# Histamine shapes the neurocomputational dynamics of human learning

**DOI:** 10.1038/s41467-026-73865-9

**Published:** 2026-06-02

**Authors:** Michael J. Colwell, Fin J. E. van Uum, Philip J. Cowen, Marieke A. G. Martens, Michael Browning, Helen C. Barron, Catherine J. Harmer, Susannah E. Murphy

**Affiliations:** 1 University Department of Psychiatry, University of Oxford, Warneford Hospital, Oxford, UK; 2 Oxford Health NHS Foundation Trust, Warneford Hospital, Oxford, UK; 3 Brain Network Dynamics Unit, Nuffield Department of Clinical Neurosciences, University of Oxford, Oxford, UK; 4 Medical Research Council Centre of Research Excellence in Restorative Neural Dynamics, University of Oxford, Oxford, UK; 5 Oxford University Centre for Integrative Neuroimaging, FMRIB, Nuffield Department of Clinical Neurosciences, University of Oxford, Oxford, UK

**Keywords:** Computational neuroscience, Cognitive neuroscience, Neurotransmitters, Learning and memory, Human behaviour

## Abstract

Histamine was the first canonical monoamine identified in the mammalian brain, yet arguably remains the least understood in its mechanistic contributions to human behaviour. Using a first-in-class causal probe (H_3_R inverse agonist pitolisant), we show how elevating histamine shapes offline and online temporal–hippocampal dynamics — sustaining episodic learning-related activity and polarising retrieval computations. Beyond this, histamine adaptively shifts neurocomputational strategy under high working memory load, while stabilising value updates during aversive reinforcement learning. These findings uncover a mechanistically grounded influence of this underexplored system on human neurocomputation, supporting its therapeutic potential in psychiatry.

## Abstract (web summary)

Histamine was the first monoamine detected in the brain, yet its role in human behaviour remains unclear. Here, Colwell et al. uncover a causal neurocomputational role for histamine in human learning.

## Introduction

Histamine was observed in the cortex nearly a century ago^1^, yet its contribution to human behaviour remains elusive compared to classical counterparts such as dopamine and serotonin. Among canonical monoamines, histamine was the first detected in the brain^1–6^, long before its neuronal organisation was mapped^7–10^. Originating from its sole nucleus (tuberomammillary nucleus [TMN]), histamine neurons project centrally with greater density than well-established systems such as noradrenaline and oxytocin^9–17^, with which histamine is known to interact^18,19^. Notably, a high proportion of histaminergic projections terminate along a conserved cortico-hippocampal pathway including the mammillary bodies – a classical structure long recognised as an essential relay for human memory^9,10,20–25^. Across this pathway, histamine neurons are controlled by their sole autoreceptor class, short isoforms of the 3^rd^ histamine receptor (H_3_R)^15,26–29^, which are expressed almost exclusively in the brain^30,31^. Histaminergic expression peaks in subcortical circuits, where it shapes synaptic plasticity and network dynamics in regions central to memory in preclinical models^23,32–40^. Within these circuits, histamine co-interacts with neuromodulators whose contribution to human learning is well-established (e.g., acetylcholine and serotonin)^41,42^. By contrast, the causal role of histamine in temporal–hippocampal dynamics and learning remains unclear.

Animal studies support a central role for histamine and H_3_R in memory, with endogenous histamine and H_3_R blockade enhancing episodic and working memory^14,39,43,44^. Additionally, extensive preclinical work implicates histamine in fear learning^45^, where targeted TMN lesions and H_3_R opto-stimulation increase aversive learning^46–49^. In humans, by contrast, evidence is sparse: preliminary PET work links H_3_R density in dorsolateral prefrontal cortex (DLPFC) with working memory^50^, and attempts to causally manipulate histamine yield non-specific neural and behavioural effects in memory paradigms^39,51^, while its role in aversive learning remains unexplored. This translational gap likely reflects the historic absence of approved methods to raise central histamine in vivo, such as blood–brain barrier–permeable H_3_R inverse agonists with greater selectivity than historical probes (e.g., betahistine)^52,53^. As the therapeutic potential of H_3_R emerges further – including its preclinical anxiolytic and antidepressant properties^54–56^ – it is becoming increasingly important to understand the broader contribution of histamine to human behaviour.

This longstanding gap may be overcome with pitolisant, a first-in-class H_3_R inverse agonist approved for narcolepsy treatment in 2019^57,58^. Notably, pitolisant is an order of magnitude more selective for H_3_R than historical compounds (e.g., > 200-fold selectivity vs. betahistine)^52,53,59,60^, with preferential activity at H_3_ autoreceptors^26–29^. Here, pitolisant increases histamine locally via autoreceptor-regulated histaminergic activity^29,53,59^, though the mode of release (*i.e*., point-to-point synaptic transmission) remains unresolved. In humans, pitolisant achieves high forebrain H_3_R occupancy ( > 80%)^61^, providing a unique opportunity to probe histamine’s causal role in human behaviour. We hypothesised that pitolisant would reflect preclinical signatures of elevated histamine, shaping temporal–hippocampal circuitry to support human learning and adaptive cognition.

Here, we leverage pharmaco-fMRI to causally reveal how histamine shapes the neurocomputational dynamics of human learning. This approach captures its influence across offline and online temporal–hippocampal learning states, and places them in broader computational contexts of working memory and reinforcement learning. In line with our predictions, the results indicate elevating histamine shifts temporal–hippocampal network dynamics, while sustaining learning-related activity and driving divergent retrieval computations. At a broader level, elevated histamine adaptively shifts neurocomputational strategy under high cognitive load, while stabilising value updates during aversive reinforcement learning.

## Results

### Histamine shapes offline temporal–hippocampal network dynamics

**Fig. 1:**
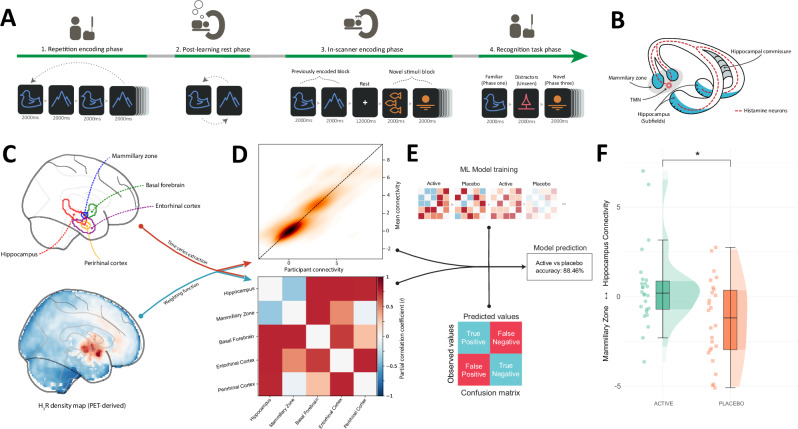
Multi-stage memory paradigm flow and modelling of H_3_R-weighted network dynamics during post-learning rest. **A** The paradigm comprised four stages: (1) encoding of visual pictures (landscapes/animals) through repeated presentation; (2) post-learning rest (10.4 mins rsfMRI) to capture network dynamics following encoding; (3) further encoding of novel (*n* = 48) and previously learned (*n* = 8) images during fMRI, enabling the novel > familiar contrast; and (4) recognition testing of previously learned (*n* = 56) and novel distractor (*n* = 27) images. **B** Histamine neurons are densely distributed along a pathway critical for memory consolidation and encoding, spanning the mammillary zone (including the TMN) to the hippocampus via the fornix^9,30^. **C** During post-learning rest (stage two), signal was extracted from memory-relevant ROIs with evidence of H_3_R sensitivity. Signals were weighted by regional H_3_R density derived from PET maps^70^. **D** Weighted signals were assembled into individual covariance matrices and **E** classified via linear discriminant analysis. Cross-validated models distinguished pitolisant from placebo with 88.5% accuracy. **F** Univariate analysis showed that network changes were driven by stronger mammillary zone ↔ hippocampus connectivity in the pitolisant group (permutation testing [pitolisant > placebo]: *t*[51] = 2.92, *p* = 0.0266, FWE-corrected, Cohen’s *d* = 0.81 [95% CI 0.24, 1.37]). Boxplots depict the interquartile range (IQR, central line = median), whiskers = ±1.5 × IQR, and half-violins show data distribution. *p ≤ 0.05, permutation test (FWE-corrected; two-tailed). **D,** **F** contain data from *N* = 52 individuals. FWE Family-wise error; ML Machine Learning.

To examine how pharmacologically elevated histamine influences the neural dynamics of both offline and online memory processing, we implemented a multi-stage task paradigm (Fig. 1A). In stage one, participants iteratively encoded images until reaching criterion (see Supplementary Note 1). Stage two comprised rsfMRI to assess how histamine modulates offline temporal–hippocampal network dynamics. In stage three, participants encoded both novel and previously learned (familiar) images during fMRI, enabling a novel > familiar contrast of new learning. Finally, in stage four, recognition memory for images learned throughout the paradigm was probed.

As temporal–hippocampal network connectivity is shaped by recent learning^62^, we investigated whether elevating histamine levels (via pitolisant) influences offline network dynamics post-learning (stage two). Signal estimates from memory-relevant regions sensitive to H_3_R manipulation^9,38,39,49,63–69^ were weighted by global H_3_R density (via *neuromaps*^70^) and used to construct covariance-based network models (Fig. 1B–D). A cross-validated machine learning classifier distinguished pitolisant vs. placebo with 88.5% accuracy (Fig. 1E). This network-level separation was explained by changes in connectivity between the hippocampus and mammillary zone—a zone containing the origin of histamine neurons^71,72^–which increased under pitolisant (Fig. 1F). Results without H_3_R-weighting showed similar group differences, with reduced effect size (Supplementary Note 2).

**Fig. 2:**
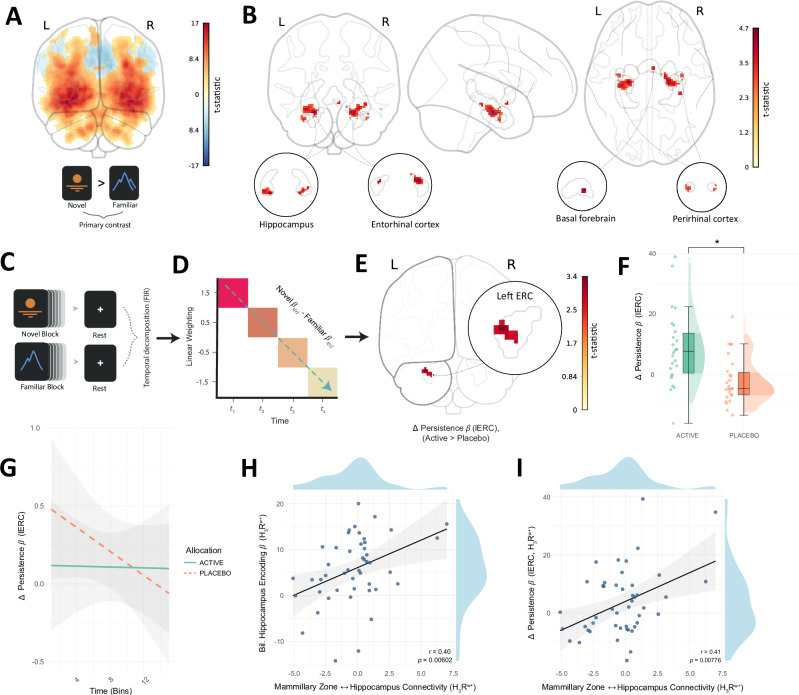
Neural dynamics of new learning and post-encoding signal persistence. During stage three of the memory paradigm, participants encoded novel images and previously learned images in separate blocks (*N* = 12 interleaved blocks), separated by post-learning rests (12,000 ms). **A** This panel depicts activity specific to new learning (novel > familiar contrast, TFCE-corrected; see Supplementary Note 3 for full statistics and coordinates). **B** Pitolisant increased activation during new learning across an a priori memory network, including bilateral hippocampal subfields, basal forebrain, and rhinal cortices (entorhinal, perirhinal; TFCE-corrected SVC). ROI outlines are shown in grey. **C,** **D** Rest periods were temporally modelled with finite impulse response functions to capture signal persistence after new learning (novel > familiar). **E,** **F** Pitolisant sustained signal persistence in the left medial entorhinal cortex (SVC permutation testing: peak MNI = -28, -12, -30; cluster = 19 voxels; *t*(51) = 3.37, *p* = 0.0222, TFCE-corrected, *d* = 1.02 [0.44, 1.60]). Boxplots depict the interquartile range (IQR, central line = median), whiskers = ±1.5 × IQR, and half-violins show data distribution. *p ≤ 0.05, SVC permutation test (FWE-corrected; two-tailed). **G** Depicts the temporal trajectory (Δ Persistence $\beta$) of signal persistence for novel > familiar activity during post-encoding rest; regression lines reflect linear fits ± SEM over time. **A**, **B**, **E**, **F**, **G** contain data from *N* = 52 individuals. **H**, **I** Hippocampal–mammillary zone connectivity during the prior rest (stage two) predicted both hippocampal encoding activity (linear regression β = 1.13, *p* = 0.00602, FWE-corrected, two-tailed; η_p_^2^ = 0.16 [95% CI 0.01, 0.35]; *r* = 0.40) and entorhinal signal persistence (linear regression β = 1.98, *p* = 0.00422, FWE-corrected, two-tailed; η_p_^2^ = 0.19 [0.03, 0.39]; *r* = 0.44). The positive associations in panels **H** and **I** were observable within allocation groups, via aggregated posterior estimates (hippocampus encoding β = 0.607, 95% range [0.606, 0.608]; Δ signal decay β = 1.19 [0.032, 2.36]). Scatterplot points depict participant mean datapoints; regression lines reflect linear fits ± SEM. **H,** **I** contain data from *N* = 47 individuals. Abbreviations: BOLD Blood Oxygenation Level Dependent; H_3_R^W+^ = Histamine 3 receptor weighted, lERC Left entorhinal cortex, ROI Region of Interest, TFCE Threshold-Free Cluster Enhancement.

Together, these findings suggest that histamine shifts offline temporal–hippocampal dynamics, providing network-level context for subsequent learning, as explored in the following section (stage three; see Fig. 2H, I).

### Histamine stabilises new learning signal persistence and leads to asymmetrical retrieval computations

Next, we investigated how pharmacologically elevated histamine influences the neural dynamics of new learning. This was tested during the third stage of the multi-stage memory paradigm, where participants encoded novel images alongside those previously learned in stage one. Neural activity was contrasted between these conditions (novel > familiar; Fig. 2A; Supplementary Table 3), allowing the isolation of neural dynamics specific to new learning. During new learning, pitolisant increased activation in bilateral hippocampal subfields in both whole-brain and small-volume corrected (SVC) analyses (Fig. 2B; Supplementary Note 3; Supplementary Table 4). Increased activity was also observed across the a priori memory network from the preceding rest stage, including basal forebrain, entorhinal cortex and perirhinal cortex. Stimulus discrimination behaviour (accuracy and response time) did not differ significantly between groups, indicating comparable task engagement (Supplementary Note 4).

As persisting temporal–hippocampal activity during post-learning rest is linked to effective consolidation in humans^73–75^, we asked whether histaminergic modulation following pitolisant administration influences this process. To test this, signal from post-encoding rest intervals was temporally decomposed using finite impulse response (FIR) modelling, enabling us to capture signal persistence after new learning (novel > familiar; Fig. 2C, D). Pitolisant sustained temporal persistence for new learning in the left medial entorhinal cortex (Fig. 2E, F). This effect reflects persistence beyond initial response magnitude, as shown by the FIR-isolated decay trajectories (Fig. 2G). Importantly, encoding-related activity in the left hippocampus and entorhinal cortex—but not their right-hemisphere homologues—predicted entorhinal persistence, suggesting a lateralised effect (Supplementary Note 5). Moreover, offline hippocampal–mammillary zone connectivity (stage two) was associated with higher hippocampal activity during encoding (Fig. 2H) and increased entorhinal signal persistence (Fig. 2I). These associations were absent or weakened when H_3_R-weighting was removed (Supplementary Table 9), supporting histaminergic specificity.

Thirty minutes after in-scanner encoding (stage three), recognition memory for images learned throughout the paradigm was probed (stage four). Choice behaviour was modelled with a drift diffusion model (DDM) of evidence accumulation. Parameter estimation was stimulus-dependent, as previously encoded images and unseen distractors are assumed to differ in their evidence accumulation properties^76^. Full details of model selection, parameter recoverability, and posterior predictive checks are provided in the Supplementary Methods. The best-fitting model included four stimulus-dependent parameters, formalising distinct parts of choice behaviour: drift rate (${v}_{s}$), reflecting evidence accumulation efficiency; decision policy (${a}_{s}$), reflecting the evidence threshold required before making a decision, capturing signal-noise separation; non-decision time (${t}_{{er},s}$), reflecting the pre-decisional buffer between stimulus onset and evidence accumulation; and starting point bias (${z}_{s}$), reflecting an initial bias toward one response boundary.

**Fig. 3:**
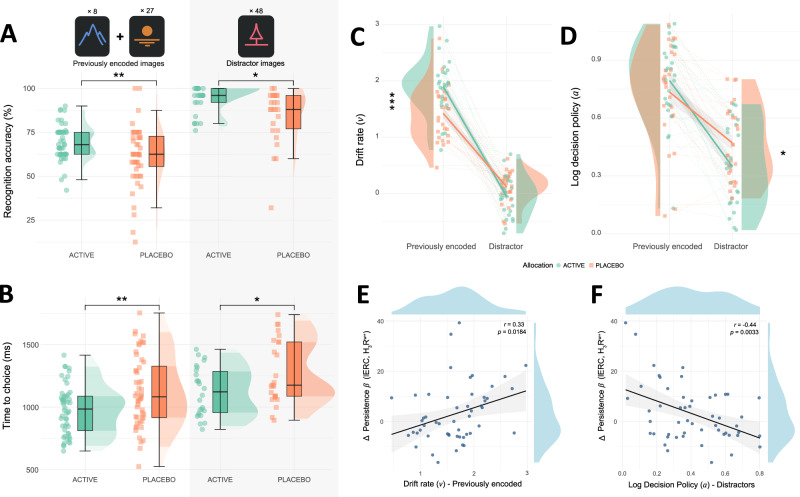
Decision computations during memory recognition and their association with prior encoding-related activity. The multi-stage memory paradigm concluded with a memory recognition task. Test items comprised previously encoded images (stage one: *n* = 8; stage three: *n* = 27) and unseen distractors (*n* = 48). **A** Recognition accuracy was higher under pitolisant (distractors EMM = 8.62 ± 3.71, two-tailed *p* = 2.187610e-02, *d* = 0.76 [0.11, 1.40]; previously encoded EMM = 7.50 ± 2.69, two-tailed *p* = 1.346737e-02, *d* = 0.67 [0.13, 1.18]). **B** Time to correct choices was reduced under pitolisant (distractors EMM = -154.0 ± 66.0, two-tailed *p* = 2.144859e-02, *d* = -0.81 [-1.50, -0.12]; previously encoded EMM = 144.0 ± 54.5, two-tailed *p* = 1.034688e-02, *d* = -0.76 [-1.34, -0.18]). **C** Drift rate (${{{{\boldsymbol{v}}}}}_{{{{\boldsymbol{s}}}}}$) was higher under pitolisant for previously encoded images (EMM = 0.48 ± 0.11, *p* = 6.292987e-05, *d* = 1.16 [0.59, 1.73]) but not distractors (EMM = -0.42 ± 0.28, two-tailed *p* = 1.307893e-01, *d* = -0.42 [-0.97, 0.13]). **D** Decision policy (log ${{{{\boldsymbol{a}}}}}_{{{{\boldsymbol{s}}}}}$) was reduced for distractors under pitolisant (EMM = -0.12 ± 0.06, two-tailed *p* = 4.550284e-02, *d* = -0.71 [-1.42, -0.01]), with no effect for previously encoded items (EMM = 0.06 ± 0.06, two-tailed *p* = 3.195107e-01, *d* = 0.35 [-0.35, 1.05]). **E** lERC signal persistence predicted drift rate for previously encoded items (linear regression β = 0.02, *p* = 0.0368, FWE-corrected, two-tailed; η_p_^2^ = 0.11 [0.00, 0.28]; *r* = 0.33), but not for distractors (β = -0.0005, two-tailed *p* = 0.897). **F** lERC persistence negatively predicted decision policy for distractors (linear regression β = -0.008, *p* = 0.0033, FWE-corrected, two-tailed; η_p_^2^ = 0.19 [0.04, 0.38]; *r* = -0.44), but not previously encoded items (β = 0.0005, two-tailed *p* = 0.838). The direction of effects in **E** and **F** was consistent across stimulus types (adjusted for groups), confirmed by posterior estimates (${{{{\boldsymbol{v}}}}}_{{{{\boldsymbol{s}}}}}$ β = 0.006 [0.004, 0.008]; log ${{{{\boldsymbol{a}}}}}_{{{{\boldsymbol{s}}}}}$ β = -0.004 [-0.007, -0.0006]). **A**–**F** contain data from *N* = 52 individuals. **A**, **B** Boxplots depict the interquartile range (IQR, central line = median), whiskers = ±1.5 × IQR, and half-violins show data distribution. **C**, **D** Points/violins show data distribution; bold lines depict group means. *** *p* ≤ 0.001, ** *p* ≤ 0.01, * *p* ≤ 0.05, two-tailed EMM tests (FWE-corrected). **E**, **F** Scatterplot points depict participant mean datapoints; regression lines reflect linear fits ± SEM. Abbreviations: H_3_R^W+^ = Histamine 3 receptor weighted, ms milliseconds, lERC Left entorhinal cortex, TFCE Threshold-Free Cluster Enhancement.

Behaviourally, pitolisant improved recognition accuracy (mean %; ANOVA main effect of group: F[1,50] = 8.72, *p* = 0.0048, η_p_^2^ = 0.13 [95% CI 0.01, 0.31]) and reduced time to choice (ms; ANOVA main effect of group: F[1,50] = 7.90, *p* = 0.00703, η_p_^2^ = 0.13 [0.01, 0.31]) across stimulus types (Fig. 3A, B). Computationally, pitolisant increased drift rate for previously encoded images (${v}_{s}\,$ANOVA group × stimulus interaction: F[1,50] = 14.16, *p* = 0.000441, η_p_^2^ = 0.14 [0.04, 0.27]; ${v}_{s}$ ANOVA main effect of group: F[1,50] = 4.13, *p* = 0.0473, η_p_^2^ = 0.03 [0.00, 0.13]; Fig. 3C). Conversely, pitolisant reduced decision policy for distractors only (log ${a}_{s}\,$ANOVA group × stimulus interaction: F[1,50] = 7.36, *p* = 0.00912, η_p_^2^ = 0.13 [0.01, 0.31]; log ${a}_{s}$ main effect of group: F[1,50] = 0.38, *p* = 0.538, η_p_^2^ < 0.01 [0.00, 0.12]; Fig. 3D). No significant group differences emerged for other parameters (Supplementary Fig. 7). Post-encoding signal persistence (lERC; stage three) was positively associated with drift rate for previously encoded images but not distractors (Fig. 3E). Conversely, persistence negatively predicted decision policy for distractors, but not previously encoded items (Fig. 3F).

Taken together, these results indicate that histamine enhances entorhinal signal persistence following learning, while during retrieval it drives asymmetrical computations—selectively enhancing evidence accumulation for past learning and lowering the evidence required for unfamiliar information. More broadly, our findings suggest a histaminergic shaping of temporal–hippocampal dynamics across offline and online learning states in support of consolidation.

### Histamine influences the neurocomputational signatures of working memory

**Fig. 4:**
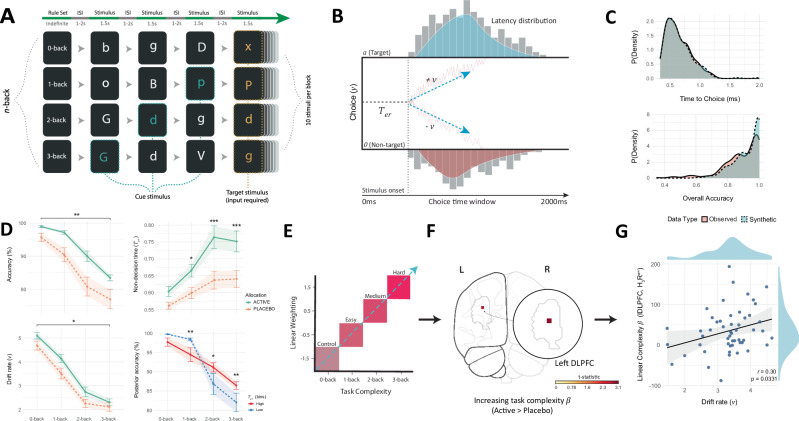
Task procedure, computational modelling and analysis of the complex working memory task. **A** The verbal *n*-back task included four levels of increasing complexity (0–3 back; rows), with each requiring cued information to be maintained over longer intervals. Before each block, participants were cued with task rules (e.g., press right if the current letter matches that two trials ago). Each level comprised four blocks (16 total), with 20,000 ms rests interleaved. **B** Drift diffusion models were fit to choice behaviour (4 chains × 4,000 samples per *N*), containing three parameters: drift rate ($+v/-v$), non-decision time (${T}_{{er}}$) and decision policy ($a$). **C** Models fit to individual participants resulted in posteriors which closely matched observed behaviour. **D** Pitolisant increased accuracy (upper left) and drift rate (lower left) independent of task load, while non-decision time increased as a function of load (upper right). Splitting trials by high vs. low ${T}_{{er}}$ (within-subjects median split) revealed that higher *T*_*er*_ decreased accuracy under low load, but improved accuracy at high load (lower right). Plot points depict mean value; error bars and shaded area depict SEM. ***p ≤ 0.001, **p ≤ 0.01, *p ≤ 0.05, two-tailed EMM tests (FWE-corrected*;* see Supplementary Note 6 for full statistics). **E** BOLD signal was estimated as a function of task complexity via parametric modelling. **F** Pitolisant increased recruitment of the left DLPFC as task complexity increased (SVC permutation testing: peak MNI = -34, 38, 34; cluster = 5 voxels; *t*(51) = 3.12, *p* = 0.0456, TFCE-corrected, *d* = 0.93 [0.35, 1.50]). **G** Linear activity within this cluster predicted drift rate ${{{\boldsymbol{v}}}}$ (linear regression β = 0.004, *p* = 0.0335, two-tailed; η_p_^2^ = 0.09 [0.00, 0.26]; *r* = 0.30); this relationship was observable within allocation groups, based on aggregated posterior estimates (β = 0.003 [0.001, 0.005]). Scatterplot points depict participant mean datapoints; regression lines reflect linear fits ± SEM. **C, D,** **F** and **G** contain data from *N* = 52 individuals. Abbreviations: lDLPFC = Left Dorsolateral Prefrontal Cortex; H_3_R^W+^ = Histamine 3 receptor weighted; TFCE = Threshold-Free Cluster Enhancement.

We next extended our investigation from episodic learning to working memory, asking how pharmacologically elevated histamine shapes its neurocomputational signatures. Participants performed an fMRI-adapted verbal *n*-back task (Fig. 4A), indicating whether a target letter had appeared *n* trials earlier (0-back [control], 1-back [easy], 2-back [moderate], 3-back [hard]). This stepwise design enabled the modelling of load-dependent changes in decision computations and neural activity. As with the memory recognition task, choice behaviour was modelled using a drift diffusion model (DDM; Fig. 4B). The winning model was selected based on goodness-of-fit among candidate models and parameter recoverability (Supplementary Methods), and validated by posterior predictive checks (Fig. 4C). The final model included three parameters: drift rate ($+v/-v$), non-decision time (${T}_{{er}}$), and decision policy ($a$).

Behaviourally, pitolisant improved overall task accuracy (main effect of group ANOVA: F[1,50] = 7.89, *p* = 0.0070, η_p_^2^ = 0.14 [0.01, 0.32]; Fig. 4D, upper left), but did not significantly change time to choice (Supplementary Fig. 9A). Computationally, pitolisant increased drift rate ($+v/-v$) independent of task load (main effect of group ANOVA: F[1,50] = 4.94, *p* = 0.0308, η_p_^2^ = 0.09 [0.00, 0.26]; Fig. 4D, lower left), while no significant group effect on decision policy ($a$) was observed (Supplementary Fig. 9B). Non-decision time (${T}_{{er}}$) increased with task complexity under pitolisant (ANOVA group × task condition interaction: F[3,150] = 2.70, *p* = 0.0481, η_p_^2^ = 0.05 [0.00, 0.11]; Fig. 4D, upper right). To assess its contribution to behaviour, we split posterior performance into high and low ${T}_{{er}}$ bins (500,000 draws; see Supplementary Methods). High ${T}_{{er}}$ reduced accuracy at low load but enhanced it at high load (ANOVA group × task condition interaction: F[3, 158] = 2.78, *p* = 0.043, η_p_^2^ = 0.08 [0.01, 0.15]; Fig. 4D, lower right), consistent with confirmatory analyses of observed accuracy (Supplementary Fig. 9C; Supplementary Note 7). Under higher working-memory load, encoding demands additional resources^77^, and increases in ${T}_{{er}}$ may reflect a strategic reallocation of pre-decisional time to support these processes. Importantly, such slowing of ${T}_{{er}}$ can reflect adaptive shifts in cognitive strategy, independent of visuomotor deadtime^78^.

In a parametric model capturing neural responses to increasing task load (Fig. 4E), pitolisant increased recruitment of the left dorsolateral prefrontal cortex (Fig. 4F; Supplementary Fig. 11). Load-related activity in this region tracked drift rate ($v$; Fig. 4G). Notably, these effects were absent when H_3_R-weighting was removed from parameter estimates (Supplementary Table 9), supporting histaminergic specificity. Furthermore, drug-related increases in neural recruitment peaked at moderate task load (2 > 0-back), where DLPFC activity plateaus (Supplementary Fig. 12), encompassing left DLPFC, bilateral hippocampus, and substantia nigra (in both whole-brain and SVC analyses; Supplementary Figs. 10, 11, 13; Supplementary Tables 6–7).

Overall, these findings indicate that histamine promotes adaptive shifts in neurocomputational strategy under high cognitive loads. Specifically, the interval preceding evidence accumulation lengthens (${T}_{{er}}$), while recruitment of the left dorsolateral prefrontal cortex increases – a region which tracks evidence accumulation efficiency ($v$), as seen here and in previous causal interference work^79,80^.

### H_3_R blockade influences aversive computations during reinforcement learning

**Fig. 5:**
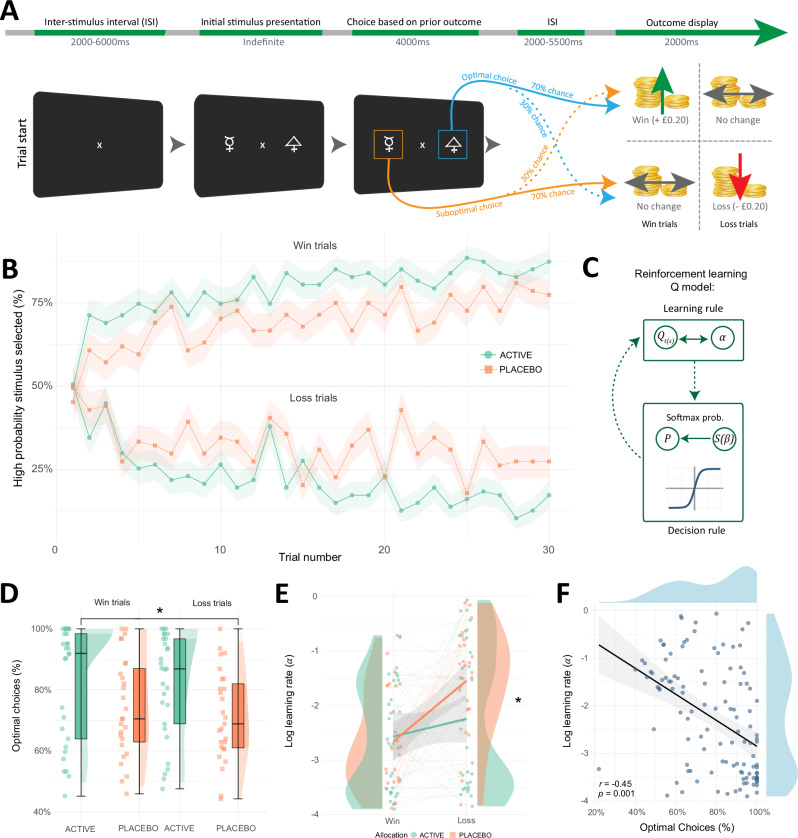
Reinforcement learning procedure, computational modelling and causal analyses. **A** Probabilistic instrumental learning task. Participants chose between symbols within a pair, with two interleaved pairs representing win and loss conditions. Symbols had fixed reciprocal probabilities (70% vs. 30%) of favourable outcomes (winning or avoiding loss). Learning occurred across 60 trials (30 per condition) over three blocks. Optimal performance required repeated selection of the high-probability symbol; cumulative earnings were paid at study completion. **B** Learning over time across groups (averaged across blocks). The Y-axis (high probability stimulus selected) refers to the selection (mean %) of high probability win or loss symbols. The shaded region surrounding the lines indicates the standard error. **C** Choice behaviour was fit to a Q-learning model^81–83^, comprising a learning rule which updates value estimates (${Q}_{t(s)}$), and decision rule assigning choice probability ($P$) via softmax. Two parameters, learning rate ($\alpha$) and inverse decision temperature ($\beta$), formalise distinct computational processes contributing to task behaviour (for further details, see Supplementary Methods). **D** Pitolisant increased overall optimal choices (win trials EMM = 9.57 ± 4.62, *p* = 4.107559e-02, *d* = 0.74 [0.23, 1.46]; loss trials EMM = 11.37 ± 4.62, *p* = 1.563904e-02, *d* = 0.88 [0.16, 1.60]). Boxplots depict the interquartile range (IQR, central line = median), whiskers = ±1.5 × IQR, and half-violins show data distribution. **E** Pitolisant reduced learning rate (log $\alpha$) during loss trials (win trials EMM = 0.22 ± 0.30, *p* = 4.588790e-01, *d* = 0.24 [-0.39, 0.86]; loss trials EMM = -0.56 ± 0.26, two-tailed *p* = 3.643643e-02, *d* = -0.59 [-1.15, -0.03]). Points/violins show data distribution; bold lines depict group means. **F** Learning rate (log $\alpha$) was negatively associated with optimal choices (β = -4.32, two-tailed *p* = 2.31e-05, η_p_^2^ = 0.16 [0.05, 0.29); *r* = -0.45); this relationship was observable within allocation groups, based on aggregated posterior estimates (β = -6.70 [-7.13, -6.26]). Scatterplot points depict participant mean datapoints; regression lines reflect linear fits ± SEM. **C**–**F** contain data from *N* = 57 individuals. * *p*  ≤  0.05, two-tailed EMM tests (FWE-corrected). ISI Inter-stimulus interval.

Next, we investigated whether pharmacologically elevating histamine influences reinforcement learning using a probabilistic instrumental learning task (Fig. 5A). Throughout the task, participants chose between symbol pairs associated with stable outcome probabilities, in either rewarding (monetary gain) or aversive (monetary loss) contexts. Optimal performance required selecting the symbol with the higher probability (70%) of favourable outcome (winning money or avoiding loss). Participant choices throughout the task were fit to a computational Q-learning model (Fig. 5B) adapted from refs. ^81–83^ (see Supplementary Methods for model specifications and validation procedures). The model formalises learning and decision computations with two parameters: learning rate ($\alpha$) which describes the rate at which expectations are updated during learning; and inverse decision temperature ($\beta$), which captures the degree of stochasticity during decision-making. As $\alpha$ and $\beta$ covary, inferential analyses incorporated the reciprocal parameter in the linear model structure (see Supplementary Methods for further details).

Throughout the task, pitolisant increased optimal choices (Fig. 5C,D; main effect of group ANOVA: F[1,55] = 7.08 *p* = 0.0102, η_p_^2^ = 0.11 [0.01, 0.28]), but did not significantly change time to choice (Supplementary Fig. 14A). Computational modelling showed that pitolisant reduced the learning rate $\alpha$ during loss trials (Fig. 5E; ANOVA group × task condition interaction: F[1,55] = 4.29, *p* = 0.041963, η_p_^2^ = 0.06 [0.00, 0.19]; main effect of group ANOVA: F[1,55] = 0.68, *p* = 0.413), but did not significantly change inverse decision temperature $\beta$ (Supplementary Fig. 14B; Supplementary Note 8). Moreover, these effects persisted when the first ten trials per condition were excluded from the computational model, indicating they were not driven by early exploratory noise (Supplementary Note 8). Notably, both observed optimal choices and model-derived (posterior) optimal choices were associated with lower $\alpha$ (Fig. 5F; Supplementary Fig. 14C); consistent with the principle that, in stable environments, lower $\alpha$ is optimal given that prior outcomes are more predictive of future uncertainty^84,85^. Together, these results suggest histamine may adaptively shift value updating toward a more stable (or, less reactive) state in aversive contexts.

### Comparative H_3_R-weighting, cerebral blood perfusion, blinding integrity, and subjective affect

Additional control analyses were performed to examine H_3_R specificity and potential confounds in the primary analyses. Applying H_3_R-weighting to task-based fMRI clusters (pitolisant > placebo) increased the magnitude of group-level effects (mean ± SE Δ*d* = +0.12 ± 0.05), with peak correspondence in the basal forebrain during new learning (Δ*d* = +0.34; Supplementary Fig. 15). Cerebral blood perfusion did not differ significantly between groups, either globally (grey and white matter) or regionally within task-specific networks (Supplementary Note 9; Supplementary Fig. 16).

Blinding integrity was assessed via participants’ estimation of group allocation and side-effect profiles. Group allocation estimates did not differ across groups at study completion (χ² = 0.00, *p* = 1.00, *N* = 58), with both groups predominantly guessing placebo (79%). Side-effect profiles did not differ significantly during acute administration (Supplementary Table 10).

As subjective affect and cognition (e.g., motivation, arousal) can indirectly influence task behaviour^86^, we assessed repeated measures of these domains. No significant group differences were observed over the testing period (Supplementary Fig. 17; Supplementary Tables 11, 12). An exploratory analysis found that task performance and subjective cognition were not significantly related (Supplementary Table 13), consistent with previous clinical work^87,88^. Before testing, no significant group differences were observed in self-reported measures of sleep, fatigue, alertness, or arousal (Supplementary Note 10).

## Discussion

Histamine has arguably remained the least understood of the canonical monoamines, despite being the first identified in the mammalian brain^1–6^. The present findings help close this gap, revealing the causal role of histamine in the neurocomputational architecture of human learning. Together, these findings underscore histamine’s translational potential, especially in conditions characterised by cognitive impairment, where augmenting histaminergic signalling may counteract pathophysiological mechanisms^89–92^.

Pharmacologically elevated histamine activity shifted temporal–hippocampal dynamics within a H_3_R-weighted network. This network-level separation was isolated to strengthened functional coupling between the hippocampus and mammillary zone – this zone encompasses both the histamine nucleus (TMN) and mammillary bodies, which share local circuitry and exhibit among the highest histaminergic fibre densities brain-wide^9,93,94^. Notably, the mammillary bodies appear to function as a mediator of downstream histamine release in the hippocampus^95–97^. The strength of hippocampal-mammillary zone coupling predicted both online encoding-related activity within the hippocampus, and post-encoding signal persistence in the entorhinal cortex. Given histamine primes hippocampal CA3 sharp-wave activity^98^, this raises the possibility that hippocampal-mammillary zone coupling may drive broader cortico-hippocampal dynamics in support of retrieval and consolidation.

Within the same temporal–hippocampal network where increased offline functional coupling was observed, elevated histamine increased neural recruitment during new learning, and sustained post-learning activity within the medial entorhinal cortex (MEC). MEC neurons exhibit persistent activity which directly modulates cortico-hippocampal activity^99,100^, and disruption of MEC inputs during the post-learning period impairs memory formation^101^. As histamine enhances theta-coupled spiking within the MEC^38^, this points to a role for histamine in driving consolidation through sustained MEC signal persistence. Indeed, we observed that higher levels of MEC signal persistence predicted future retrieval computations, which in turn were asymmetrically shifted in those with pharmacologically elevated histamine levels (selectively enhancing evidence accumulation for previously encoded items, while reducing decision policy strength for distractors).

Learning systems interface with broader domains of cognition; accordingly, we probed the broader effects of elevated histamine on working memory and reinforcement learning. Under higher working-memory load, a shift in both computational strategy and dorsolateral prefrontal recruitment occurred in those receiving pitolisant. Within the deep laminar layers of the prefrontal cortex where H_3_R is expressed^102^, histamine release from TMN-projecting neurons regulates fast-spiking interneurons essential for aspects of working memory function^103–105^. Potentially, H_3_R blockade via pitolisant shifted constitutive receptor activity within this network^29,106^, contributing to the present findings. Accordingly, our findings complement but reverse the direction of prior human correlation work showing that greater DLPFC H_3_R density is associated with reduced *n*-back performance^50^.

During reinforcement learning (RL), pharmacologically elevated histamine flattened learning rates for aversive outcomes. Preclinically, activation of H_3_R via optogenetics and TMN lesions produces the behavioural inverse of the present findings^46–49^, while suppression of H_3_R via antagonism impairs aversive memory consolidation^107^. These effects suggest that histamine influences aversive learning via circuits partly distinct from hippocampal-dependent memory, potentially through parallel engagement of cholinergic pathways^13,49,107,108^. Accordingly, we found no relationship between RL parameters ($\alpha$, $\beta$) and evidence accumulation efficiency $v$ during memory retrieval (Supplementary Note 8), consistent with these effects arising from distinct computational mechanisms.

A common thread across the present findings is that histamine appears to bias neurocomputation toward stability and adaptive evidence accumulation, consistent with proposals that histamine supports novelty-linked arousal rather than undifferentiated global arousal^30,109,110^. Indeed, microdialysis-EEG work suggests elevated histamine (via pitolisant) is not accompanied by general increases in arousal/wakefulness^111^. By selectively stabilising encoding-related activity and dampening reactive value updating, histamine may confer computational advantages in environments where relevant information must be retained and distractors filtered. However, as with other monoamines^82,112–114^, histaminergic potentiation may incur broader costs, including anxiety and insomnia^15,115,116^, counterbalancing its adaptive gating of novelty-linked arousal.

The principal mechanism of pitolisant is presumed to be upregulation of central histamine via autoreceptor blockade^53,57,117^, where it shows minimal intrinsic activity at H_3_R isoforms believed to function as heteroreceptors^28,29,118^ (see Supplementary Note 11 for further discussion). While it is not possible in humans to fully exclude downstream contributions of other neuromodulatory systems (e.g., GABA, noradrenaline, dopamine, and serotonin), this is largely due to constraints that preclude polypharmacy blockade approaches (*e.g*., H_1_R antagonism) in healthy volunteers. Specifically, combining multiple centrally active agents in healthy volunteers introduces safety, regulatory, and interpretational constraints that limit the use of polypharmacy designs.

For the present work, convergent preclinical evidence supports a predominantly histaminergic mechanism. In rodents, the H_3_R inverse agonist ciproxifan enhances recognition memory, and this effect is abolished by H_1_R and H_2_R antagonists^119^. Moreover, chemogenetic activation of histaminergic neurons in the TMN improves object recognition and spatial memory, while H_2_R antagonism reverses these effects^66^. Furthermore, histamine can modulate hippocampal synaptic transmission via H_3_R even when GABA_A_ receptors are pharmacologically blocked^120^, indicating that histaminergic effects do not require GABAergic mediation. Additionally, GABA co-released from TMN projections produces tonic inhibition of pyramidal neurons^103^, which would be expected to suppress, rather than enhance, learning-related neural activity. Indeed, baseline GABA levels are inversely related to BOLD reactivity^121^. This contrasts with the learning-associated increases in BOLD signal observed across tasks in the present study. Nonetheless, future work will be important to determine the relative contribution of H_3_ autoreceptors and heteroreceptor variants, as well as the possible causal involvement of adjacent monoaminergic systems^122^.

The present findings may prove relevant for efforts to therapeutically target H_3_R in disorders marked by cognitive impairment^89–91^. In Alzheimer’s disease, reductions in histaminergic neuron number and HDC mRNA expression^17,123,124^ support a biologically plausible rationale for H_3_R blockade. Depression has also been linked to alterations in histaminergic signalling, including reduced cortical H_1_R binding^125^; however, postmortem studies suggest that histaminergic neurons are largely preserved in depression^126^, indicating a more subtle or indirect involvement. Moreover, the effects of H_3_R blockade in clinical populations such as Alzheimer’s disease and schizophrenia have been mixed, ranging from modest benefits^127–129^ to no clear efficacy^130–133^. Many prior studies used H_3_R antagonists or weak inverse agonists, which differ from pitolisant in their suppression of constitutive H_3_R signalling, particularly at autoreceptor-associated isoforms^29^, potentially contributing to inconsistent clinical outcomes. Accordingly, future mechanistic work with H_3_R inverse agonists, which may include compounds such as bavisant (JNJ-31001074) and AZD5312, may endeavour to delineate isoform-specific engagement and downstream cognitive effects.

In conclusion, the present study highlights the broad mechanistic contribution of a long-neglected classical neurotransmitter system – histamine – to human behaviour. Specifically, we show that increasing histamine activity shapes offline and online hippocampal dynamics, stabilising entorhinal activity post-learning and shifting future retrieval computations. More broadly, elevated histamine signalling adapts neurocomputational strategy under high working-memory load while flattening learning rates for aversive outcomes. Together, these findings position histamine as a mechanistic entry point for influencing neurocomputation, motivating further study of its translational potential.

## Methods

### Participants and design

Sixty healthy participants met the study eligibility and were randomised to receive a single dose of pitolisant or placebo (*N* = 29:31, pitolisant:placebo). Study recruitment and data collection was undertaken between April 2023 and January 2024. To assess eligibility to participate, prospective participants underwent two screening sessions: an initial pre-screening (online) and subsequent in-person medical screening. Exclusion criteria included medical conditions which were contraindicated for pitolisant (e.g., hepatic impairment), history of neurological or psychiatric health concerns, recent recreational drug use (3 month wash-out), neuroimaging contraindications, recurrent use of medications with histaminergic properties (e.g., antihistamines for allergies; antidepressants or antipsychotics, including mirtazapine and clozapine), and current pregnancy or breastfeeding. All participants had a BMI between 18-31 and were fluent in English. To avoid functional (fMRI) confounds associated with handedness (*i.e*., left- or right-handed) during cognitive memory performance^33^, only right-handed individuals were included. Full study inclusion and exclusion criteria are described within Supplementary Table 1. For details of participant flow through recruitment and randomisation procedures, see the CONSORT flow chart (Supplementary Fig. 1). The final sample consisted of 58 healthy individuals (37 females; drug:placebo = 29:29; mean age = 28.17) and were well matched across baseline working memory (digit span), education (years and level of attainment), and other demographic factors (Supplementary Table 2).

Participants were randomised to receive a single high therapeutic dose of H_3_R inverse agonist pitolisant hydrochloride 36 mg (18 mg × 2 film-coated tablets) or placebo (lactose tablets), in a double-blind study design. As the experimental design centred on a multi-stage learning paradigm, a within-subject crossover was avoided as repeating the four-stage learning cycle may introduce meta-learning and period effects^134,135^. Both pitolisant and placebo were encapsulated in a generic opaque capsule to conceal allocation from researchers and participants. Encapsulation and randomisation were undertaken by external researchers not engaged in data collection at the NIHR Oxford Health Biomedical Research Centre (Oxfordshire, United Kingdom).

Randomisation was undertaken using a novel variance minimisation algorithm which allows minimising potential group-level differences in general cognitive function at baseline^136^. The algorithm balances group allocation while accounting for covariates—gender and baseline cognition score (calculated using the Wechsler Adult Intelligence Scale digit span aggregate^137^)—using a computed sum of squared deviations.

The study was assessed and approved by the Central University Ethics Committee at the University of Oxford (MSD-IREC reference code: R83940/RE002). All participants provided informed consent prior to study participation. The study and its protocol were pre-registered on the National Institute of Health Clinical Trials Database (NCT05849675). All pre-registered primary outcomes are presented in this paper. Screening and study visits were conducted within the Department of Psychiatry and Oxford Centre for Human Brain Activity, University of Oxford.

### Procedure

Participants undertook an online pre-screening procedure to assess basic eligibility (*i.e*., BMI, medical status and handedness) before being invited to an in-person medical screening. During the in-person screening, participants were administered the Structured Clinical Interview for DSM-V to screen for current or past psychiatric illness. Medical history, medications, and MRI contraindications were assessed in interview. Blood pressure and heart rate were taken, and urine samples were acquired to assess current pregnancy and/or drug use. During this visit, participants undertook the Wechsler’s Digit Span test (forward, backward and ordered)^137^, an index of general intelligence/cognition^138–141^, which allowed balancing general cognitive functioning across allocation groups (via variance minimisation randomisation).

Gender was determined by self-report. Although gender was considered in the study design — with groups balanced for sex via the variance minimisation randomisation procedure — gender-disaggregated analyses were not conducted as the study was not powered to detect gender differences in the primary outcomes.

Eligible participants who passed the screening process were invited to the study visit, where the main study outcome data was collected. At the start of the study visit, participants completed baseline questionnaire battery of affect (for further details on questionnaire measures, see Supplementary Methods). Following this, participants self-administered their assigned intervention (pitolisant or placebo) in the presence of a researcher. All study visits followed a fixed schedule, with drug administration at approximately 09:00-10:00 for all participants, thereby controlling for known circadian fluctuations in histaminergic tone^142,143^. Next, there was a three-hour period to allow drug metabolism before commencing neuroimaging and behavioural experiments. After three hours, pitolisant reaches peak serum concentration and high occupancy of brain-wide H_3_R within the high therapeutic dose range used within the present study^61,144^. This three-hour interval falls well within the reported elimination half-life of pitolisant (approximately 11 hours^145^), supporting sustained central bioavailability throughout the testing period. After three hours, participants undertook neuroimaging, behavioural testing and questionnaire procedures at the Oxford Centre for Human Brain Activity. At the end of the study visit, participants were asked to estimate their allocation before study debriefing. Participants were reimbursed 140 GBP upon completion of the study.

### fMRI memory and learning task paradigms

The multi-stage memory paradigm (Fig. 1A; adapted from ref. ^146^) is composed of four distinct stages. First, participants engage in the encoding of visual stimuli (images of landscapes or animals) through repetitive encoding. To support encoding, participants categorised images by content via button press (A for animal; L for landscape). In the second stage, resting-state fMRI (rsfMRI) was undertaken over a 10.4 min interval to capture network dynamics during the post-learning stage. The third stage involves a second encoding task similar to the first; however, it incorporates both previously learned images (*n* = 8) and novel images (*n* = 48) within an fMRI scanning session. This design enables direct comparison of neural responses associated with the encoding of new information versus familiar content (i.e., novel > familiar contrasts); task sequences were optimised to probe the hippocampal subfields (dentate gyrus and cornu ammonis [CA1-3]) given their importance in memory formation^42,146^. Finally, the fourth stage comprises a memory recognition task in which participants are asked to determine whether each presented image was encountered earlier in the experiment (from either the first or third stage; *n* = 56) or represents a novel distractor (*n* = 27). During this task, participants indicate if images were previously seen via button response (A for previously seen; L for unseen distractors). For the memory recognition task, behavioural task outcomes (accuracy and response time) were used to undertake drift diffusion modelling of evidence accumulation (see Supplementary Methods for further details). Behavioural, computational and neuroimaging data for tasks during the multi-stage memory paradigm consisted of *N* = 52 individuals (mean age = 28.58; 33 female), while resting state neuroimaging data consisted of *N* = 52 individuals (mean age = 28.39; 35 female), reflecting independent quality-based exclusions applied to each dataset.

The complex working memory task (verbal *n*-back; Fig. 4A) captures neural activity during working memory processing under increasing task complexity, inducing activation changes within the DLPFC, hippocampus and dopaminergic nuclei^147,148^. During the task, participants must identify if a letter corresponds to a previously presented letter according to a rule. Specifically, if the letter occurred one trial ago (1-back), two trials ago (2-back), or three trials ago (3-back). In addition, there was an intermixed sensorimotor control task which served as a performance baseline (0-back). Letters appeared interleaved in sets of ten trials per block with four blocks per task condition (16 task blocks and 17 rest blocks total). To deter phonological and visual task strategy, occlusive consonants served as task stimuli in both upper and lower case form. The behavioural task outcomes were adjusted overall accuracy (correct hits—false alarms) and response time. Additionally, behavioural data for the task was fit to a computational model of evidence accumulation, a drift diffusion model, which was validated here through model selection, parameter recovery, and posterior predictive checks (see Supplementary Methods for further details). The winning model contained the following parameters: drift rate (+*v/-v*), non-decision time (*T*_*er*_) and decision policy (***a***). This task, associated preprocessing scripts, and computational modelling scripts are shared via an open-source repository: https://github.com/mjcolwell/n-back_oxford. Behavioural, computational and neuroimaging data for the complex working memory task consisted of *N* = 52 individuals (mean age = 28.12; 33 female).

The Probabilistic Instrumental Learning Task (Version 220916; adapted from^81,83^) assesses reward and loss-based instrumental learning using a fixed-probability outcome structure. Participants chose between two symbols in alternating pairs: one pair associated with high-probability reward trials (outcomes: +£0.20 or no change), and the other with high-probability loss trials (outcomes: –£0.20 or no change). Each symbol within a pair was associated with a fixed outcome probability (70% vs. 30%), and trial outcomes were presented immediately following the choice. Participants were informed prior to the task that their cumulative earnings would be awarded upon study completion and were instructed to maximise monetary gain. Primary behavioural outcomes included the proportion of optimal choices (i.e., selecting the option with the higher probability of gain or loss avoidance) and response time (ms). In addition, computational Q-learning models were fit to choice behaviour, allowing access to parameters (learning rate $\alpha$; inverse decision temperature $\beta$) which formalise decision learning and computations throughout the task (for further details, see Supplementary Methods). Behavioural and computational data for the instrumental learning task consisted of *N* = 57 individuals (mean age = 28.16; 36 female).

### Behavioural and demographic data analysis

Behavioural task and demographic data pre-processing and statistical analyses were carried out using R Software (version 4.3.1)^149^. The effect of group allocation (pitolisant vs. placebo) on behavioural task outcomes was undertaken via two-way mixed-effect ANOVA modelling, with group and task condition serving as fixed factors and participant as a random factor; the variance minimisation procedure allows for ANOVA analysis without inclusion of balancing covariates^136^. Planned comparisons were undertaken via two-tailed estimated marginal means analysis (EMM), where estimates are reported with associated standard means error. Family-wise error (FWE) was corrected for using the Bonferroni-Holm procedure. Effect size metrics are reported for ANOVA (partial eta squared: η_p_^2^) and EMM (Cohen’s d, *d*) alongside the associated two-tailed 95% confidence intervals (effect size calculations are described in the Supplementary Methods). Variables with skewness of ≥ ±1 or kurtosis ≥ ±5 were log-transformed, and underwent confidence interval bootstrapping for EMMs tests. In addition to normality checks, models were verified for covariate independence and homogeneity of regression slopes by assessing covariate interactions across factors. Homogeneity of demographic covariates across groups was assessed using chi-squared (X^2^) independence tests (binary or categorical data) or independent two-tailed t-tests (discrete or continuous data). Participant group estimation results were analysed via X^2^ independence tests. Inferential analyses were carried out at an alpha level of 0.05. A sample size calculation determined a required sample size of *N* = 52 was needed to undertake two-tailed between-groups inferential statistics (Power [1 - β error probability]: 80%).

### MRI data acquisition, preprocessing, and analysis

Multi-modality MRI data was acquired using a 3-Tesla Prisma Siemens scanner utilising a 32-channel head matrix coil at the Oxford Centre for Human Brain Activity (University of Oxford). The scan started with T1-weighted anatomical image acquisition across 192 slices (TR = 1900ms, TE = 3.97 ms, field of view = 192 mm, flip angle = 8°, voxel dimensions = 1mm^3^, acquisition time = 5 mins, 31 sec). Functional image sequences were T2-weighted echoplanar images and were parameterised to optimise for image fidelity in regions of interest (ROIs). The first task, the novel memory encoding task, was acquired over one run (TR = 800 ms, TE = 30 ms, flip angle = 52°, slice thickness = 2 mm, multiband accelerator factor 6, resolution = 2.4mm^3^ isotropic voxel size, acquisition time = 6 mins, 48 sec). The second task, the complex working memory task, was also acquired over one run (TR = 1500 ms, TE = 25 ms, flip angle = 70°, slice thickness = 2 mm, multiband accelerator factor 3, PAT GRAPPA acceleration factor 2, resolution = 2mm^3^ isotropic voxel size, acquisition time = 15 mins, 9 sec). To assist in distortion correction of functional images, fieldmaps were acquired before task runs (TE1 4.92 ms, TE2 7.38 ms; TR = 590 ms, flip angle = 46°).

Structural and functional data were converted to BIDS specification using *HeuDiConv* (v 1.0.1)(https://github.com/nipy/heudiconv) and converted to NIfTI-1 using *dcm2niix* (v1.0.20220720)^150^. Image quality for structural and functional data was assessed using *MRIQC* (v23.1.0)^151^. Structural images were defaced using *pydeface* (v2.0.2) (https://github.com/poldracklab/pydeface), bias-field corrected using *FSL* (version 6.00)^152^, deep-learning based skull-stripping via *synthstrip* (v1.3)^153^. All functional images were registered to T1-weighted images using FSL’s *FLIRT/FNIRT*^154^ and normalised to Montreal Neurological Institute (MNI) space.

Functional resting state data underwent first-level preprocessing via FSL’s *MELODIC* tool: high pass temporal filtering occurred at 99 s, motion correction was undertaken via MCFLIRT^155^, field-map correction, and automatic dimensionality estimation of components. These pre-processed data were further denoised with a supervised learning approach via ICA-FIX^156,157^. To account for the specific acquisition sequence used, the ICA-FIX model was self-trained on signal/noise discrimination via hand-labelling of 16 participants from this dataset; leave-one-out (LOO) cross-validation produced a 3 × *TPR* + *TNR* of 99.9% at a threshold of 30, which was the threshold used for denoising the full dataset. Following denoising, the data were smoothed using a Gaussian kernel of 2.5 mm and normalised to MNI space.

To investigate how increased histamine influences offline network dynamics, a priori ROIs for network nodes were defined. ROIs were restricted to regions with strong evidence for both (*i*) involvement in memory processing (and thus also used in the memory encoding task), and (*ii*) sensitivity to histamine H_3_R modulation, based on prior animal and human work^9,38,39,49,63–69^. This approach reduced dimensionality and minimised the risk of spurious network effects. Additional temporal-limbic regions were excluded where no evidence for H_3_R involvement was available. An ROI of the bilateral hippocampus was derived from the Jülich histological probabilistic atlas^158^, containing a combined bilateral mask of the dentate gyrus and cornu ammonis (CA) (set to a > 0.50 probability threshold). An a priori ROI of the mammillary zone, which contains the TMN (origin of histamine neurons) as indicated in past work^71,72^, was derived from the Jülich atlas ( > 0.25 probability threshold)^159^. Additionally, an a priori ROI of the basal forebrain was also derived from the Jülich atlas^158,160^. Further ROIs of the bilateral entorhinal cortex (set to a > 0.50 probability threshold), also derived from the Jülich atlas^158,160^, and bilateral perirhinal cortex masks were created using 5 mm radius spheres with reference to previous work on this region (MNI coordinates: left mask centre X,Y,Z [-33, -4, -32]; left mask centre [33, -7, -29])^161^. To correct for multiple comparisons, a priori ROIs were bilateralised/contained all divisions. During the post-learning resting stage, time series signals were extracted from these ROIs; extracted signals were multiplicatively weighted using a rank-based inverse normalised PET image of regional H_3_R density from neuromaps^70^. These images were originally supplied by Gallezot et al^31^. from a sample of participants with a mean age of 35 ± 10 (*N* = 9; all male), within 6 years of the mean age range within the current study.

Covariance network matrices of H_3_R-weighted regions of interest (ROIs, or nodes) were generated using *fslnets* (http://fsl.fmrib.ox.ac.uk/fsl/fslwiki/FSLNets). Multivariate analysis of these covariance matrices was performed using linear discriminant analysis, applied to regularised partial correlation matrices derived from all participants’ data (from *scikit-learn*^162^). This machine learning algorithm employs LOO cross-validation to train and test classifiers, enabling the discrimination of network matrices between different allocation groups. These analyses were also performed on the non-weighted dataset and are reported in Supplementary Note 1.

Task-related functional data were preprocessed and analysed (first-level) using FSL’s *FEAT* tool: motion correction was undertaken using *MCFLIRT*^155^, with spatial smoothing set to a Gaussian kernel of 5 mm (full-width-half maximum), high-pass temporal filtering (fitting a straight line using Gaussian-weighted least squares; memory encoding task σ = 45.0 s; working memory task σ = 49.5 s), field-map correction, normalisation of the 4D dataset grand-mean intensity using a single multiplicative factor, and distortion correction via B0 unwarping using phase and magnitude images. For task-related functional images, motion outlier confound matrices were created using *FSL*. During registration, functional images were aligned to a single-band functional reference image with increased contrast acquired before the task acquisition sequence. Functional image datasets with high motion (maximum absolute displacement of ≥ 1.5 mm) or technical issues during the scan (e.g., scanner failure during time-dependent sequence) were excluded from the dataset (*N* = 6 for all functional sequences). H_3_R-weighting was not applied to task-based fMRI analyses, as the permutation-based GLM pipeline (FSL’s randomise) does not permit integration of external PET-derived weights. We therefore prioritised analysis of unweighted task-related neural signal, before examining the histaminergic specificity of associations via post hoc application of receptor-weighting to extracted parameter estimates (see Supplementary Methods).

For task-related functional data, higher level analysis was undertaken using non-parametric permutation inferential analysis via FSL’s randomise^163^, utilising the threshold-free cluster enhancement (TFCE) method set to *p* < 0.05. Significant clusters identified through TFCE were summarized using descriptive statistics of the peak voxel within each cluster (i.e., location, t-statistic and p-value). Motion outlier confound matrices and motion parameters were included in higher-level analysis. The main effect of task was analysed at the whole brain level, while the group-level analyses (pitolisant vs. placebo) were restricted to a priori ROIs which overlap with histological maps of histaminergic innervation (small volume correction [SVC])^9,39,63,64,164^. Follow-up exploratory analyses for both tasks undertaken using a whole-brain mask. For the novel memory encoding task, neural activity during new learning was contrasted with previously learned information (novel > familiar contrast). In addition, BOLD signal from each rest period (12 s) was decomposed using finite impulse response across 15 temporal bins (one bin per 0.8-s TR). These bins were concatenated within a linear model which captured signal decay which contrasted rests following novel > familiar encoding (${t}_{1}$ novel [EV -7.5] → ${t}_{15}$ novel [EV + 6.5]; ${t}_{1}$ familiar [EV + 6.5] → ${t}_{15}$ familiar [EV -7.5]). For the complex working memory task, increased activity as a function of increased task complexity/load was linearly modelled (0-back [EV -1.5], 1-back [EV -0.5], 2-back [EV + 0.5], and 3-back [EV + 1.5]). Alongside this, contrasts across each level of task complexity were undertaken: 1 > 0-back, 2 > 0-back, and 3 > 0-back. During the memory encoding and complex working memory tasks, the same a priori ROIs from the network analysis were reapplied: bilateral hippocampus, basal forebrain, mammillary zone, entorhinal and perirhinal cortices. Each ROI was bilateralised/contained all divisions. For the complex working memory task, ROIs were created over the bilateral medial DLPFC (BA46 and dorsal transition area BA46/9) and bilateral rostral DLPFC (BA9), per previous subdivisions of this region and task relevance^165,166^, using cortical maps from the Human Connectome Project atlas^167^. Given functional differences across hemispheric divisions of the DLPFC in working memory function^165,168–170^, each division was treated as distinct. In addition, given the role of the dopaminergic substantia nigra in *n*-back performance, alongside its histaminergic innervation^148,164^, an ROI mask derived from previous work was selected (set to a > 0.25 probability threshold)^171,172^. All ROIs were resampled to 2mm^3^ MNI space and were previously investigated at 3 T fMRI^148,161,165,172–176^. Significant clusters for each ROI are reported alongside their corresponding peak MNI coordinates. Parameter estimates from significant clusters were used to assess their relationship with experimental variables such as connectivity values (see Supplementary Methods for details). The voxel-wise statistical maps resulting from permutation testing were processed and visualised using *Nilearn* (v 0.10.4) (https://github.com/nilearn/nilearn)^177^ and *Nibabel* (v 5.2.1) (https://github.com/nipy/nibabel)^178^.

Arterial Spin Labelling (ASL) was undertaken to explore the potential influence of pitolisant on cerebral blood flow. Details on the ASL sequence parameterisation, preprocessing and analysis are provided in the Supplementary Methods.

### Reporting summary

Further information on research design is available in the Nature Portfolio Reporting Summary linked to this article.

## Supplementary information

Supplementary Information

Reporting Summary

Transparent Peer Review file

## Data Availability

The raw and modelled data generated for this study have been deposited on Zenodo^179^ and Github: https://github.com/mjcolwell/Histamine_Learning_Data_and_Code.

## Code availability

The code used to undertake preprocessing, network analysis, computational modelling and inferential modelling are available on Zenodo^179^ and Github: https://github.com/mjcolwell/Histamine_Learning_Data_and_Code.
