# Supplementary material for: Histamine shapes the neurocomputational dynamics of human learning
Source: Nat Commun. 2026 Jun 2;17:7124. doi: 10.1038/s41467-026-73865-9 (PMC13396361; doi:10.1038/s41467-026-73865-9)
Supplement: Supplementary file 1 — Supplementary Information [file 41467_2026_73865_MOESM1_ESM.pdf]

Supplementary Information

Histamine shapes the neurocomputational dynamics of human learning

Colwell et al. 2026. Published in *Nature Communications*.

Supplementary Methods

Questionnaire Battery, Procedure, and Analysis

A battery of self-report questionnaires was administered at baseline (i.e., the start of the study visit) and again at the end of the visit to assess self-rated state anxiety, anhedonia, affect, and side effects. These included the Spielberger State Anxiety Inventory (STAI-S) [1], the Dimensional Anhedonia Rating Scale (DARS) [2], the Positive and Negative Affect Schedule (PANAS) [3], a Visual Analogue Scale (VAS), and a side effects profile questionnaire. As side effects were recorded only during the study visit, items relating to insomnia and sexual side effects were excluded from analysis.

In addition to these established measures, a novel instrument – the State-Subjective Cognition Scale (SSCS) – was employed to assess subjective cognitive states. The SSCS is a validated state-based measure developed to address limitations of existing retrospective measures of subjective cognition (van Uum, Colwell, & Murphy, under review). It comprises thirteen items rated on 100-point visual analogue scales anchored at opposing descriptors, grouped into five psychometrically validated subscales (memory, selective attention, executive function, motivation, and mood). Subscale structure was derived using exploratory factor analysis and confirmed in an independent sample using confirmatory factor analysis, with all subscales showing good internal consistency and convergent validity with established subjective cognition and affect measures. Negatively valenced items were reverse-coded such that higher scores uniformly reflected better subjective cognitive functioning and affective state. The SSCS was administered at four time points during the study visit: at baseline (T_1), prior to MRI scanning (2.5 hours post-dose; T_2), immediately following MRI scanning (4 hours post-dose; T_3), and at the conclusion of the visit (T_4). An exploratory analysis examined associations between post-scan self-rated cognition (SSCS; T_3) and objective cognitive outcomes (see Supplementary Table 13). SSCS scores at T_3 were used as this assessment was temporally closest to task performance. Correlations were tested for the SSCS composite score and its five subdomains, with significance values adjusted using false discovery rate correction.

For the main questionnaire battery (excluding the SSCS), post-dose questionnaire scores were analysed using baseline-adjusted mixed-effects ANCOVA models. In these models, post-intervention scores served as the outcome variable, with baseline (pre-intervention) scores included as a covariate, and participant was included as a random effect. Given the added temporal dimension of the SSCS ($T_{1\text{-}4}$), these data were analysed using linear mixed-effects models with restricted maximum likelihood estimation, incorporating time as a fixed effect. As with task data, planned pairwise comparisons for questionnaire data were conducted using estimated marginal means

with two-tailed tests, and multiple comparisons were corrected using the Bonferroni-Holm procedure. All inferential tests were evaluated at an alpha level of 0.05.

Memory Recognition Task Analysis – Drift Diffusion Modelling

The memory recognition task, conducted during the final phase of the multistage fMRI memory paradigm, assessed participants' memory for previously encoded visual items among novel distractors. Task behaviour was fit to a computational model of evidence accumulation, a Bayesian drift diffusion model (DDM). The DDM provides access to latent cognitive processes which occur during evidence accumulation, providing potential explanations for patterns observed within task behaviour [4]. Our DDM approach utilised the publicly available *HDDM* [5] package available in Python, which allows undertaking Bayesian Monte Carlo Markov Chain (MCMC) sampling (via *PyMC* [6]) of all model parameters per participant. Each model was calculated with four Markov chains with 4,000 samples per chain, of which the first 2,000 were discarded as burn-in. Models were assessed for convergence via *ArviZ* [7] using the Gelman-Rubin diagnostic (rank-normalized, split-$\hat{R}$); the threshold for acceptable convergence was set to $\hat{R} \leq 1.01$. Model selection was guided by the deviance information criterion (DIC), which balances model fit and complexity [8]. The initial candidate model was the simplest possible DDM, consisting of three parameters: drift rate parameter (v), decision policy (a), trial variability, and non-decision time (T_{er}). Model selection occurred through iteratively increasing complexity through a stepwise approach (Supplementary Table 5). Allowing parameters to vary according to stimulus type ('previously encoded' or 'distractor'), and the inclusion of inter-trial variability parameters resulted in better model fit. The stimulus-dependent estimation of parameters aligns with previous implementations of the DDM for recognition memory [9]. The winning model consisted of four stimulus-dependent parameters, v_s, a_s, $T_{er,s}$ and z_s, with two fixed inter-trial variability parameters: st and sz. This model can be conceptualised as following:

$$\mathrm{RT}(x) \approx \frac{{a_s}^{eff}(z'_s)}{v_s} + T_{er,s} + \mathcal{N}(0, st^2)$$

where the trial-wise starting point z'_s varies according to:

$$z'_s \sim \mathrm{Uniform}\left(z_s - \frac{sz}{2}, z_s + \frac{sz}{2}\right)$$

Where the differential varies according to if the lower boundary is hit ${a_s}^{eff} = a_s \cdot z'_s$, or if the upper boundary is hit $a_s \cdot (1 - z'_s)$. v_s was sampled in unconstrained Euclidean space, while a_s, z_s, sz and st were sampled in space and transformed to positive reals. $T_{er,s}$ was sampled within a space bounded by the observed RT range.). Model parameters were estimated for individual participants without access to group-level posteriors to allow frequentist statistical analyses. Posterior predictive checks

indicated a close match between synthetic data retrieved from the model fitting process and observed behaviour (Supplementary Fig. 5). Parameter recovery for stimulus-dependent and fixed parameters was performed using *pyMC* [6] MCMC, using true parameter estimates across 200,000 trials. All parameter estimates were checked for recoverability (Supplementary Fig. 6), while the winning model demonstrated adequate convergence during fitting ($\hat{R} \leq 1.01$). Before inferential analyses, decision policy a_s was log transformed, z_s was inverse logit transformed, while T_{er} was arcsine transformed. All parameters were analysed using mixed-effects ANOVA and EMM modelling approaches.

N-Back outcome analyses and drift diffusion modelling

Due to excess skewness (-1.67 [threshold ≥ ±1]) and kurtosis values (6.25 [threshold ≥ ±5]), raw accuracy (%) values on the *n*-back were log-transformed, and EMM tests included bootstrapped confidence intervals. Raw accuracy (%) values are retained within Fig. 4D (main text) for interpretability.

A Bayesian drift diffusion model (DDM) was validated for the verbal *n*-back task as part of the present study. As with the memory recognition task, the DDM was fit via HDDM [5] with each model being calculation across four Markov chains with 4,000 samples per chain, of which the first 2,000 were discarded as burn-in. In addition, model convergence was assessed using a split-$\hat{R}$ criterion of ≤ 1.01. The initial candidate model was selected based on previous work with the DDM which included a drift rate parameter (v), an initial starting bias parameter (z), decision policy (a), trial variability, and non-decision time (T_{er}) [10]. Due to the high trial type imbalance (20-30% cued targets per block), we considered models with separate drift rates ($+v/-v$). In our model comparison, the best-fitting model included split drift rate but excluded initial choice bias, as it yielded the lowest Deviance Information Criterion (Supplementary Table 8).

Drift criterion for split drift rate was not included as there was no theoretical basis for its influence in this paradigm [10]. While more complicated models were considered, high parameterisation has been reported to reduce detection of group-level effects from DDM parameters [11]. The winning candidate model ($+v/-v,\ a,\ T_{er},\ st,\ sv$) can be conceptualised as following:

$$\mathrm{RT}(x) \approx \frac{a}{v + \mathcal{N}(0,\ sv^2)} + T_{er} + \mathcal{N}(0, st^2)$$

As with the memory recognition task, all models parameters ($v,\ a,\ st$ and sv) were sampled in Euclidean space, except T_{er} which was sampled in a space bounded within the observed RT values. Model parameters were estimated at the individual level without incorporating group-level posteriors, enabling subsequent frequentist

statistical analyses. Posterior predictive checks showed a close match between observed task and synthetic data derived from the model fitting process for participant data (Main text, Fig. 4C). Parameter recovery was undertaken using synthetic data simulated via *pyMC* MCMC using true parameter estimates for each model parameter across 200,000 trials. These simulated data were fitted using the winning DDM, and recovered parameter values were compared against their true values. All model parameters were deemed recoverable from the fitting process (Supplementary Fig. 8A). The winning model converged well during the fitting process ($\hat{R} \leq 1.01$). For inferential analyses, decision policy a was log transformed, while T_{er} was arcsine transformed. All parameters were analysed using mixed-effects ANOVA and estimated marginal means (EMM) modelling approaches.

Using simulated task data from the posterior predictive checks, we performed post-hoc analyses to assess if high and low T_{er} (split using within-subjects median-split) influence posterior accuracy. We ran a mixed-effects ANOVA model with T_{er} bins as a between-subjects factor and posterior accuracy as the outcome variable and estimated marginal means planned comparisons.

Probabilistic Instrumental Learning Task Analysis and Reinforcement Learning Modelling

For the analysis of behavioural performance, the final half of trials (10 per condition) were analysed, as this reflects the plateau phase of learning performance [12,13]. One participant was excluded due to data quality issues. Exploratory analyses including all trials is reported in Supplementary Note 9, alongside analyses comparing overall monetary gain across allocation groups.

Behavioural data were fit using a computational reinforcement learning model (Q-learning), consistent with prior implementations [12,14,15]. Model selection details are provided in [14], with parameter recovery checks reported in [12]. Posterior predictive checks were conducted by comparing observed temporal learning patterns with those simulated from the fitted models (see Supplementary Fig. 14D; Main text, Fig. 5B). The value of a selected stimulus was updated on each trial, with the value of the unselected stimulus updated reciprocally. The learning algorithm is described in the following equation:

$$Q_{t+1(s)} = Q_{t(s)} + \alpha_j \left(R_t - Q_{t(s')}\right)$$

The Q value, or belief expectation, $Q_{t(s)}$, describes the value associated with a symbol (s) at a given trial (t). R_t refers to the type of outcome observed, which can be positive (win during high-probability win [1], or avoiding loss during high-probability loss [0]) or negative (no change during high-probability win [0], or loss during high-probability loss [-1]). The learning rate parameter α_j , which determines the slope of the learning curve, is set for each trial type j (win/loss trials) and was sampled in logit space. In line with previous studies [14], $Q_{0(s)}$ was initialised at 0.5. Each trial, the unselected option, described as $Q_{t(s')}$, was updated reciprocally based on the counterfactual outcome

from the selected symbol. The Q value assigned to the first two paired stimuli were used to provide an estimate of choice probability through a softmax function:

$$P_{t(s)} = \frac{1}{1 + exp^{-\beta_j\left(Q_{t(s)} - Q_{t(s')}\right)}}$$

The probability ($P_{t(s)}$) of choosing a given the shape s is controlled by the inverse decision temperature parameter β (sampled in log space). As with the learning rate parameter, β is set for each trial type j. Modelling win and loss trials separately was required to observe valence-specific effects and is in line with previous implementations of the model [12]. Each parameter was estimated within discrete space (110 × 100 grid) by determining the posterior probability of each trial type, then by calculating the expected value for each marginal likelihood parameter. The α parameter was log transformed before inferential analyses using mixed-effects ANOVA and planned comparisons EMM tests. Given the high covariance between model parameters due to within-subjects trade-off, mixed-effects ANOVA models were adjusted to include the reciprocal parameters as random effect terms: `(parameter (α/β) ~ group * condition (loss/win) + (1 + parameter (α/β) || Participant))`. Independent random intercepts were specified to account for condition-level parameter trade-offs, as computational models were fit separately for each condition within participants.

Beyond the computational model reported, alternative models were considered; these include models where outcome sensitivity and value decay are parameterised. However, these parameters are mathematically redundant [16], therefore there is no theoretical prior to justify their inclusion. Given the lack of previous work on histamine and reinforcement learning, it was decided to use the legacy Q-learning parameter β [15].

Global and Regional Blood Perfusion Analyses

Following rsfMRI and task-based fMRI sequences, an arterial spin labelling sequence occurred to estimate global and regional blood flow across allocation groups. Participant structural data was preprocessed used FSL's anatomical processing script (*FSL_Anat*; https://fsl.fmrib.ox.ac.uk/fsl/fslwiki/fsl_anat) which entailed bias-field correction, brain extraction and registration to standard MNI152 space via FMRIB's *FNIRT* tool). ASL perfusion maps were distortion-corrected and motion-corrected within units of mL/100mg/min via FSL's *BASIL* (Bayesian Inference for Arterial Spin Labelling tool, https://fsl.fmrib.ox.ac.uk/fsl/fslwiki/BASIL) [17,18]. BASIL performed voxelwise calibration, label-control subtraction, and inferrential estimation of voxel-wise perfusion to generate absolute perfusion maps, controlling for the partial volume effects observed at the participant level. These perfusion images were then registered to standard space using the non-linear warp generated via *FSL_Anat* via *FNIRT*. The transformed and calibrated perfusion maps were analysed using global CSF values

output by BASIL for grey and white matter and analysed via between-groups ANOVA modelling. For regional CSF analysis, voxelwise glm permutation testing (5000 permutations, with TFCE and FWE correction for cluster significance) was undertaken within whole-brain and task-network maps via FSL's *randomise*. For inferrential analyses of calibrated perfusion maps, there were missing data for one participant (due to scanner sequence issues) and exclusions due to technical issues during the scan (*N*=5). Global estimates of blood flow analyses included grey and white matter masks. Regional estimates of blood flow were undertaken within a mask of *a priori* region of interests for the fMRI sequences (memory consolidation/encoding and complex working memory).

fMRI Resting State and Task-Based Correlational Analyses

Linear regression analyses (including two-tailed Pearson's product-moment tests) were conducted to examine the relationship between group-level effects observed across outcome variables (including fMRI TFCE-corrected cluster β values and computational parameters) within the primary analysis. The relationship between Mammillary zone–hippocampal connectivity (predictor) and both hippocampal encoding cluster activity (outcome) and entorhinal memory trace cluster (outcome) was assessed (see main text, Fig. 2C – G, I). In addition, the relationship between trace decay (predictor) and computational decision parameters during recall (drift rate v_s and decision policy a_s) was assessed. For the *n*-back task, the relationship between drift rate v (outcome) was assessed with activity within the left DLPFC cluster as task complexity increased (predictor). While a significant group effect was observed for non-decision time T_{er} (see main Results), there was no theoretical basis for its examining its relationship with DLPFC activity given activity within this region has been causally linked to v across multiple studies, but not T_{er} [19,20]. Where appropriate, correlational analyses were corrected for multiple comparisons using Holm–Bonferroni adjustment. For each linear regression analysis, follow-up models were fitted separately within each allocation group using ordinary least squares. This allowed estimation of the predictor–outcome relationship independently within groups, without assuming a shared effect across conditions. Regression coefficients from these within-group models were then aggregated to characterize the posterior distribution of effects across groups.

For all correlational analyses involving fMRI signal, the signal was weighted based on the spatial density of regional H_3R (rank-based inverse normalized). A comparison between weighted and non-weighted group-level effect sizes is reported in Supplementary Fig. 15. In addition, a comparison between weighted and non-weighted correlational analyses is reported in Supplementary Table 9.

Effect Size Calculation

Effect sizes were derived for both mixed-effects ANOVA models and estimated marginal means (EMM) comparisons. For ANOVA models, partial eta squared (ηp^2) was computed using the *eta_squared* function from the *effectsize* R package, applied to the fitted mixed-effects model. For EMM pairwise comparisons, Cohen's *d* was estimated using the *eff_size* function from the *emmeans* R package, based on follow-up outcome values. For permutation tests reported in the main text, effect sizes were computed from the observed parameter estimates corresponding to the tested contrasts.

Behavioural Data Software – Collection and Analysis

For data collection, the following software packages were used: *PsychoPy* 2021.1.4; *MATLAB* 2021a; *Qualtrics Surveys*; *Anaconda* 2021.4; *Presentation* (Neurobs) 23.0; *Psychtoolbox* v3. For data analysis, the following software packages were used: *Docker* 4.22.0; *MATLAB* (R2022a); *Python* (version 3.8.8); R (4.3.1). The following R packages were utilised: *moments* (0.14.1); *effectsize* (0.8.6); *ggbeeswarm* (0.7.2); *gtools* (3.9.4); *readxl* (1.4.2); lme4 (1.1-33); *sdamr* (0.2.0); *plotrix* (3.8-2); *ggridges* (0.5.4); *tidyverse* (2.0.0); *knitr* (1.42); car (3.1-2); *stringr* (1.5.0); *ggplot2* (3.4.2); *ggsignif* (0.6.4); *ggpubr* (0.6.0); *viridis* (0.6.4); *ggdist* (3.3.0); *ggstance* (0.3.6); *RColorBrewer* (1.1-3); *ggrepel* (0.9.3); *emmeans* (1.8.5); *ggpp* (0.5.4); *lmerTest* (3.1-3); *cowplot* (1.1.1); *openxlsx* (4.2.5.2); *ez* (4.4-0); *gghalves* (0.1.4); *ggsignif* (0.6.4); *data.table* (1.14.8); *ggplot2* (3.4.2); *psycho* (0.6.1); *gtools* (3.9.4); *ggExtra* (0.10.1); . All required Python dependencies are included within the Docker image: hcp4715/hddm:0.8. No additional MATLAB packages required.

Supplementary Note 1

Following iterative encoding of visual stimuli in phase one of the multi-stage fMRI task, a short test was undertaken to ensure a high accuracy criterion was achieved (≥85% accuracy). Participants discriminated between encoded items and novel distractors above criterion (mean = 98.98% ± 2.66). There was no significant difference in accuracy across allocation groups (Main effect of group [mixed effects ANOVA]: F[1,47] = 0.003, *p* = 0.957).

Supplementary Note 2

A network analysis without time series weighting using histamine 3 (H_3R) receptor maps was undertaken. Binary classification machine learning was able to differentiate between pitolisant and placebo with the same level of accuracy (88.46%). Connectivity between the mammillary zone and hippocampus was higher in individuals who received the drug vs. placebo (*t*[51] = 2.79, *p* = 0.035, FWE-corrected, Cohen's *d* = 0.77).

Supplementary Note 3

During the formation of novel memories (novel > familiar contrast), there was increased activity across multiple brain regions in the pitolisant group, identified via *a priori* SVC permutation testing. These areas included the bilateral hippocampus (left hippocampus cluster: peak MNI coordinates = X,Y,Z [-22, -14, -26]; cluster size (voxels) = 89; *t*-max (51) = 4.651,*p* = 0.0054, TFCE-corrected; right hippocampus cluster 1: peak MNI coordinates = [22, -12, -26]; cluster size = 36; *t*-max (51) = 4.037, *p* = 0.0162, TFCE-corrected; right hippocampus cluster 2: peak MNI coordinates = [36, -26, -18]; cluster size = 6; *t*-max (51) = 3.724, *p* = 0.0396), basal forebrain (peak MNI coordinates = [6, 2, -16]; cluster size = 5; t-max (51) = 3.843, *p* = 0.0330, TFCE-corrected), bilateral perirhinal cortex (left perirhinal cortex cluster: peak MNI coordinates = [-34, -6, -34]; cluster size = 10; t-max (51) = 3.462, *p* = 0.0178, TFCE-corrected; right perirhinal cortex cluster 1:

peak MNI coordinates = [36, 8, -34]; cluster size = 5; *t*-max (51) = 3.306, p = 0.0244, TFCE-corrected; right perirhinal cortex cluster 2: peak MNI coordinates = [38, -6, -28]; cluster size = 1; *t*-max (51) = 2.777, p = 0.0470, TFCE-corrected), and bilateral entorhinal cortex (right entorhinal cortex cluster 1: peak MNI coordinates = [22, -16, -28], cluster size = 53; *t*-max (51) = 4.256, p = 0.0016, TFCE-corrected; right entorhinal cortex cluster 2: peak MNI coordinates = [14, -6, -20], cluster size = 1; *t*-max (51) = 2.698, p = 0.0464, TFCE-corrected; left entorhinal cortex cluster: peak MNI coordinates = [-22, -16, -28], cluster size = 7; *t*-max (51) = 3.978, p = 0.0194, TFCE-corrected).

Supplementary Note 4

An analysis of labelling discrimination ('animal' or 'landscape') during the fMRI encoding task was undertaken to ensure visual stimuli were properly discriminated within the scanner. There was no significant main effect of allocation on labelling discrimination accuracy (F[1,50] = 1.72, p = 0.195); in addition, there was no significant main effect of group on labelling speed (F[1,50] = 0.06, p = 0.803) (Supplementary Fig. 2).

Supplementary Note 5

We examined whether the lateralisation of entorhinal trace persistence (main text, Fig. 2E – G) was mirrored by lateralised activity during encoding in significant clusters. There was a main effect of the left hippocampus (β = 0.43, p = 0.0132, η_p^2 = 0.12 [0.01, 1.00]; r = 0.34) and left entorhinal cortex (β = 0.49, p = 0.00887; η_p^2 = 0.13 [0.02, 1.00]; r = 0.37) on signal trace persistence. However, there was no statistically significant main effect of the right hippocampus (β = 0.17, p = 0.430) and right entorhinal cortex (β = 0.23, p = 0.266).

Supplementary Note 6

Estimated marginal means (EMMs) for accuracy, drift rate, and non-decision time across n-back levels are reported here (as supplement to Fig. 4D, main text). Participants receiving pitolisant showed generalised increases in accuracy (0-back EMM (± SE) = 0.04 ± 0.03, p = 0.115; 1-back EMM = 0.08 ± 0.03, p = 0.0012, d = 0.87 [0.30, 1.44]; 2-back EMM = 0.12 ± 0.03, p = 0.0004, d = 1.36 [0.84, 1.94]; 3-back EMM = 0.10 ± 0.03, p = 0.0008, d = 1.13 [0.60, 1.66]) and drift rate (0-back EMM = 0.42 ± 0.26, p = 1.090413e-01; 1-back EMM = 0.63 ± 0.26, p = 1.626182e-02, d = 0.89 [0.16, 1.62]; 2-back EMM = 0.48 ± 0.26, p = 6.321225e-02; 3-back EMM = 0.19 ± 0.26, p = 4.521472e-01). As task complexity increased, non-decision time elevated in the pitolisant group (0-back EMM = 0.04 ± 0.03, p = 1.930955e-01; 1-back = 0.066 ± 0.032, p = 4.277534e-02, d = 0.78 [0.02, 1.54]; 2-back = 0.127 ± 0.032, p = 1.541361e-04, d = 1.49 [0.72, 2.25]; 3-back = 0.11 ± 0.032, p = 8.740529e-04, d = 1.30 [0.53, 2.06]). Splitting trials by high vs. low T_{er} revealed decreased posterior accuracy under low load but improved accuracy under high load (0-back EMM = -0.03 ± 0.03, p = 0.926; 1-back = -0.63 ± 1.75, p = 0.004, d = -0.63 [-1.38, -0.19]; 2-back = 0.02 ± 0.004, p = 0.032, d = 0.40 [0.04, 1.37]; 3-back = 0.03 ± 0.02, p = 0.004, d = 0.69 [0.27, 1.52]).

Supplementary Note 7

We ran confirmatory analyses on the relationship between posterior *n*-back accuracy (mean %) and continuous T_{er}, using the following mixed effects model: `(`T_{er} `~ accuracy*condition [reference = 0-back] + (1 | Participant))`. There was a significant interaction between accuracy and medium task difficulty (2-back; β = 1.25 [0.13, 2.38], $t(176) = 2.20$, $p = 0.0293$) and the hardest task difficulty (3-back; β = 1.22 [0.11, 2.33], $t(173) = 2.17$, $p = 0.0317$), while no further significant interactions were observed (1-back; β = 0.90 [-0.20, 2.01], $t(167) = 1.61$, $p = 0.108$. In addition, the inclusion of group allocation as a covariate within the model yielded similar interaction effects (1-back: β = 0.98 [-0.11, 2.08], $t(169) = 1.77$, $p = 0.0785$; 2-back: β = 1.36 [0.24, 2.47], $t(178) = 2.40$, $p = 0.0175$; 3-back: β = 1.32 [0.22, 2.42], $t(175) = 2.36$, $p = 0.0194$).

In addition to the median-split T_{er} analysis on posterior accuracy (see Results, main text), we assessed the potential influence of group allocation by including it as a covariate within the median-split T_{er} analysis. Here, the significant T_{er} × task complexity interaction persisted for both the observed (F[3,149] = 2.86, $p = 0.0391$, $\eta_p^2 = 0.05$ [0.00, 1.00]) and posterior data models (F[3,149] = 3.05, $p = 0.0305$, $\eta_p^2 = 0.04$ [0.00, 1.00]).

Supplementary Note 8

An exploratory analysis across all task trials (*n*=60) on the Probabilistic Instrumental Learning Task was undertaken. Consistent with the primary analyses, those who received pitolisant showed higher optimal choices (F[1,55] = 7.03, $p = 0.0104$, $\eta_p^2 = 0.11$ [0.01, 0.28]; win trials EMM = 0.13 ± 0.06, $p = 0.0285$, $d = 0.75$ [0.07, 1.43]; loss trials EMM = 0.13 ± 0.06, $p = 0.0309$, $d = 0.74$ [0.06, 1.42]), while there was no significant difference in time to choice (F[1,55] = 3.16, $p = 0.0811$).

To explore whether the significant group × task condition interaction on learning rate (log α) was attributable to artefacts of early stochastic responding, we repeated the model after excluding the first 10 trials of each condition. The interaction remained significant (main effect of group: F[1,54] = 9.36, $p = 0.0035$, $\eta_p^2 = 0.15$ [0.03, 1.00]; group × task condition interaction: F[1,49] = 4.41, $p = 0.041$, $\eta_p^2 = 0.08$ [0.00, 1.00]; win trials EMM = 0.27 ± 0.29, $p = 0.35$; loss trials EMM = 1.21 ± 0.38, $p = 0.0023$, $d = 1.21$ [0.45, 1.97]). Moreover, there was no significant main effect of group or group × task condition interaction on inverse decision temperature (β; main effect of group: F[1,47] = 0.32, $p = 0.574$; group × task condition interaction: F[1,26] = 1.60, $p = 0.2169$).

An additional analysis on total money earned across allocation groups was conducted. Those who received pitolisant received more money overall (EMM(55) = 1.33 ± 0.53, p = 0.0156, $d = 0.66$ [0.12, 1.21]), with the drug group earning on average £1.33 more than the placebo group.

We investigated the potential relationship between RL computational parameters and drift rate (v) parameters across the *n*-back and recognition memory tasks. There was no

significant main effect of *n*-back drift rate on both inverse decision temperature (win trials β = 0.50, *p* = 0.0647; loss trials β = 0.26, *p* = 0.483) and learning rate (win trials α = -0.02, *p* = 0.635; loss trials α = -0.01, *p* = 0.751). Similarly, there was no significant main effect of memory recognition drift rate (mean) on both inverse decision temperature (win trials β = 1.17, *p* = 0.0887; loss trials β = -0.39, *p* = 0.646) and learning rate (win trials α = -0.18, *p* = 0.165; loss trials α = 0.03, *p* = 0.479).

Supplementary Note 9

We examined potential indirect changes in cerebral blood flow across allocation groups which may have driven changes in BOLD signal across rsfMRI and task-based fMRI. In terms of global blood flow, there was no significant difference within either grey matter (ANOVA main effect of group: F[1,50] = 0.64, *p* = 0.428, two-tailed) or white matter across groups (ANCOVA main effect of group: F[1,50] = 0.88, *p* = 0.353) (Supplementary Fig. 16A – B). Similarly, regional blood flow within a whole-brain mask found no significant differences (pitolisant > placebo, peak t[51] = 4.79, *p* = 0.1781, TFCE-corrected; placebo > pitolisant, peak t[51] = 4.91, *p* = 0.2171, TFCE-corrected). Regional blood flow specifically within *a priori* task networks for memory consolidation/encoding (pitolisant > placebo, peak t[51] = 4.054, *p* = 0.0581, TFCE-corrected; placebo > pitolisant, peak t[51] = 1.809, *p* = 0.921, TFCE-corrected) and complex working memory (pitolisant > placebo, peak t[51] = 3.472, *p* = 0.1712, TFCE-corrected; placebo > pitolisant, peak t[51] = 2.125, *p* = 0.906, TFCE-corrected) were not significantly different.

Supplementary Note 10

There were no significant group differences in baseline self-rated fatigue/drowsiness prior to testing (*t*[55] = 0.76, *p* = 0.76, 95% CI [−0.23, 0.17]) between the pitolisant group (mean ± SD = 0.14 ± 0.36) and the placebo group (mean ± SD = 0.17 ± 0.38). Similarly, there were no significant group differences in baseline self-rated insomnia (*t*[27] = 1.44, *p* = 0.161, 95% CI [−0.03, 0.17]), with low scores observed in both the pitolisant (mean ± SD = 0.07 ± 0.26) and placebo groups (mean ± SD = 0.00 ± 0.00). The reduced degrees of freedom reflect the absence of variability in insomnia scores in the placebo group.

Consistent results were observed on baseline visual analogue scale measures. There was no significant difference on the alert–drowsy item (*t*[54] = 0.84, *p* = 0.40, 95% CI [−5.77, 14.10]) between pitolisant (mean ± SD = 22.86 ± 19.65) and placebo participants (mean ± SD = 18.69 ± 17.67). Likewise, there was no significant group difference on the lethargic–energetic item (*t*[53] = −1.91, *p* = 0.06, 95% CI [−15.97, 0.38]), with scores of 65.96 ± 16.39 in the pitolisant group and 73.76 ± 14.29 in the placebo group. Visual analogue scale items were scored bidirectionally, such that lower values reflected the left-anchored descriptor (*e.g.*, alert or lethargic) and higher values reflected the right-anchored descriptor (*e.g.*, drowsy or energetic).

Supplementary Note 11

The core mechanism of pitolisant is believed to be upregulation of synaptic histamine via autoreceptor blockade [21–23]. However, as with most psychoactive drugs [24], there may be ancillary effects present. Levels of nonhistaminergic monoamines remain unchanged in H_3R−/−mice, while there is evidence of clear increases in synaptic histamine [25–27]. Early work on pitolisant suggests it may selectively increase synaptic dopamine in the prefrontal cortex but not striatum [28]. However, in human dopaminergic neurons, H_3R mRNA is low, and therefore increased synaptic dopamine may be an indirect result of synaptic histamine on co-localised monoaminergic neuron subpopulations in the prefrontal region [22,29,30]; this is consistent with the broader network effects typical of classical monoamines at the synapse [31–33]. Indeed, given the abundance of H_3-heteroreceptors in the region, a lack of change in striatal dopamine after pitolisant supports its preference for H_3-autoreceptors [34]. Further, pitolisant may induce a brief spike (~30 min) in prefrontal acetylcholine levels, however, returning to basal state (1hr post-administration) prior to the present study testing period (3hrs) [28], in addition to very small increases in serotonin (15%) selective to the striatum and not serotonin-rich prefrontal and hypothalamic regions [35,36]. Taken together, the region-selectivity of dopamine and small or brief increases in region-selective acetylcholine and serotonin, in comparison to whole-brain changes in synaptic histamine [28,37], are consistent with the preferential action of pitolisant for histamine autoreceptors. Indeed, relative to H_3R agonists, pitolisant does not show intrinsic activity at one of the longest H_3R isoforms, H_3R-453, which is presumed to function as a heteroreceptor [38,39]; moreover, compared to H_3R-445, inverse agonism (*e.g.,* via pitolisant) leads to larger responses in shorter and more constitutively active isoforms, H_3R-365 and H_3R-373, which are believed to function as autoreceptors [38–40]. While pitolisant shows lower binding affinity at these short isoforms, it still produces robust inverse agonism, consistent with their high constitutive activity; moreover, expression of H_3R isoforms in human tissue remains incompletely resolved, so binding affinity does not directly map onto functional effect size [38]. Nevertheless, further work is required to further identify H_3R isoforms in humans (>20 isoforms identified cross-species) [41], and clarify which are preferentially targeted by pitolisant at therapeutically-relevant doses.

Supplementary Figures

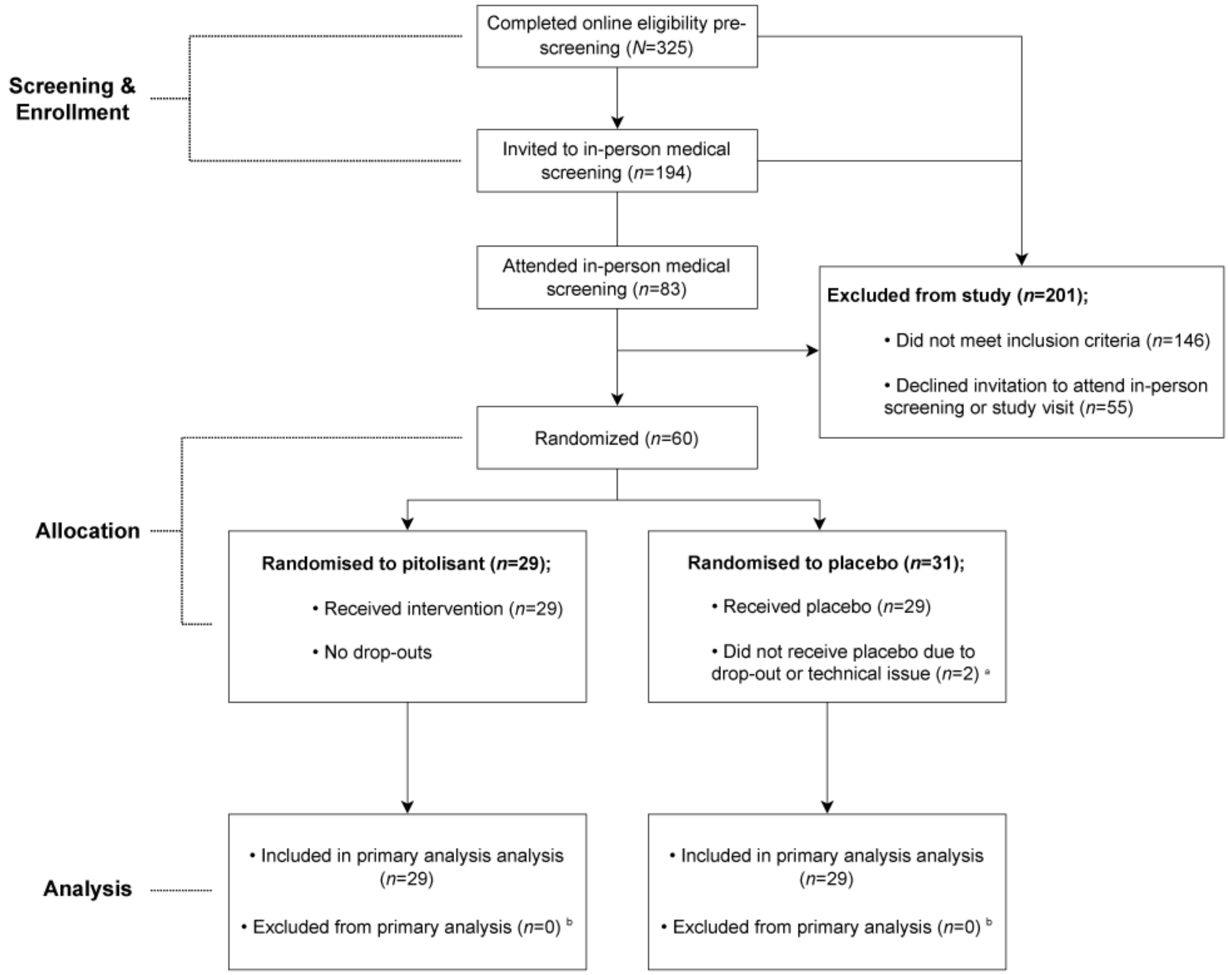

Supplementary Fig. 1. CONSORT flowchart: recruitment and randomisation progression throughout the study. [a] One participant randomised to placebo dropped out of the study prior to administration on the study day; another participant was withdrawn from the study due to technical issues on the study day (*i.e.,* MRI scanner coil failure).[b] Participants were included in all analyses unless there were procedure-specific data quality concerns (*e.g.,* extreme framewise displacement during fMRI tasks).

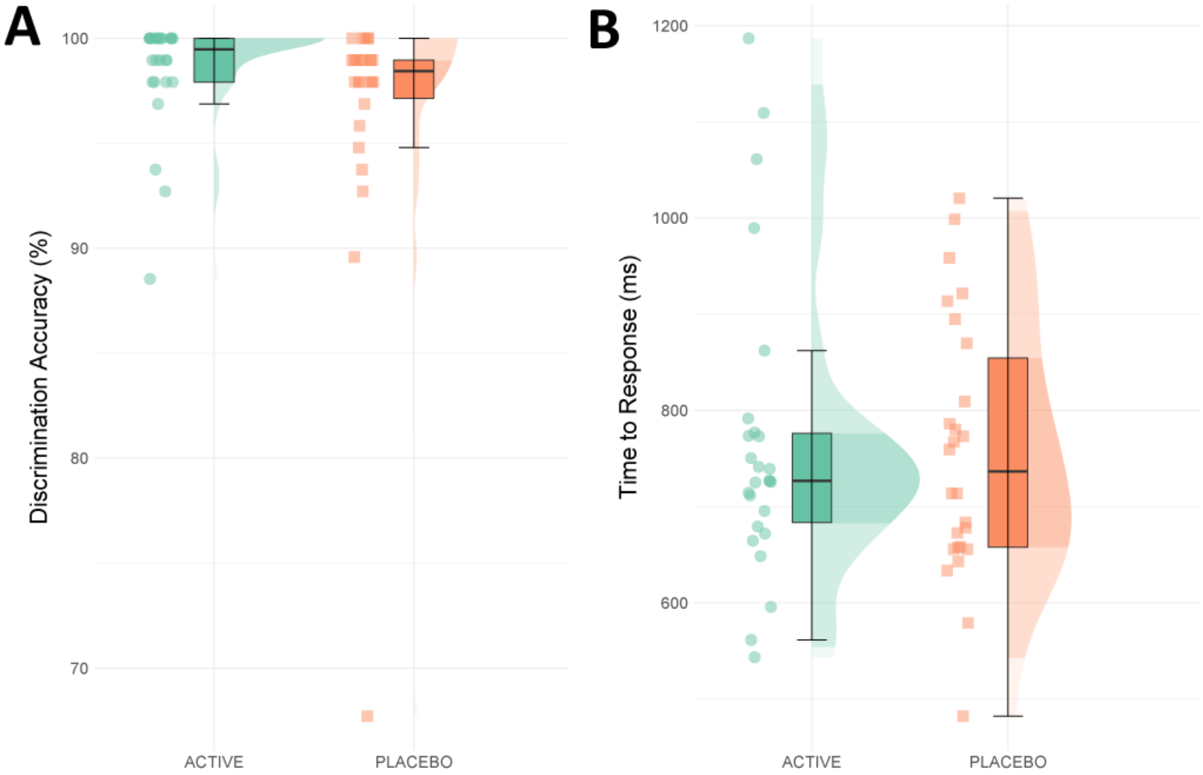

Supplementary Fig. 2. Discrimination accuracy and response time across allocation groups during in-scanner encoding task. A Mean percentage accuracy of discrimination between labelling stimuli ('animal' or 'landscape') across allocation groups. **B** Mean response time (ms; milliseconds) to choice during discrimination across groups. This figure contains data from *N*=52 individuals; lines and plot points depict mean value, with error bars and shaded areas around each line depicting standard mean error.

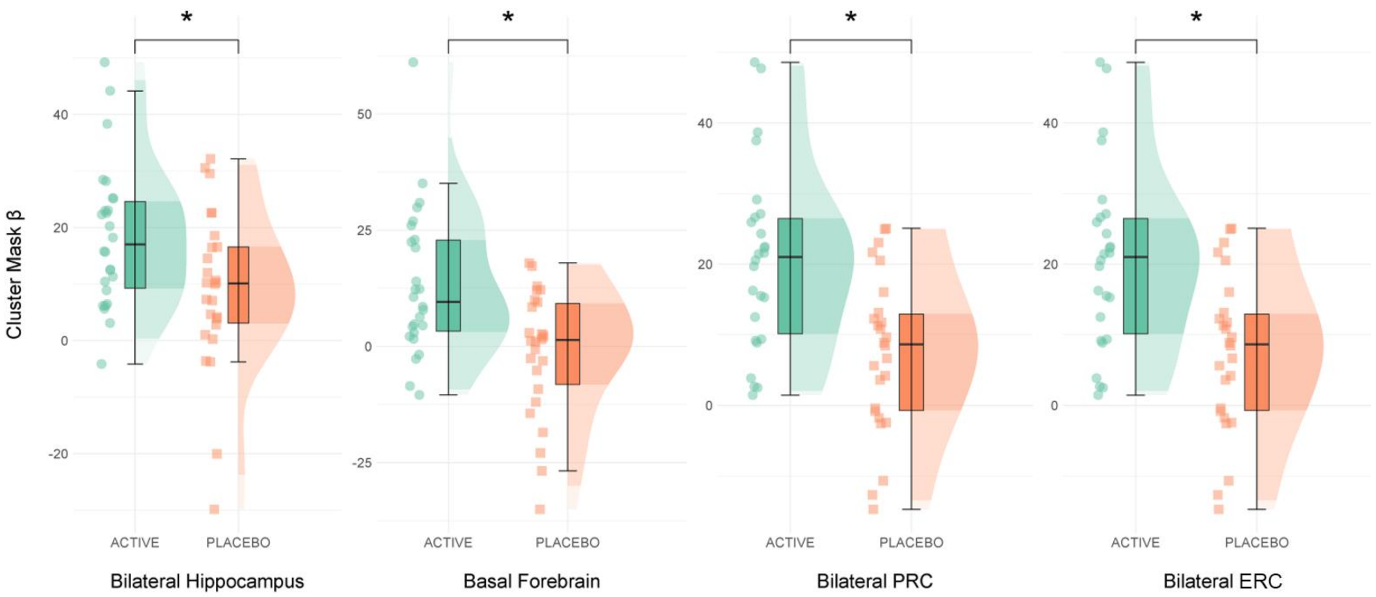

Supplementary Fig. 3. BOLD activation (β) in a priori regions of interest during novel memory formation across allocation groups. Parameter estimates were derived from pitolisant > placebo TFCE-corrected clusters. This figure contains data from *N*=52 individuals. Full statistics for TFCE-corrected permutation tests are reported in Supplementary Note 3. Boxplots represent interquartile range (IQR); central line depicts the median. Whiskers represent ±1.5 IQR, encompassing most data points; half-violin plots depict the data distribution. * $p \leq 0.05$ represents group differences by one-tailed permutation testing (TFCE corrected). Abbreviations: PRC = Perirhinal cortex; ERC = Entorhinal cortex.

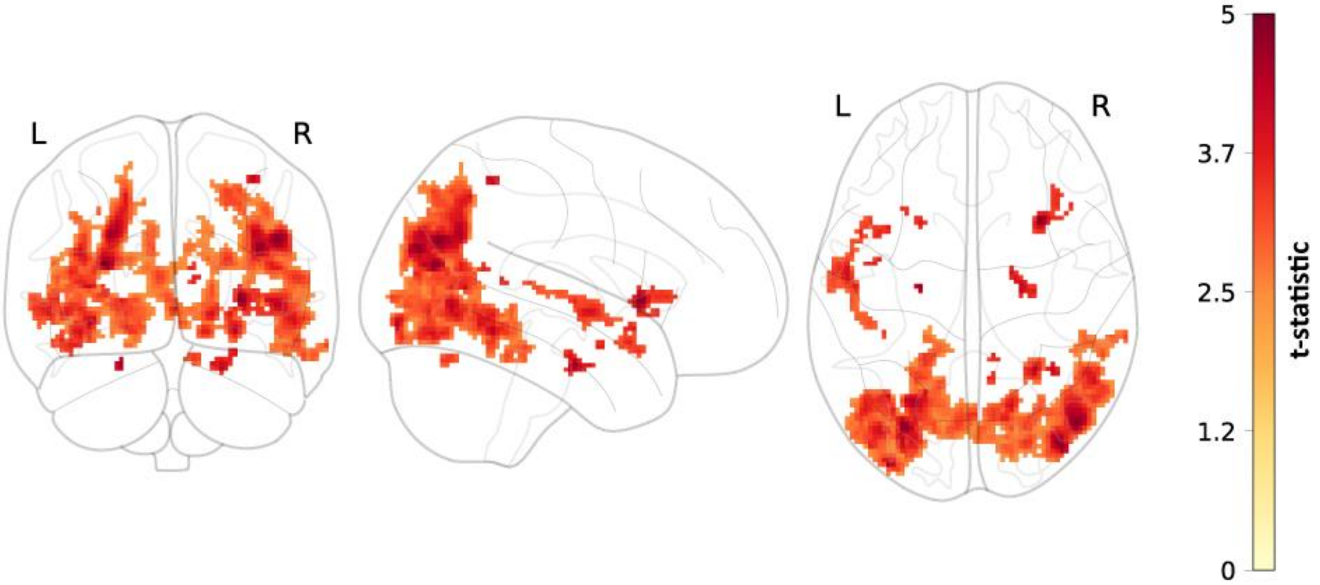

Supplementary Fig. 4. Whole brain analysis of activity during novel memory formation across groups. At the whole-brain level, increased activity was observed in the pitolisant group (pitolisant > placebo) during novel memory formation is represented in by the TFCE-corrected (*p < 0.05*) t-statistic map. This figure contains data from *N*=52 individuals. The location and characteristics of each cluster is described in Supplementary Table 7.

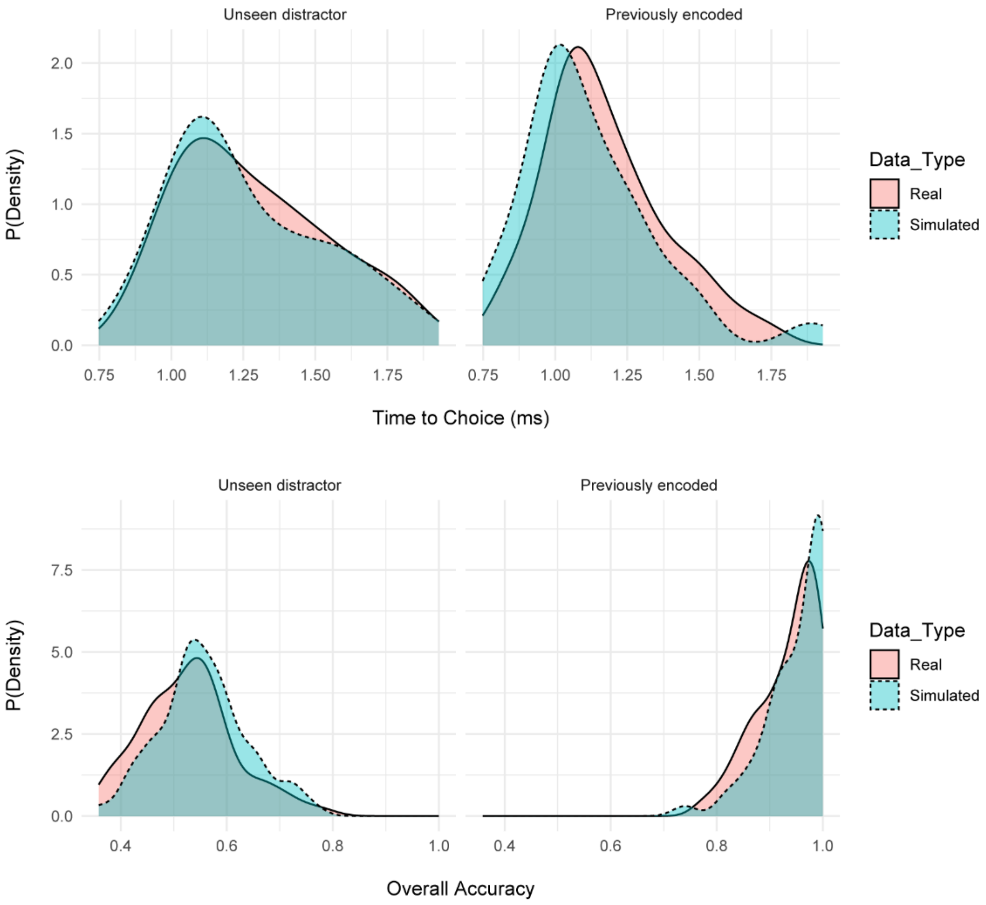

Supplementary Fig. 5. Memory recognition DDM: Posterior predictive checks. A Comparison of probability density curves between observed task data (red; solid line) and synthetic data generated from the model fitting process (green; dashed line), incorporating data from both study visits (baseline and follow-up). Synthetic data were generated from models fitted to data from *N* = 52 individuals (*N* = 104 total).

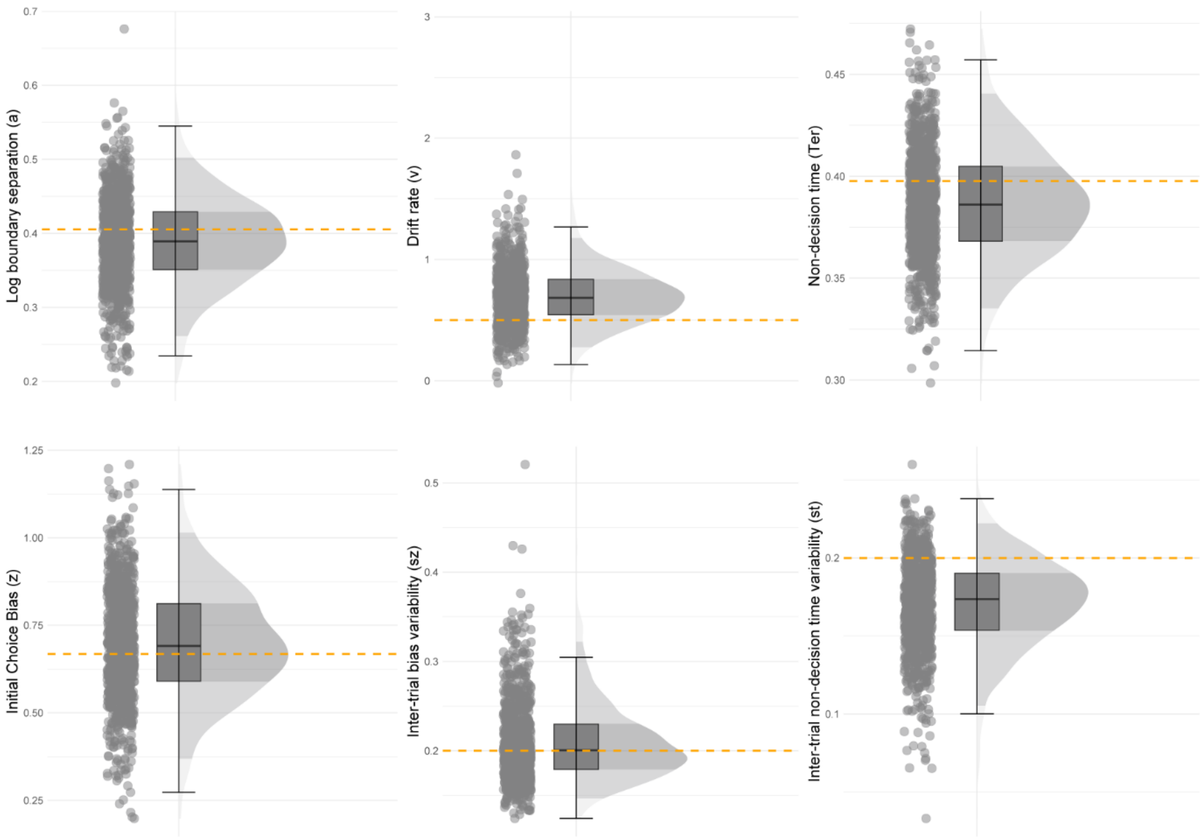

Supplementary Fig. 6. Memory recognition DDM: Drift diffusion model parameter recovery. Synthetic data were generated using true model parameters (yellow dashed line) and fit to the drift diffusion model. The recovered parameters from this process are plotted against their corresponding true values here. The data within figure contain 200,000 trials (or, *N*=1000 synthetic participants). Boxplots represent the interquartile range (IQR), while the central line depicts the median. The whiskers extend to approximately ± 1.5 times the IQR, encompassing the bulk of the data points; half-violin plots depict the data distribution.

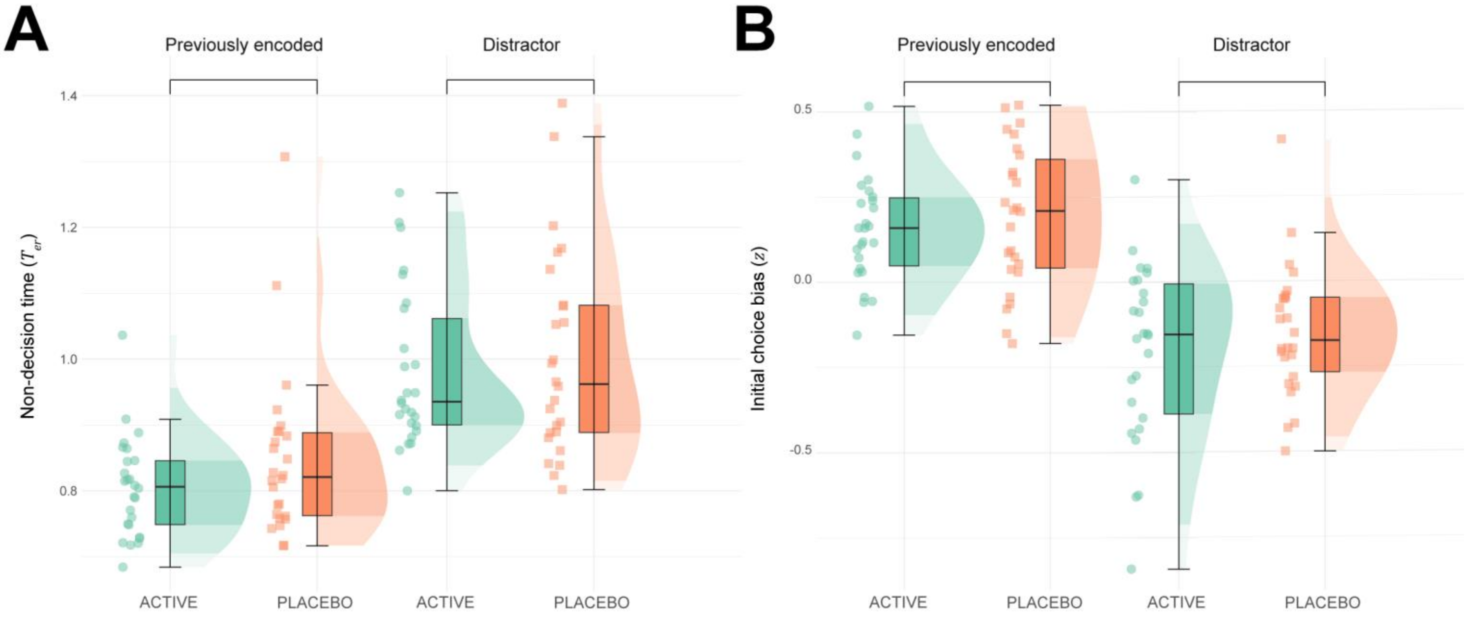

Supplementary Fig. 7. Memory Recognition DDM: non-decision time T_{er} and initial choice bias z parameters across allocation groups. A There was no significant main effect of group for T_{er} (F[1,50] = 1.23, *p* = 0.273), and no significant group × task condition interaction for T_{er} (F[1,50] = 0.41, *p* = 0.523). **B** Similarly, there was no significant main effect of group for z (F[1,50] = 1.63, *p* = 0.208), and no significant group × task condition interaction for z (F[1,50] = 0.09, *p* = 0.764). This figure contains data from *N*=52 individuals; lines and plot points depict mean value, with error bars and shaded areas around each line depicting standard mean error.

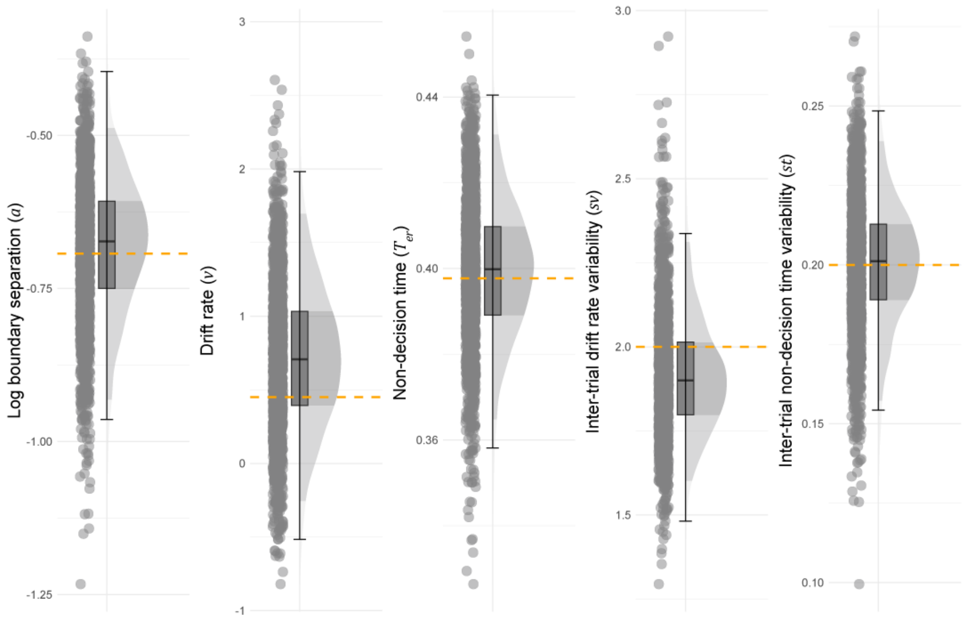

Supplementary Fig. 8. Complex working memory task (*n*-back): Drift diffusion model parameter recovery and split non-decision time posterior predictive checks. A Synthetic data were generated using true model parameters (yellow dashed line) and fit to the drift diffusion model. The recovered parameters from this process are plotted against their corresponding true values here. The data within figure contain 200,000 trials (or, *N*=500 synthetic participants). Boxplots represent the interquartile range (IQR), while the central line depicts the median. The whiskers extend to approximately ± 1.5 times the IQR, encompassing the bulk of the data points; half-violin plots depict the data distribution**.**

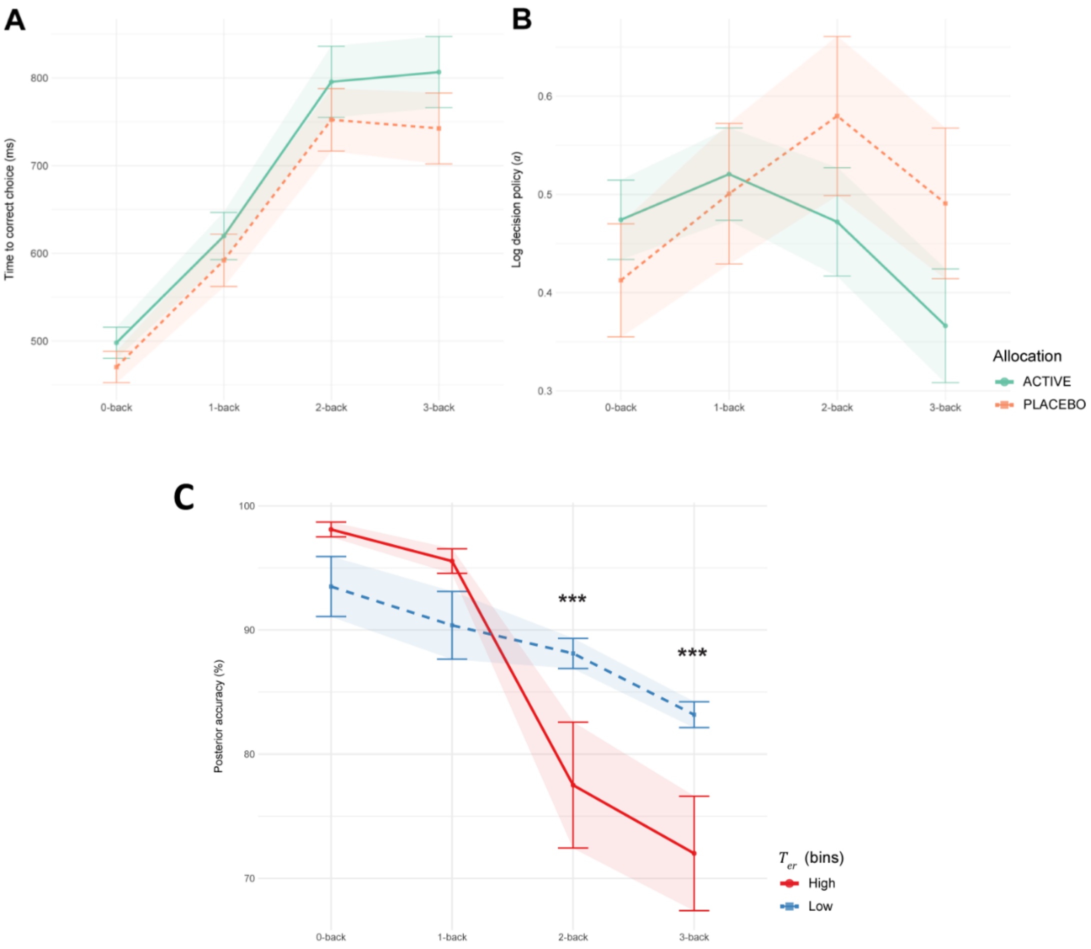

Supplementary Fig. 9. Additional n-back analyses: time to correct choice (ms), decision policy a parameter and split T_{er} analysis. A There was no significant main effect of group on time to choice (ANOVA: F[1,50] = 1.02, p = 0.317), in addition to no significant group x task condition interaction (F[3, 150] = 0.44, *p* = 0.722). **B** Similarly, there was no significant main effect of group on decision policy a (F[1,50] = 0.289, *p* = 0.593), in addition to no significant group x task condition interaction (F[3,150] = 1.905, *p* = 0.131). **C** In addition to the median-split T_{er} analysis on posterior accuracy (see Results, main text), we repeated the approach on observed accuracy values. This confirmed a T_{er} × task complexity interaction on observed accuracy (F[3,149] = 2.857, p = 0.0391, η_p^2 = 0.13 [0.05, 0.22]). Significant pairwise differences were seen at medium-hard task difficulty, but not control and easy difficulty (0-back EMM = -0.09 ± 0.04, *p* = 0.248; 1-back EMM = 0.02 ± 0.03, *p* = 0.1552; 2-back EMM = -0.07 ± 0.03, *p* = 0.0003, *d* = -0.85 [-1.92, -0.52]; 3-back EMM = -0.10 ± 0.03, *p* = 0.0003, *d* = -1.18 [-2.12, -0.83]). All panels contain data from *N*=52 individuals; lines and plot points depict mean value, with error bars and shaded areas around each line depicting standard mean error.

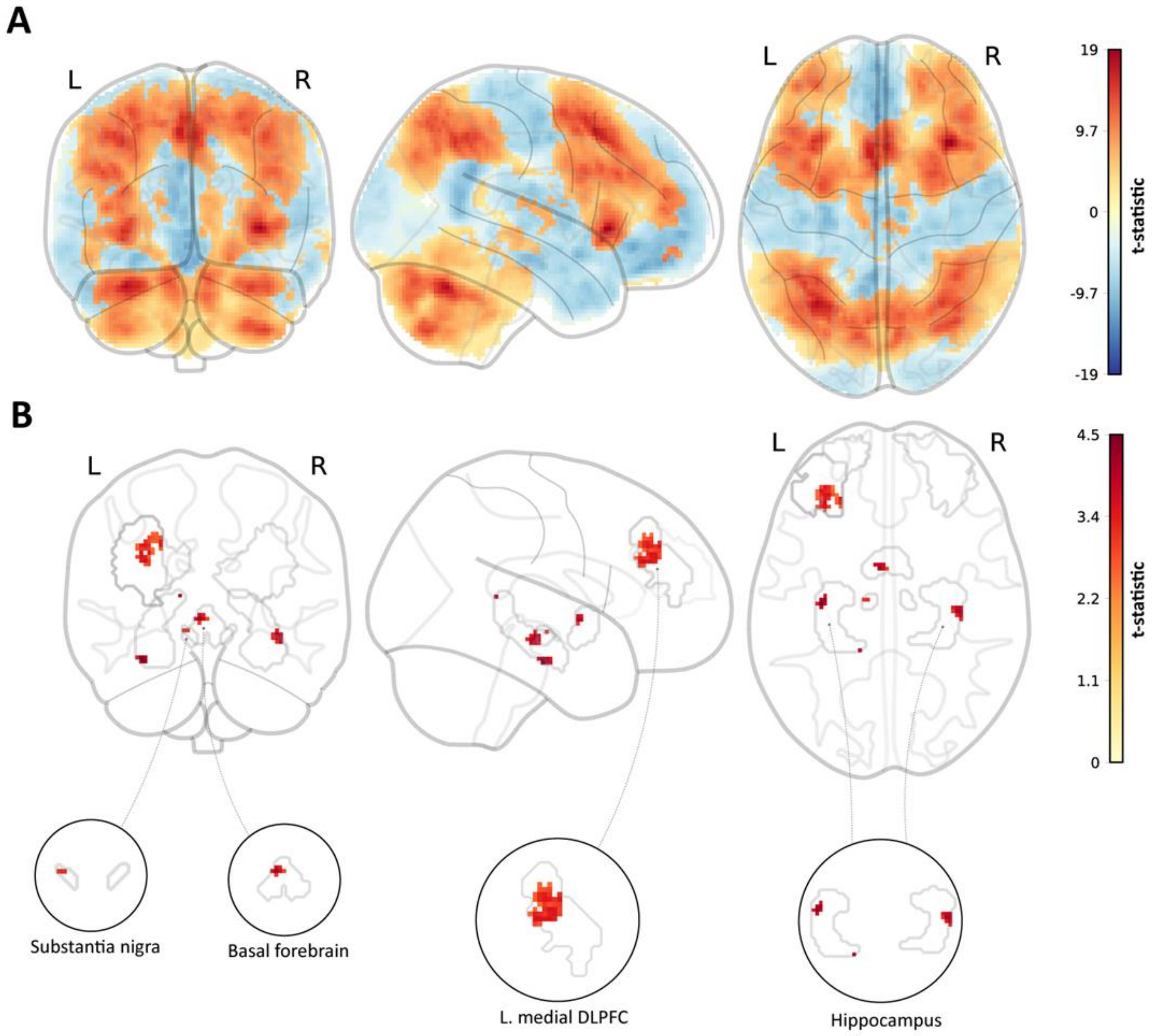

Supplementary Fig. 10. Functional imaging analysis of the complex working memory task. A Whole brain analysis (via permutation testing) of the effect of task is displayed for the primary contrast. TFCE-corrected t-statistic maps show increased (2 > 0-back) and decreased (0 > 2-back) BOLD activity during complex working memory processing across a network of structures including the dorsolateral prefrontal cortex, cerebellum, thalamus and brain stem (whole brain 2 > 0-back cluster: peak MNI coordinates = X,Y,Z [-6, -56, -62]; cluster size (voxels) = 79,786; t-max(51) = 3.320, p = 0.0002, TFCE-corrected; whole brain 0 > 2-back cluster 1: peak MNI coordinates = [-48, -2, -46]; cluster size = 50,848; t-max(51) = 3.810, p = 0.0002, TFCE-corrected; whole brain 0 > 2-back cluster 2: peak MNI coordinates = [-20, -104, 8]; cluster size = 1,842; t-max(51) = 6.529, p = 0.0014, TFCE-corrected; whole brain 0 > 2-back cluster 3: peak MNI coordinates = [-50, -64, 34]; cluster size = 260; t-max(51) = 6.245, p = 0.0120, TFCE-corrected; whole brain 0 > 2-back cluster 4: peak MNI coordinates = [34, -78, -34]; t-max(51) = 8.022, p = 0.0154, TFCE-corrected). **B** Increased activity during complex working memory processing (2 > 0-back) in the pitolisant group (pitolisant > placebo; SVC permutation testing) across *a priori* ROIs (significant clusters displayed in tan) including the medial DLPFC, bilateral hippocampus, basal forebrain and substantia nigra (see Supplementary Table 6 and Supplementary Fig. 7 for further information); *t*-statistics maps are TFCE-corrected. Enlarged ROIs are provided for visual purposes, with the ROI mask displayed in the gray outline. Panels **A** – **B** contain data from *N*=52 individuals. Abbreviations: BOLD = Blood Oxygenation Level Dependent; DLPFC = Dorsolateral Prefrontal Cortex; TFCE = Threshold-Free Cluster Enhancement; ROI = Region of Interest.

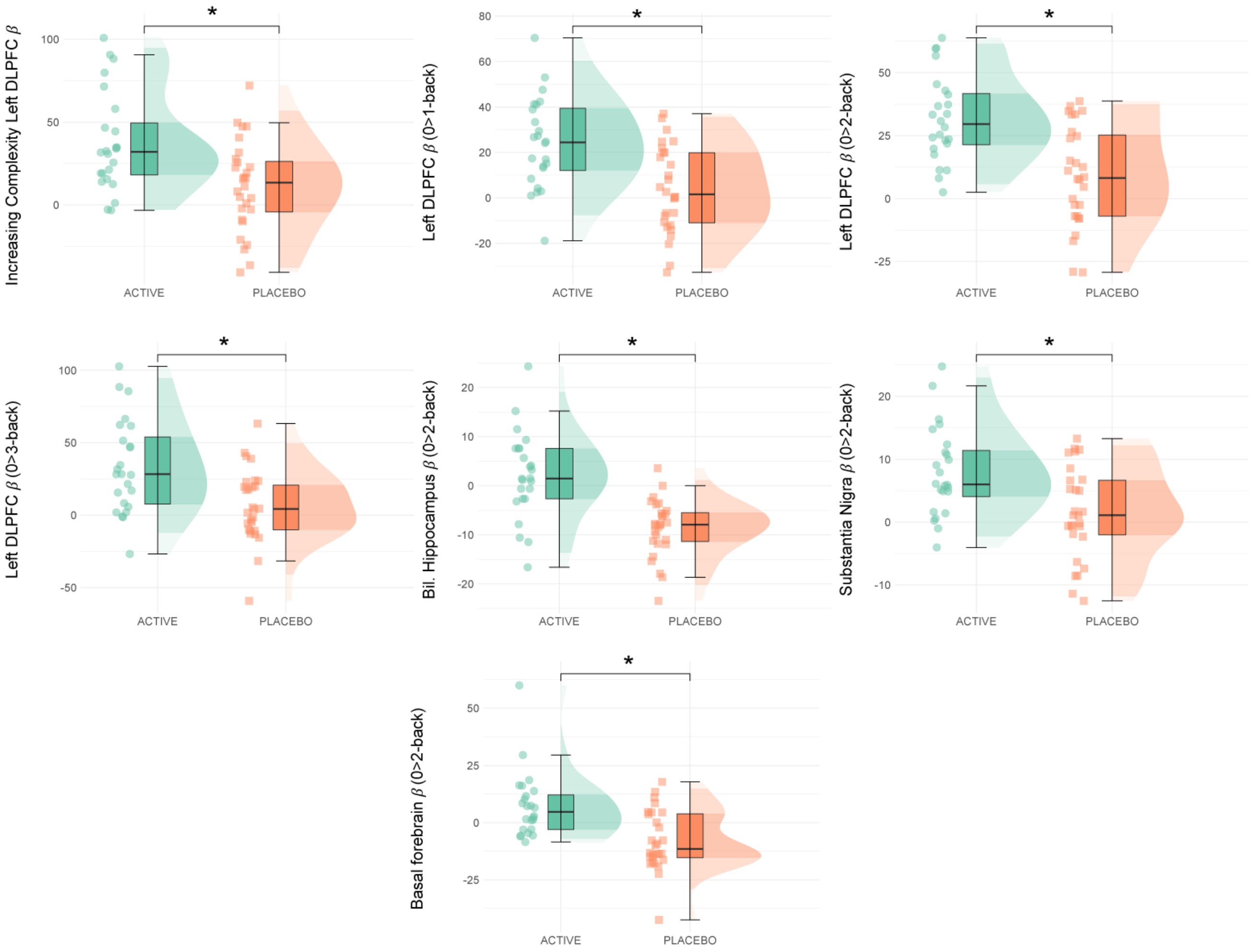

Supplementary Fig. 11. BOLD activation (β) in a priori regions of interest during the complex working memory task across allocation groups. Parameter estimates were derived from pitolisant > placebo TFCE-corrected clusters. This figure contains data from *N*=52 individuals. Boxplots represent interquartile range (IQR); central line depicts the median. Whiskers represent ±1.5 IQR, encompassing most data points; half-violin plots depict the data distribution. * p ≤ 0.05 represents group differences by one-tailed permutation testing (TFCE corrected). Abbreviations: DLPFC = Dorsolateral prefrontal cortex.

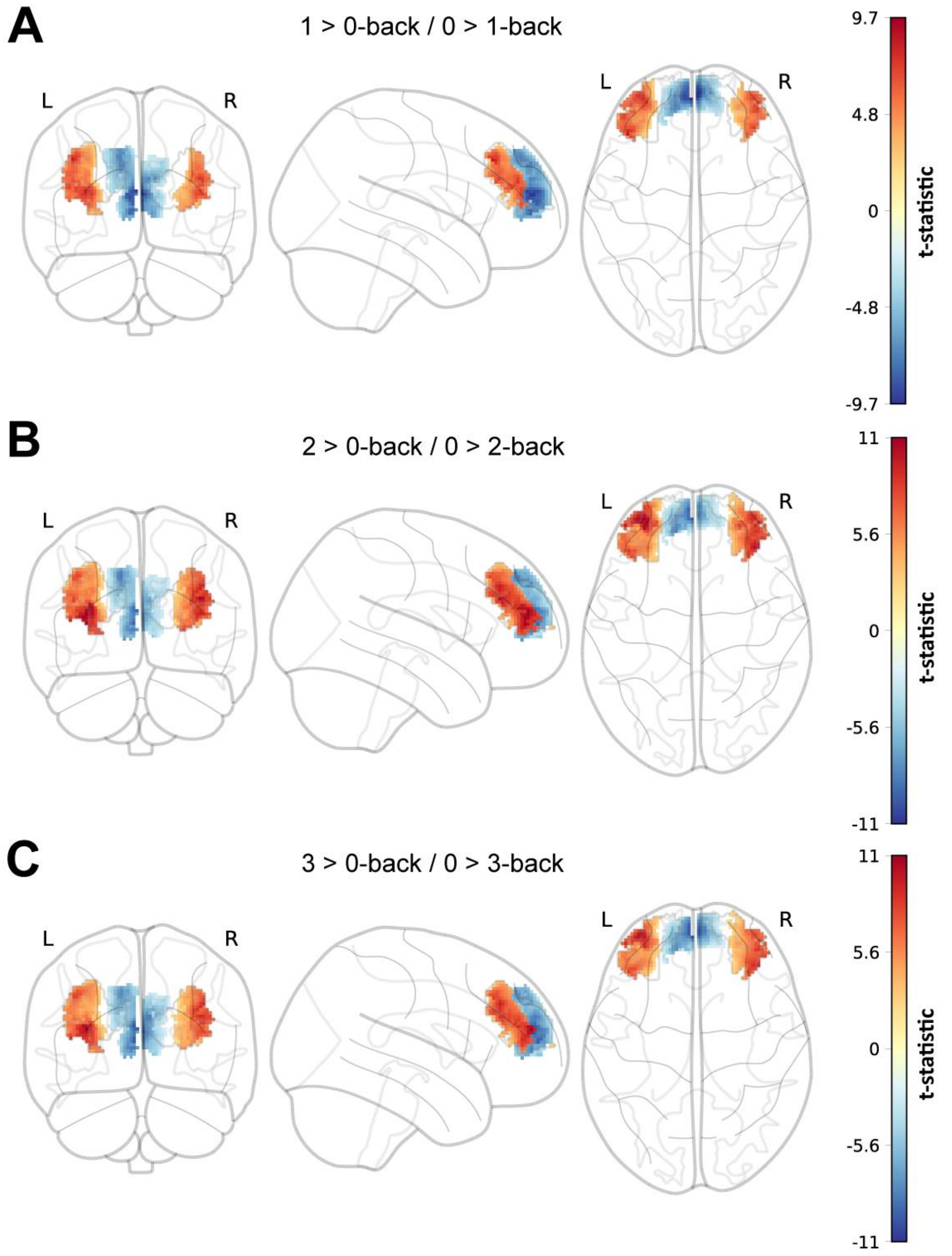

Supplementary Fig. 12. N-Back fMRI paradigm: main effect of task across each level of task complexity. Small volume corrected analysis of the effect of task on medial and rostral DLPFC activity across each level of task complexity (**A**: 1 > 0-back / 0 > 1-back; **B**: 2 > 0-back / 0 > 2-back; **C**: 3 > 0-back / 0 > 3-back). TFCE-corrected ($p < 0.05$) t-statistic maps show increased (1/2/3 > 0-back) and decreased (0 > 1/2/3-back) BOLD activity during complex working memory processing. Increased DLPFC peaked in the 2 > 0-back condition by number of voxels activated (3 > 0-back cluster left DLPFC cluster: peak MNI coordinates = X,Y,Z [-28, 48, 4]; cluster size = 893; t-max (51) = 5.280, $p < 0.001$, TFCE-corrected; 3 > 0-back cluster right DLPFC cluster: peak MNI coordinates: [28, 50, 6]; cluster size = 837; t-max (51) = 5.284, $p < 0.001$, TFCE-corrected; 2 > 0-back left DLPFC cluster: peak MNI coordinates = [-28, 48, 4]; cluster size = 977; t-max (51) = 7.252, $p < 0.001$, TFCE-corrected; 2 > 0-back right DLPFC cluster: peak MNI

coordinates = [28, 50, 6]; cluster size = 871; *t*-max (51) = 8.145, $p < 0.001$, TFCE-corrected; 1 > 0-back left DLPFC cluster: peak MNI coordinates = [-32, 50, 6]; cluster size = 738; *t*-max (51) = 4.997, $p < 0.001$, TFCE-corrected; 1 > 0-back right DLPFC cluster: peak MNI coordinates = [32, 54, 8]; cluster size = 552; *t*-max (51) = 5.301, $p < 0.001$, TFCE-corrected). Further, decreases in DLPFC activity were most pronounced in the 0 > 1-back condition by number of voxels activated (0 > 3-back left DLPFC cluster: peak MNI coordinates = [-6, 50, 0]; cluster size = 735; *t*-max = 6.805, $p < 0.001$, TFCE-corrected; 0 > 3-back right DLPFC cluster: peak MNI coordinates = [10, 58, 2]; cluster size = 622; *t*-max (51) = 4.539, $p < 0.001$, TFCE-corrected; 0 > 2-back left DLPFC cluster: peak MNI coordinates = [-6, 50, 0]; cluster size = 717; *t*-max (51) = 6.974, $p < 0.001$, TFCE-corrected; 0 > 2-back right DLPFC cluster: peak MNI coordinates = [2, 52, 6]; cluster size = 573; *t*-max (51) = 4.460, $p < 0.001$, TFCE-corrected; 0 > 1-back left DLPFC cluster: peak MNI coordinates = [-6, 50, 0]; cluster size = 747; *t*-max (51) = 6.064, $p < 0.001$, TFCE-corrected; 0 > 1-back right DLPFC cluster: peak MNI coordinates = [12, 52, 2]; cluster size = 672; *t*-max (51) = 4.218, $p < 0.001$, TFCE-corrected). All panels contain data from N=52 individuals.

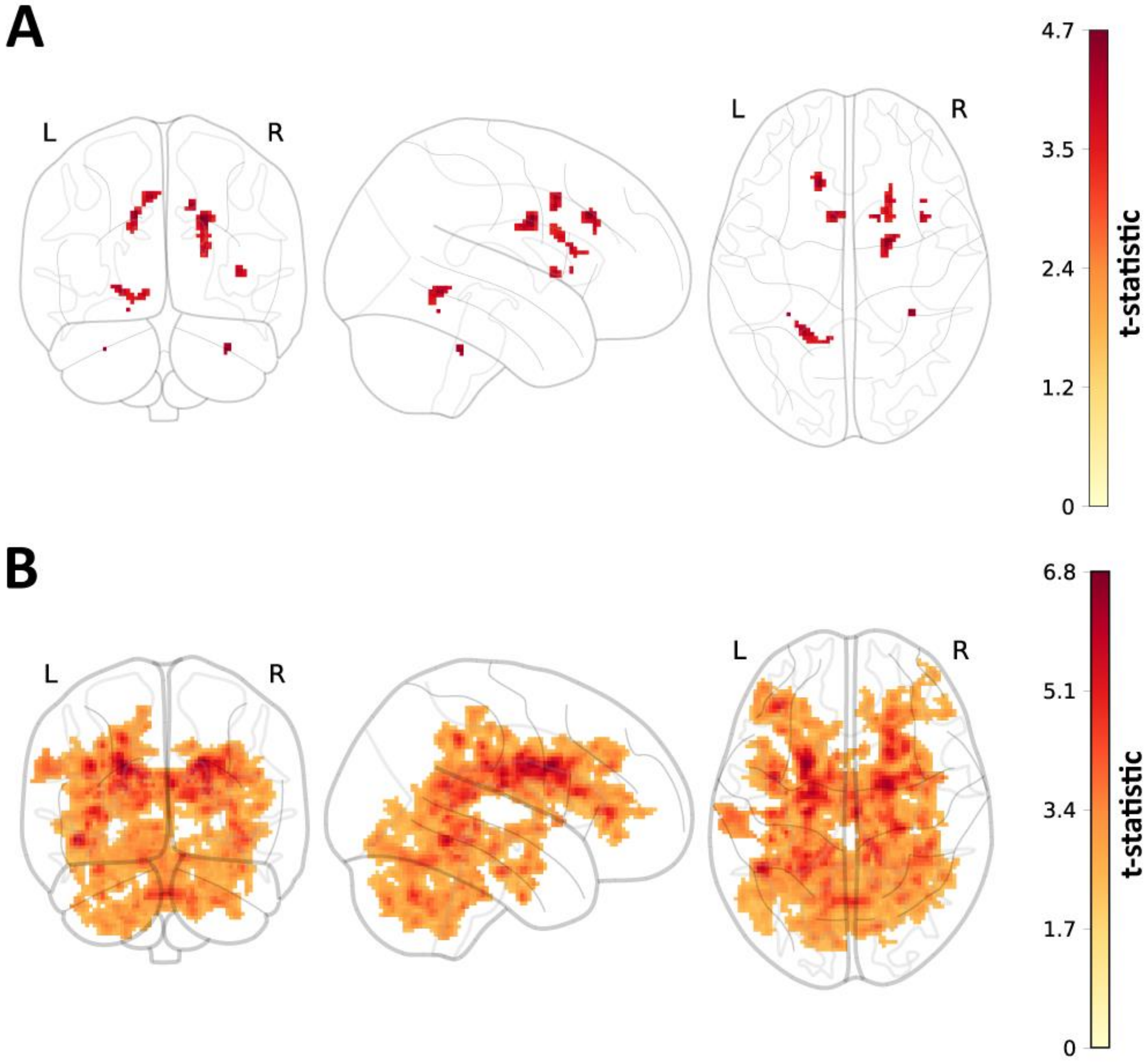

Supplementary Fig. 13. Whole brain analysis of activity during complex working memory across groups. At the whole-brain level, increased activity was observed in the pitolisant group (pitolisant > placebo) during during 1 > 0-back (**A**) and 2 > 0-back (**B**) is represented in by the TFCE-corrected (*p < 0.05*) t-statistic map. No significant clusters were identified in the whole-brain analysis of the 3 > 0-back contrast. The location and characteristics of each cluster is described in Supplementary Table 4. All panels contain data from *N*=52 individuals.

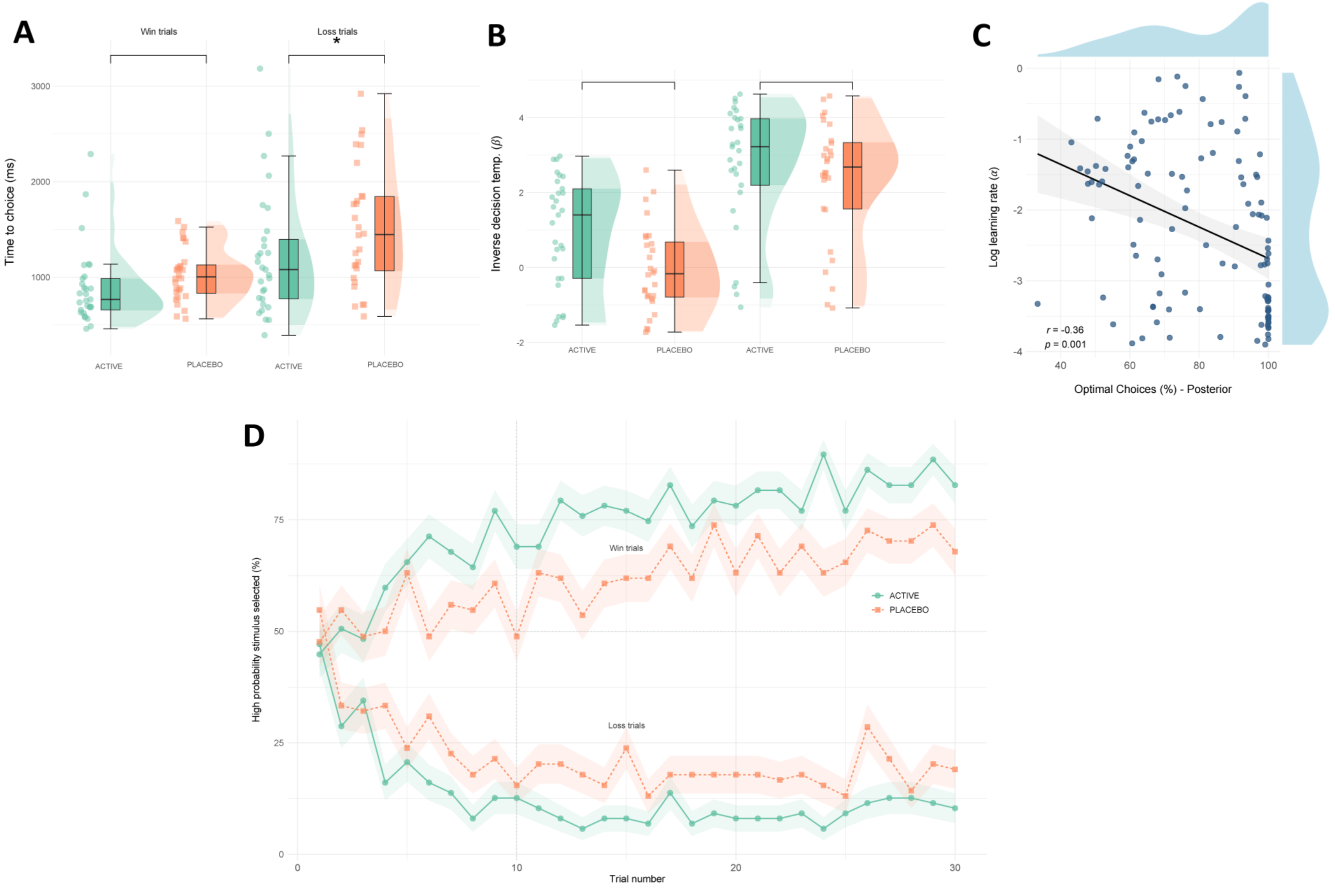

Supplementary Fig. 14. Additional analyses - Probabilistic Instrumental Learning Task. A There was no significant main effect of group on time to choice (ms) during the task (F[1,55] = 3.263, *p* = 0.0763), in addition to no significant group × task condition interaction (F[1,55] = 2.02, *p* = 0.16). **B** Similarly, there was no significant effect of group on inverse decision temperature during the task (F[1,42] = 2.40, *p* = 0.1287), in addition to no significant group × task condition interaction (F[1,52] = 0.35, *p* = 0.5580). Boxplots represent the interquartile range (IQR), while the central line depicts the median. The whiskers extend to approximately ± 1.5 times the IQR, encompassing the bulk of the data points; half-violin plots depict the data distribution. C Model-fitted posterior for optimal choices were associated with learning rate (β = -5.55, *p* = 0.00142, η_p^2 = 0.16 [0.07, 1.00]; *r* = -0.36); this relationship was observable within allocation groups, based on aggregated posterior estimates (β = -5.09 [-7.00, -3.19]). The fitted line through data points was generated via linear modelling (shaded area: standard mean error). **D** Synthetic temporal learning was generated using posterior choice probabilities and simulating choices from a Bernoulli distribution. Synthetic learning over time across groups (averaged across blocks) are displayed here – see Figure 5B (main text) for comparison with observed data. The Y axis (‘high probability stimulus selected’) refers to the selection (mean %) of high probability win or loss symbols. Plot points depict mean value; error bars and shaded area depict SEM. All panels include data from *N*=57 individuals.

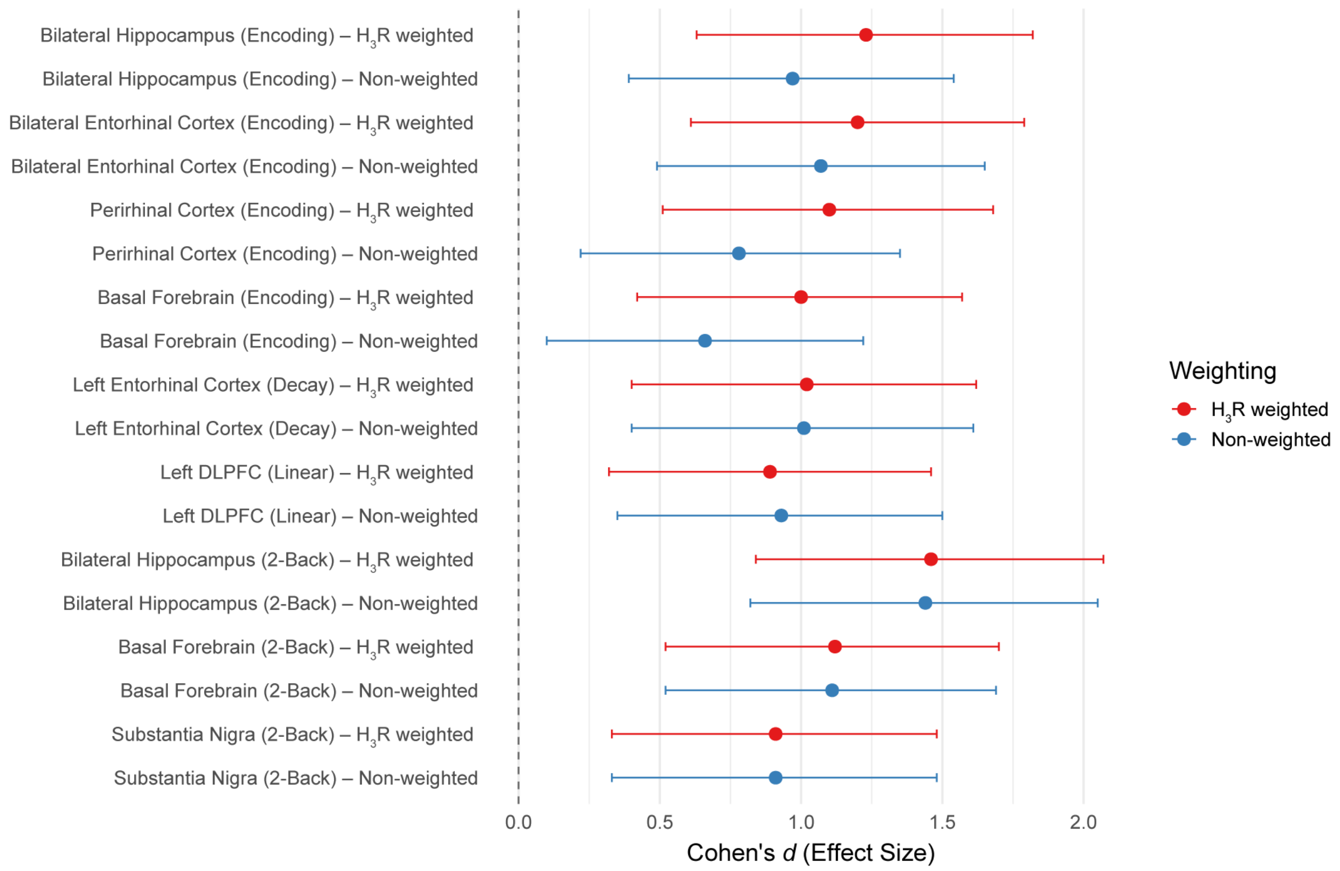

Supplementary Fig. 15. Summary of fMRI task-based group effect cluster effect sizes: H₃R-weighting vs non-weighting. Throughout the fMRI task-based analysis (memory encoding and n-back fMRI; *N*=52 individuals), multiple group-based effects (drug > placebo) were identified via TFCE-corrected permutation testing. BOLD signal within the cluster (alpha thresholded; $p < 0.05$) was extracted within using both binary and H_3R-weighted cluster masks. Central line dots represent mean Cohen's *d*, and whiskers represent the 95% confidence interval.

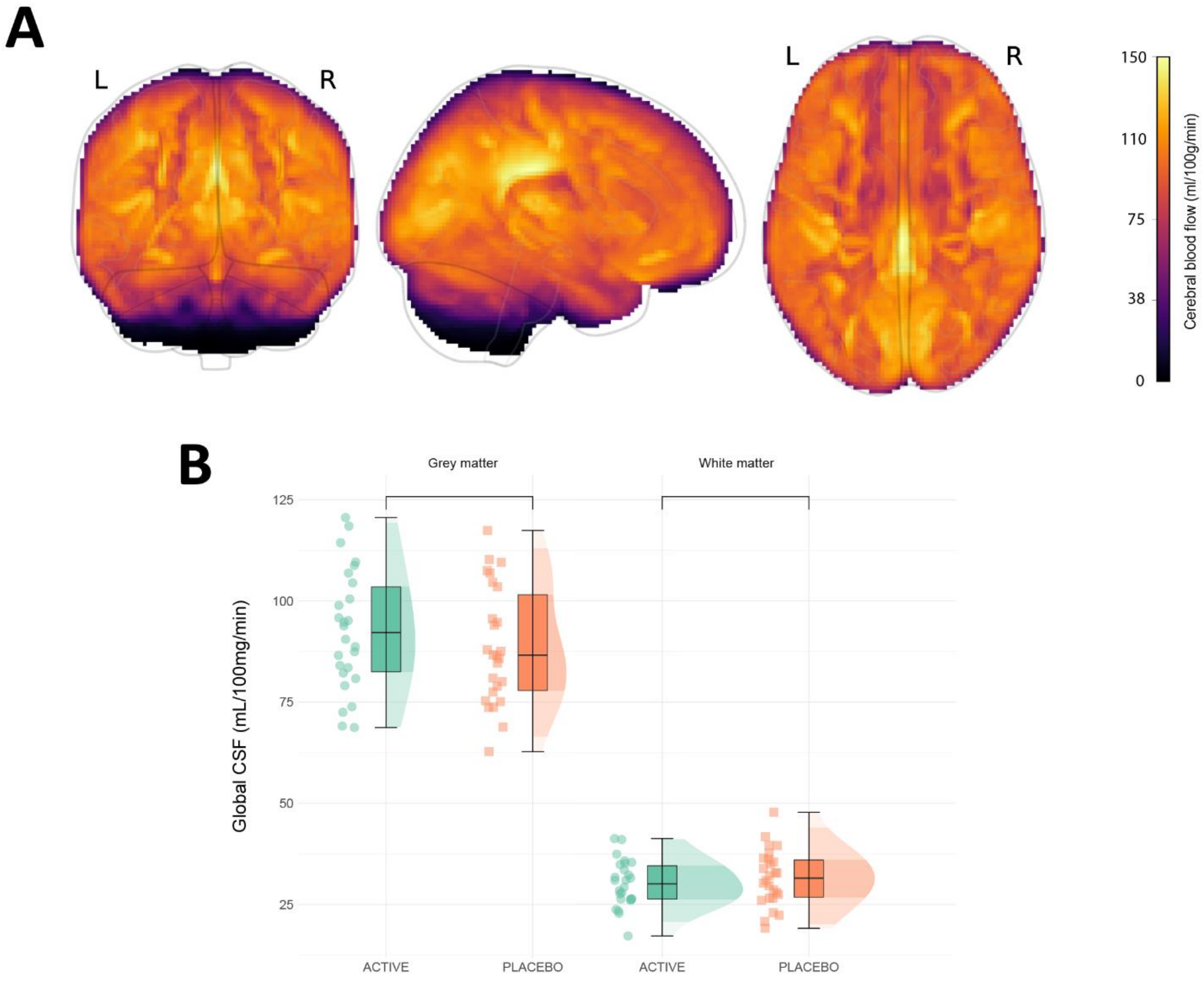

Supplementary Fig. 16. Arterial Spin Labelling – Cerebral Blood Flow Analysis. A Mean Cerebral blood flow across all participants. **B** Global cerebral blood flow values (grey and white matter) **across allocation groups.** All panels contain data from *N*=52; boxplots represent the interquartile range (IQR), while the central line depicts the median. The whiskers extend to approximately ± 1.5 times the IQR, encompassing the bulk of the data points; half-violin plots depict the data distribution.

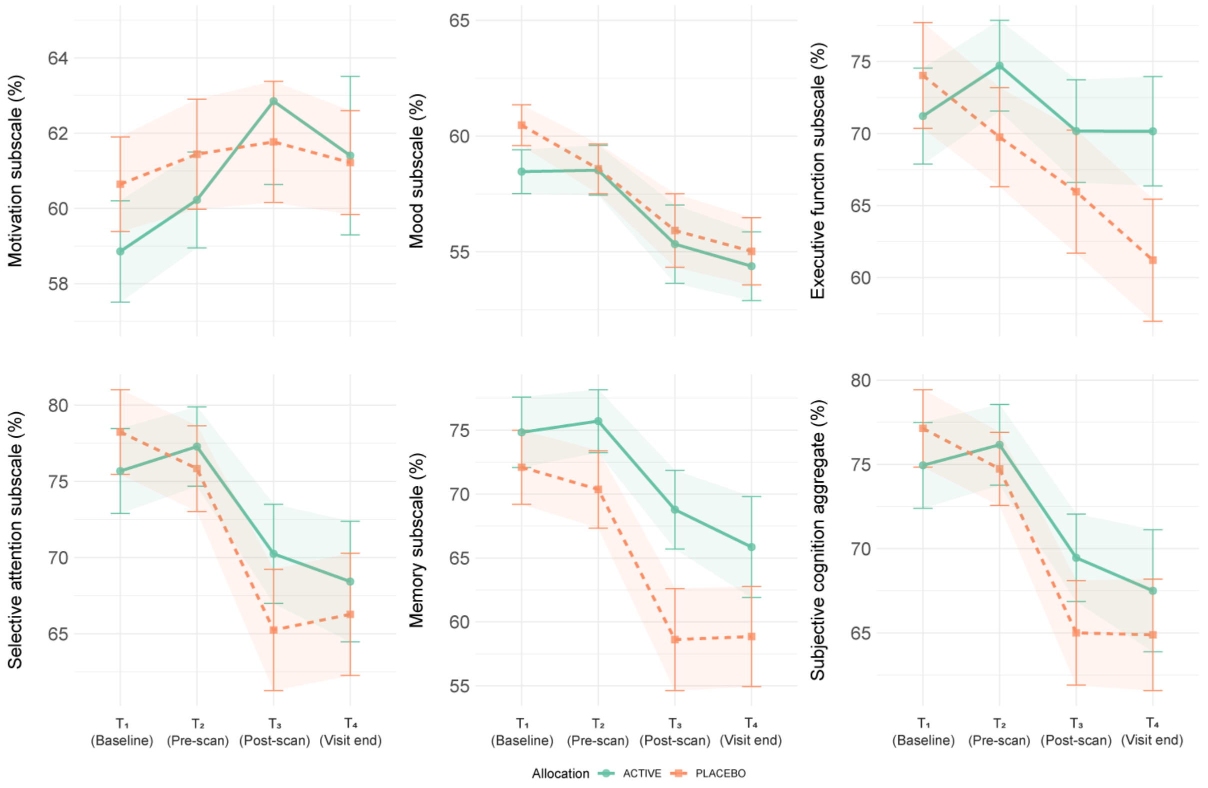

Supplementary Fig. 17. Subjective cognition throughout the acute dosing period across allocation groups. Throughout the acute dosing period, individuals were asked to self-rate subjective cognition at regular intervals (T_1: Baseline, before intervention; T_2 pre-scan, 2.5hrs post-intervention; T_3 post-scan, 4hrs post-intervention; T_4 at visit end, 5hrs post-intervention). Scores for each allocation group are plotted across all timepoints, where higher scores indicate greater self-assessment of cognitive ability, and were divided into five subscales: motivation, mood, executive functioning, selective attention, and memory. Lines and plot points depict mean value, with error bars and shaded areas around each line depicting standard mean error. This figure contains data for *N*=57 individuals (216 observations total). *N*=1 individuals were excluded from analyses due to data missingness (baseline).

Supplementary Tables

Supplementary Table 1. Study inclusion and exclusion eligibility criteria

Inclusion criteria:<br>• Participant is willing and able to give informed consent for participation in the research<br>• Not currently taking any medications which may interfere with pitolisant, including psychoactive medications<br>• Not currently using antihistaminergic medication<br>• Aged 18-45 years<br>• Male or female<br>• Sufficiently fluent English to understand and complete cognitive tasks and questionnaires<br>• Body Mass Index within the range 18–30<br>• Right handed
Exclusion criteria:<br>• Current pregnancy (as determined by urine pregnancy test taken during screening visit), planning to become pregnant or breast feeding<br>• Any past or current history of severe and/or serious psychiatric disorder, including but not limited to schizophrenia, psychosis, bipolar affective disorder, major depressive disorder, obsessive compulsive disorder<br>• Clinically significant abnormal values for urine drug screen, blood pressure measurement (in accordance with AP20 'non-invasive blood pressure'). A participant with a clinical abnormality or parameters outside the reference range for the population being studied may be included only if the Investigator considers that the finding is unlikely to introduce additional risk factors and will not interfere with the study procedures<br>• History of, or current medical conditions which, in the opinion of the investigator, may interfere with the safety of the participant or the scientific integrity of the study, including epilepsy/seizures, brain injury, hepatic or renal disease, acid-related gastro-intestinal problems, Central Nervous System (CNS) tumours, neurological conditions<br>• Current or past history of drug or alcohol dependency<br>• Severe lactose intolerance<br>• Use of recreational drugs (e.g. cannabis, cocaine, amphetamines) within past 3 months<br>• Participation in a study which uses the same computer tasks as those in the present study (determined by asking participants about previous studies participated in during screening) within past 3 months<br>• Participation in a study that involves the use of a medication within the last three months<br>• Smoking > 5 cigarettes per day<br>• Consumption of a high amount of caffeine per day (> 400ml caffeine) (*e.g.,* 5 or more cups of coffee)<br>• Participant is unlikely to comply with the clinical study protocol or is unsuitable for any other reason, in the opinion of the Investigator<br>• Any contraindication to MRI scanning (e.g. metal objects in your body, pacemakers, significant claustrophobia)

Table 2. Demographic characteristics across allocation groups

	Pitolisant (*n*=29)	Placebo (*n*=29)	Inferential analysis [a]
Age (Years), M (S.D.)	27.14 (5.57)	29.21 (6.72)	0.21
Gender, *N* male:female	10:19	11:18	1.00
Body mass index, M (S.D.)	23.90 (3.49)	23.59 (3.23)	0.73
Contraceptive use, yes:no	6:13	5:13	1.00
Native language, *N*			0.67
English	21	23	0.50
Chinese	2	1	
Spanish	1	3	
Other	5	2	
Digit span aggregate	36.83 (5.67)	36.10 (6.10)	0.64
Time in education (Years), M (S.D.)	17.17 (2.82)	17.97 (2.64)	0.27
Highest educational attainment, *N*			0.71
High School / Sixth form	5	6	
Undergraduate degree	12	8	
Postgraduate (Masters)	8	11	
Postgraduate (Doctorate)	4	4	

[a] Values represent significance values inferential analyses. These values pertain to Welch's two Sample t-test where differences between group means were analysed, and Pearson's Chi-squared where differences between frequency/ratio distributions were analysed.

Supplementary Table 3. Memory encoding fMRI task: TFCE-corrected cluster summary for main effect of task.

Task contrast	Cluster	Location(s)*	Hemisphere / division (peak)	Peak voxel coordinates (MNI)			Cluster size (voxels)	*t*-value (peak)	*p*-value (peak)
				X	Y	Z			
Novel > Familiar									
	1	Cerebellum; Bilateral hippocampus; bilateral putamen; bilateral occipital cortex; bilateral amygdala; bilateral thalamus; bilateral pallidum; bilateral precentral gyrus; brain stem; bilateral frontal poles; bilateral Inferior frontal gyrus	Left	-32	-70	-54	62965	3.773	0.0002
	2	Left premotor cortex	Left	-4	8	62	455	6.365	0.0100
	3	Bilateral anterior cingulate cortex	Right	6	4	28	123	5.985	0.0206
Familiar > Novel									
	1	Bilateral precuneous cortex	Right	12	-64	26	6380	6.522	0.0002
	2	Lateral Occipital Cortex; angular gyrus	Right	54	-62	40	1198	6.125	0.0002
	3	Frontal pole	Right	14	54	-2	128	4.742	0.0002
	4	Frontal pole	Left	-16	94	37	60	5.950	0.0002
	5	Middle frontal gyrus	Right	36	26	44	12	5.299	0.0020

Note: Statistical testing via TFCE-corrected permutation testing. Only clusters which remained significant following correction ($p < 0.05$, two-tailed) were included. *Cortical or subcortical regions falling within the significant cluster.

Supplementary Table 4. Memory encoding task: Between-groups whole-brain fMRI analysis

Task contrast	Cluster	Location(s)	Hemisphere / division (peak)	Peak voxel coordinates (MNI)			Cluster size (voxels)	*t*-value (peak)	*p*-value (peak)
				X	Y	Z			
Novel > Familiar									
	1	Lateral occipital cortex (superior); Visual cortex (V7)	Right	46	-74	28	4563	4.957	0.0160
	2	Superior, temporal and inferior temporal gyri	Left	-54	-8	-2	180	4.002	0.0400
	3	Lingual and fusiform gyri	Right	18	-66	-4	131	3.981	0.0408
	4	Fusiform gyrus	Right	28	-50	-10	101	4.291	0.0366
	5	Insular cortex	Right	30	16	2	92	4.825	0.0346
	6	Hippocampus; parahippocampal gyrus (anterior); subiculum; entorhinal cortex	Right	22	-16	-28	40	4.256	0.0432
	7	Temporal pole	Left	-48	10	-16	33	3.623	0.0470
	8	Cerebellum (VI)	Right	10	-74	-24	25	2.518	0.0524
	9	Insular cortex	Left	-36	12	-10	16	3.721	0.0476
	10	Lateral occipital cortex	Left	-40	-72	36	13	2.808	0.0494
	11	Superior parietal lobule	Right	36	-50	54	11	4.089	0.0470
	12	Planum polare	Left	-42	-4	-18	9	3.643	0.0488
	13	Putamen	Left	-22	14	2	9	3.745	0.0488
	14	Hippocampus; subiculum	Left	-22	-14	-26	7	4.651	0.0470
	15	Posterior cingulate gyrus	Right	8	-54	16	6	3.594	0.0488
	16	Insular cortex	Left	-28	18	4	5	3.421	0.0500
	17	Posterior cingulate gyrus	Right	10	-46	12	5	3.667	0.0486
	18	Lateral occipital cortex (superior)	Left	-32	-78	40	3	2.929	0.0496
	19	Planum temporale	Left	-38	-34	10	2	3.328	0.0496
Familiar > Novel									
	NA	--	--	--	--	--	--	--	--

Note: Statistical testing via TFCE-corrected permutation testing. Only clusters which remained significant following correction ($p < 0.05$, two-tailed) were included.

Supplementary Table 5. Post-encoding memory recognition task: Drift Diffusion Model comparison

Model specification	Deviance information criterion (DIC)
$a + v(single) + T_{er}$	10457.33
$a + v(stim) + T_{er}$	9131.99
$a(stim) + v(stim) + T_{er}(single)$	9114.83
$a(stim) + v(stim) + T_{er}(stim)$	8722.27
$a(stim) + v(stim) + T_{er}(stim) + z(single)$	8722.14
$a(stim) + v(stim) + T_{er}(stim) + z(stim)$	8721.08
$a(stim) + v(stim) + T_{er}(stim) + z(stim) + st$	8632.29
$a(stim) + v(stim) + T_{er}(stim) + z(stim) + sz$	8690.91
$a(stim) + v(stim) + T_{er}(stim) + z(stim) + sv$	8689.85
$a(stim) + v(stim) + T_{er}(stim) + z(stim) + sv + st$	8633.83
$a(stim) + v(stim) + T_{er}(stim) + z(stim) + sv + sz$	8691.04
$a(stim) + v(stim) + T_{er}(stim) + z(stim) + st + sz$	8631.22
$a(stim) + v(stim) + T_{er}(stim) + z(stim) + sv + st + sz$	8635.84

Note: ‘single’ and ‘stim’ refer to parameters which are fixed or varying according to stimulus type (‘previously encoded’ or ‘distractor’ recognition images), respectively.

Supplementary Table 6. *N*-Back fMRI between-groups analysis: small volume TFCE-corrected summary of a priori regions of interest across individual task contrasts (Drug > Placebo).

Task contrast	Mask [a]	Cluster	Hemisphere / division [b]	Peak voxel coordinates (MNI)			Cluster size (voxels)	*t*-value (peak)	*p*-value (peak)
				X	Y	Z			
1 > 0-Back									
	DLPFC (medial)	1	Left	-34	38	30	12	3.681	0.0450
2 > 0-Back (primary)									
	DLPFC (medial)	1	Left	-32	34	26	77	3.585	0.0124
	Hippocampus	1	Right	38	-22	-14	20	4.059	0.0184
		2	Left	-34	-18	-26	12	4.465	0.0170
		3	Left	-14	-42	8	1	4.065	0.0486
	Substantia nigra	1	Left	-10	-16	-10	2	3.169	0.0254
	Basal forebrain	1	Left	-4	0	-4	11	4.106	0.0118
3 > 0-Back									
	DLPFC (medial)	1	Left	-34	40	30	7	3.150	0.0458

Note: Statistical testing via SVC permutation testing (TFCE-corrected, two-tailed). [a] *a priori* small-volume corrected masks were bilateral and/or contained all divisions. [b] Only clusters identified through between-groups permutation tests which remained significant following TFCE correction ($p < 0.05$) were included. Note: Table contains data for N=52 individuals.

Supplementary Table 7. Verbal working memory n-back task: between-groups whole-brain fMRI analysis

Task contrast	Cluster	Location(s) *	Hemisphere / division (peak)	Peak voxel coordinates (MNI) X	Y	Z	Cluster size (voxels)	*t*-value (peak)	*p*-value (peak)
1 > 0-Back									
	1	Caudate	Right	20	-2	32	53	4.698	0.0382
	2	Caudate	Right	22	18	16	45	4.157	0.0448
	3	Lingual gyrus	Left	-22	-48	-6	43	4.421	0.0438
	4	Cingulate sulcus	Left	-14	28	34	37	4.730	0.0410
	5	Paracingulate gyrus	Left	-6	10	42	27	4.373	0.0440
	6	Insular cortex	Right	40	10	4	15	4.061	0.0468
	7	Paracingulate gyrus	Right	16	10	38	9	4.365	0.0458
	8	Cerebellum (VI)	Right	32	-38	-36	8	4.597	0.0472
	9	Frontal operculum	Right	38	18	6	4	3.909	0.0492
	10	Cerebellum (VI)	Left	-30	-40	-36	1	4.289	0.0498
	11	Cerebellum (V)	Left	-18	-50	-16	1	4.102	0.0496
2 > 0-Back (primary)									
	1	Left medial DLPFC; bilateral hippocampus; right inferior frontal gyrus; bilateral frontal pole; bilateral caudate; bilateral thalamus; bilateral cerebellum; left substantia nigra; left pallidum; basal forebrain; left lingual gyrus; left stria terminalis; left precentral gyrus	Right	-12	-10	22	18421	5.034	0.0006
	2	Postcentral and anterior supramarginal gyri	Left	-60	-20	26	298	4.049	0.0282
	3	Insular cortex	Right	36	10	0	38	3.782	0.0422
	4	Inferior temporal gyrus	Left	-48	-30	-26	3	3.516	0.0494
3 > 0-Back									
	NA	--	--	--	--	--	--	--	--

Note: Statistical testing via TFCE-corrected permutation testing. Only clusters which remained significant following correction ($p < 0.05$, two-tailed) were included. *Cortical or subcortical regions falling within the significant cluster.

Supplementary Table 8. *n*-Back Task: Drift Diffusion Model comparison

Model specification	Deviance information criterion (DIC)
$a + v(single) + T_{er}$	5766.366
$a + v(split) + T_{er}$	1622.017
$a + v(split) + T_{er} + z$	1513.519
$a + v(single) + T_{er} + z(split)$	5588.073
$a + v(split) + T_{er} + trial\ var.$	1561.630
$a + v(split) + T_{er} + trial\ var.cond$[a]	1402.282

[a] trial variation parameters depending on task condition (*n*-back level).

Supplementary Table 9. Comparison between H_3R-weighted and unweighted associations between group-level effects

Relationship	Weighting	β	*p*-corr [1]	η_p^2 [95% CI]	*r*
Hippocampus Encoding β ~ Mam ↔ Hippocampus Connectivity	H_3R-weighted [2]	1.20	0.012	0.16 [0.01, 0.35]	0.40
	Non-weighted	0.68	0.10	0.08 [0.00, 0.26]	0.29
Trace persistence β ~ Mam ↔ Hippocampus Connectivity	H_3R-weighted	1.98	0.0078	0.17 [0.02, 0.37]	0.41
	Non-weighted	1.52	0.0060	0.18 [0.02, 0.37]	0.42
Drift rate v (previously encoded) ~ trace persistence β	H_3R-weighted	0.02	0.0184	0.11 [0.00, 0.28]	0.33
	Non-weighted	0.02	0.0226	0.10 [0.00, 0.28]	0.32
Decision policy a (previously encoded) ~ trace persistence β	H_3R-weighted	-0.0078	0.0033	0.19 [0.04, 0.38]	-0.44
	Non-weighted	-0.0077	0.0042	0.19 [0.03, 0.37]	-0.43
Drift rate v ~ Increasing Complexity β (*n*-back)	H_3R-weighted	0.004	0.0335	0.09 [0.00, 0.26]	0.30
	Non-weighted	0.006	0.0629	0.07 [0.00, 0.23]	0.26

Note: Analyses were guided by group-level effects reported in the primary analyses, for both fMRI analyses (clusters) and behavioural/computational data. [1] Bonferroni-Holm correction to adjust for multiple comparisons. [2] H_3R-weighted and unweighted connectivity values were compared with H_3R-weighted unweighted cluster values, respectively.

Supplementary Table 10. Side effects profile during acute administration period (pitolisant vs. placebo) – descriptive statistics and inferential analysis

Side effect domain	Pitolisant (*n*=28) M (S.D.)	Placebo (*n*=29) M (S.D.)		
			F-statistic [df]	*p*
**Agitation**				
Baseline, M (S.D.)	0.00 (0.00)	0.00 (0.00)	--	--
Follow-up, M (S.D.)	0.21 (0.50)	0.21 (0.56)	< 0.00 [1, 54]	0.958
**Anxiety**				
Baseline, M (S.D.)	0.21 (0.42)	0.21 (0.42)	--	--
Follow-up, M (S.D.)	0.14 (0.36)	0.14 (0.44)	< 0.00 [1,54]	0.961
**Appetite, decreased**				
Baseline, M (S.D.)	0.32 (0.72)	0.11 (0.31)	--	--
Follow-up, M (S.D.)	0.29 (0.66)	0.17 (0.60)	0.83 [1,54]	0.367
**Appetite, increased**				
Baseline, M (S.D.)	0.07 (0.26)	0.17 (0.26)	--	--
Follow-up, M (S.D.)	1.00 (1.05)	0.97 (0.94)	0.017 [1,54]	0.898
**Diarrhoea**				
Baseline, M (S.D.)	0.00 (0.00)	0.00 (0.00)	--	--
Follow-up, M (S.D.)	0.00 (0.00)	0.00 (0.00)	0.00 [1,54]	1.000
**Drowsiness/Fatigue**				
Baseline, M (S.D.)	0.14 (0.36)	0.17 (0.38)	--	--
Follow-up, M (S.D.)	1.32 (0.91)	1.25 (0.84)	0.24 [1,54]	0.629
**Dry Mouth**				
Baseline, M (S.D.)	0.14 (0.36)	0.10 (0.31)	--	--
Follow-up, M (S.D.)	0.54 (0.79)	0.24 (0.51)	2.577 [1,54]	0.102
**Indigestion**				
Baseline, M (S.D.)	0.00 (0.00)	0.00 (0.00)	--	--
Follow-up, M (S.D.)	0.00 (0.00)	0.03 (0.19)	0.97 [1,54]	0.330
**Nausea**				
Baseline, M (S.D.)	0.00 (0.00)	0.00 (0.00)	--	--
Follow-up, M (S.D.)	0.18 (0.48)	0.07 (0.26)	1.18 [1,54]	0.282
**Sweating**				
Baseline, M (S.D.)	0.18 (0.39)	0.10 (0.31)	--	--
Follow-up, M (S.D.)	0.14 (0.45)	0.03 (0.19)	1.59 [1,54]	0.213
**Tremors**				
Baseline, M (S.D.)	0.07 (0.26)	0.00 (0.00)	--	--
Follow-up, M (S.D.)	0.04 (0.19)	0.00 (0.00)	1.96 [1, 54]	0.167
**Upset stomach**				
Baseline, M (S.D.)	0.00 (0.00)	0.00 (0.00)	--	--
Follow-up, M (S.D.)	0.00 (0.00)	0.03 (0.19)	1.00 [1,54]	0.330
**Side Effects Aggregate**				
Baseline, M (S.D.)	1.21 (1.37)	0.90 (1.18)	--	--
Follow-up, M (S.D.)	3.86 (2.62)	3.17 (2.80)	0.94 [1,54]	0.337

Note: Inferential analysis via baseline-adjusted ANCOVA modelling (two-tailed) across allocation groups (active vs placebo). Table contains data for *N*=57 individuals (114 observations total). *N*=1 individuals were excluded from analyses due to data missingness (baseline).

Supplementary Table 11. Self-rated state anxiety, anhedonia and affect across allocation groups

	Pitolisant (*n*=28) M (S.D.)	Placebo (*n*=29) M (S.D.)	Inferential analysis	
			F-statistic [df]	*p*
Dimensional Anhedonia Rating Scale				
Baseline, M (S.D.)	59.07 (4.97)	57.41 (7.53)	--	--
Follow-up, M (S.D.)	58.21 (8.54)	55.79 (8.60)	1.83 [1,54]	0.182
Positive and Negative Affect Schedule				
Negative items, baseline, M (S.D.)	11.68 (3.24)	10.90 (1.35)	--	--
Negative items, follow-up, M (S.D.)	12.89 (3.46)	11.90 (3.58)	1.43 [1,54]	0.238
Positive items, baseline, M (S.D.)	33.32 (6.63)	33.03 (7.84)	--	--
Positive items, follow-up, M (S.D.)	33.00 (7.14)	30.86 (8.37)	2.11 [1,54]	0.152
Spielberger State Anxiety Subscale				
Baseline, M (S.D.)	27.50 (5.41)	27.90 (6.14)	--	--
Follow-up, M (S.D.)	29.82 (7.59)	29.31 (9.29)	0.08 [1,54]	0.777
Visual Analogue Scale				
Negative items, baseline, M (S.D.)	143.46 (85.36)	123.76 (82.30)	--	--
Negative items, follow-up, M (S.D.)	220.50 (131.00)	225.28 (125.90)	0.03 [1,54]	0.872
Positive items, baseline, M (S.D.)	725.79 (98.20)	744.48 (97.45)	--	--
Positive items, follow-up, M (S.D.)	630.79 (180.19)	647.17 (142.01)	0.20 [1,54]	0.658

Note: Inferential analysis via baseline-adjusted ANCOVA modelling (two-tailed) across allocation groups (active vs placebo). Table contains data for *N*=57 individuals (114 observations total). *N*=1 individuals were excluded from analyses due to data missingness (baseline).

Supplementary Table 12. Mixed-effects linear modelling of subjective cognition throughout the acute assessment period

	Model estimate [a]	95% CI	*t* value	*p*
Motivation subscale				
Time	0.88	-0.02, 1.79	1.92	0.056
Allocation	2.56	-2.17, 7.28	1.06	0.291
Time × allocation	-0.67	-1.93, 0.59	-1.04	0.301
Mood subscale				
Time	-1.55	-2.40, -0.69	-3.55	0.0005
Allocation	1.82	-2.09, 5.74	0.91	0.363
Time × allocation	-0.34	-1.53, 0.85	-0.56	0.577
Executive function subscale				
Time	-0.78	-2.69, 1.13	-0.80	0.427
Allocation	5.30	-5.61, 16.21	0.95	0.343
Time × allocation	-3.48	-6.15, -0.81	-2.55	0.0117 [b]
Selective attention subscale				
Time	-2.94	-5.00, -0.88	-2.80	0.006
Allocation	3.26	-6.88, 13.40	0.63	0.529
Time × allocation	-1.71	-4.59, 1.17	-1.16	0.247
Memory subscale				
Time	-3.46	-5.37, -1.56	-3.57	0.0005
Allocation	-1.45	-11.42, 8.50	-0.29	0.775
Time × allocation	-1.70	-4.36, 0.96	-1.25	0.212
Subjective cognition aggregate				
Time	-2.98	-4.49, -1.46	-3.85	0.0002
Allocation	3.19	-5.22, 11.60	0.74	0.459
Time × allocation	-1.66	-3.78, 0.46	-1.54	0.126

Note: Inferential analyses via mixed effects linear regression modelling (two-tailed). [a] Main effect of group (active vs placebo), time, and group × time interactions were restricted using maximum likelihood estimation. [b] While a significant time × group interaction was observed, planned comparisons EMMs showed that allocation groups did not significantly differ at any time point during the testing period (T_1 EMM = -2.82 ± 5.14, p = 0.585; T_2 EMM = 3.97 ± 5.16, p = 0.443; T_3 EMM = 2.83 ± 5.43, p = 0.604; T_4 EMM = 8.95 ± 5.14, p = 0.085). The SSCS was collected at multiple time points (baseline [T_1], pre-scan [T_2], post-scan [T_3], and visit end [T_4]) all of which were included in the model. Table contains data for *N*=57 individuals (114 observations total). *N*=1 individuals were excluded from analyses due to data missingness (baseline).

Supplementary Table 13. Correlation analysis across self-rated subjective cognition (SSCS) and objective task performance

SSCS Domain

	Executive Function		Memory Function		Attention		Mood		Motivation		SSCS Composite	
N-Back	*r*	*p**	*r*	*p*	*r*	*p*	*r*	*p*	*r*	*p*	*r*	*p*
**Accuracy %**	0.30	0.08	0.33	0.08	0.34	0.08	0.08	0.59	0.26	0.09	0.28	0.08
**Response time**	-0.17	0.52	-0.17	0.52	-0.05	0.72	-0.06	0.72	-0.19	0.52	-0.09	0.72
**Drift rate**	0.31	0.17	0.13	0.61	0.12	0.61	0.04	0.77	0.14	0.61	0.10	0.61
Encoding task												
**Accuracy %**	-0.03	0.94	0.10	0.94	0.07	0.94	-0.03	0.94	0.01	0.94	-0.01	0.94
**Response time**	-0.02	0.88	-0.12	0.66	0.13	0.66	-0.15	0.66	-0.19	0.66	-0.04	0.88
**Drift rate**	-0.17	0.81	0.06	0.81	-0.09	0.81	0.03	0.84	-0.11	0.81	-0.07	0.81
Instrumental learning												
**Accuracy %**	0.05	0.79	0.16	0.62	0.14	0.62	-0.13	0.62	0.12	0.79	0.04	0.79
**Response time**	-0.08	0.96	-0.10	0.96	0.03	0.96	-0.03	0.96	-0.13	0.96	0.01	0.96
**Learning rate**	0.08	0.86	0.10	0.87	0.03	0.89	0.02	0.89	-0.22	0.81	0.09	0.87
**Beta**	0.01	0.93	-0.05	0.87	0.09	0.87	-0.15	0.87	0.29	0.30	-0.08	0.87

Note: Inferential analysis via two-tailed Pearson's *r* correlations (two-tailed, FWE-corrected) between subjective cognition (SSCS) and objective performance in cognitive task measures (N-Back, encoding task, probabilistic instrumental learning task) and associated cognitive measures. Due to some missing data from the SSCS, this table contains data for *N*=47 individuals. *Adjusted significance values using false discovery rate correction.

Supplementary References

1. Spielberger, C. D., Gonzalez-Reigosa, F., Martinez-Urrutia, A., Natalicio, L. F. S. & Natalicio, D. S. The state-trait anxiety inventory. *Revista Interamericana de Psicologia/Interamerican journal of psychology* **5**, (1971).

2. Rizvi, S. J. *et al.* Development and validation of the Dimensional Anhedonia Rating Scale (DARS) in a community sample and individuals with major depression. *Psychiatry Res* **229**, 109–119 (2015).

3. Crawford, J. R. & Henry, J. D. The Positive and Negative Affect Schedule (PANAS): Construct validity, measurement properties and normative data in a large non-clinical sample. *British journal of clinical psychology* **43**, 245–265 (2004).

4. Ratcliff, R., Smith, P. L., Brown, S. D. & McKoon, G. Diffusion decision model: Current issues and history. *Trends Cogn Sci* **20**, 260–281 (2016).

5. Wiecki, T. V, Sofer, I. & Frank, M. J. HDDM: Hierarchical Bayesian estimation of the Drift-Diffusion Model in Python. *Front Neuroinform* **7**, (2013).

6. Patil, A., Huard, D. & Fonnesbeck, C. J. PyMC: Bayesian stochastic modelling in Python. *J Stat Softw* **35**, 1 (2010).

7. Kumar, R., Carroll, C., Hartikainen, A. & Martin, O. ArviZ a unified library for exploratory analysis of Bayesian models in Python. *J Open Source Softw* **4**, 1143 (2019).

8. Spiegelhalter, D. J., Best, N. G., Carlin, B. P. & Van Der Linde, A. Bayesian measures of model complexity and fit. *J R Stat Soc Series B Stat Methodol* **64**, 583–639 (2002).

9. Ratcliff, R., Thapar, A. & McKoon, G. A diffusion model analysis of the effects of aging on recognition memory. *J Mem Lang* **50**, 408–424 (2004).

10. Pedersen, M. L. *et al.* Computational modeling of the N-Back task in the ABCD study: associations of drift diffusion model parameters to polygenic scores of mental disorders and cardiometabolic diseases. *Biol Psychiatry Cogn Neurosci Neuroimaging* **8**, 290–299 (2023).

11. van Ravenzwaaij, D., Donkin, C. & Vandekerckhove, J. The EZ diffusion model provides a powerful test of simple empirical effects. *Psychon Bull Rev* **24**, 547–556 (2017).

12. Colwell, M. J. *et al.* Direct serotonin release in humans shapes aversive learning and inhibition. *Nat Commun* **15**, 6617 (2024).

13. Gillespie, A. L., Wigg, C., Van Assche, I., Murphy, S. E. & Harmer, C. J. Associations Between Statin Use and Negative Affective Bias During COVID-19: An Observational, Longitudinal UK Study Investigating Depression Vulnerability. *Biol Psychiatry* **92**, 543–551 (2022).

14. Halahakoon, D. C. *et al.* Pramipexole Enhances Reward Learning by Preserving Value Estimates. *Biol Psychiatry* https://doi.org/https://doi.org/10.1016/j.biopsych.2023.05.023 (2023) doi:https://doi.org/10.1016/j.biopsych.2023.05.023.

15. Pessiglione, M., Seymour, B., Flandin, G., Dolan, R. J. & Frith, C. D. Dopamine-dependent prediction errors underpin reward-seeking behaviour in humans. *Nature* **442**, 1042–1045 (2006).

16. Browning, M., Paulus, M. & Huys, Q. J. M. What is computational psychiatry good for? *Biol Psychiatry* **93**, 658–660 (2023).

17. Chappell, M. A., Groves, A. R., Whitcher, B. & Woolrich, M. W. Variational Bayesian inference for a nonlinear forward model. *IEEE Transactions on Signal Processing* **57**, 223–236 (2008).

18. Chappell, M. A. *et al.* Partial volume correction of multiple inversion time arterial spin labeling MRI data. *Magn Reson Med* **65**, 1173–1183 (2011).

19. Philiastides, M. G., Auksztulewicz, R., Heekeren, H. R. & Blankenburg, F. Causal role of dorsolateral prefrontal cortex in human perceptual decision making. *Current biology* **21**, 980–983 (2011).

20. Georgiev, D. *et al.* Continuous theta burst stimulation over the dorsolateral prefrontal cortex and the pre-SMA alter drift rate and response thresholds respectively during perceptual decision-making. *Brain Stimul* **9**, 601–608 (2016).

21. Lin, J.-S. *et al.* An inverse agonist of the histamine H3 receptor improves wakefulness in narcolepsy: studies in orexin−/− mice and patients. *Neurobiol Dis* **30**, 74–83 (2008).

22. Schwartz, J. The histamine H3 receptor: from discovery to clinical trials with pitolisant. *Br J Pharmacol* **163**, 713–721 (2011).

23. Szakacs, Z. *et al.* Safety and efficacy of pitolisant on cataplexy in patients with narcolepsy: a randomised, double-blind, placebo-controlled trial. *Lancet Neurol* **16**, 200–207 (2017).

24. Stahl, S. M. *Stahl's Essential Psychopharmacology: Neuroscientific Basis and Practical Applications*. (Cambridge university press, 2021).

25. Gondard, E. *et al.* Enhanced Histaminergic Neurotransmission and Sleep-Wake Alterations, a Study in Histamine H3-Receptor Knock-Out Mice. *Neuropsychopharmacology* **38**, 1015–1031 (2013).

26. Toyota, H. *et al.* Behavioral characterization of mice lacking histamine H3 receptors. *Mol Pharmacol* **62**, 389–397 (2002).

27. Nuutinen, S. *et al.* Evidence for the Role of Histamine H3 Receptor in Alcohol Consumption and Alcohol Reward in Mice. *Neuropsychopharmacology* **36**, 2030–2040 (2011).

28. Ligneau, X. *et al.* BF2. 649 [1-{3-[3-(4-Chlorophenyl) propoxy] propyl} piperidine, hydrochloride], a nonimidazole inverse agonist/antagonist at the human histamine H3 receptor: Preclinical pharmacology. *Journal of Pharmacology and Experimental Therapeutics* **320**, 365–375 (2007).

29. Anichtchik, O. V, Peitsaro, N., Rinne, J. O., Kalimo, H. & Panula, P. Distribution and Modulation of Histamine H3 Receptors in Basal Ganglia and Frontal Cortex of Healthy Controls and Patients with Parkinson's Disease. *Neurobiol Dis* **8**, 707–716 (2001).

30. Brown, R. E., Stevens, D. R. & Haas, H. L. The physiology of brain histamine. *Prog Neurobiol* **63**, 637–672 (2001).

31. Shinohara, F., Arakaki, S., Amano, T., Minami, M. & Kaneda, K. Noradrenaline enhances the excitatory effects of dopamine on medial prefrontal cortex pyramidal neurons in rats. *Neuropsychopharmacol Rep* **40**, 348–354 (2020).

32. Lee, M. *et al.* Relationship between dopamine deficit and the expression of depressive behavior resulted from alteration of serotonin system. *Synapse* **69**, 453–460 (2015).

33. Woolley, D. W. Exploration of the Interrelationship of Serotonin with the Epinephrines and Histamine. *Proceedings of the National Academy of Sciences* **46**, 923–929 (1960).

34. Schlicker, E., Fink, K., Detzner, M. & Göthert, M. Histamine inhibits dopamine release in the mouse striatum via presynaptic H 3 receptors. *Journal of Neural Transmission/General Section JNT* **93**, 1–10 (1993).

35. Sargin, D., Jeoung, H.-S., Goodfellow, N. M. & Lambe, E. K. Serotonin regulation of the prefrontal cortex: cognitive relevance and the impact of developmental perturbation. *ACS Chem Neurosci* **10**, 3078–3093 (2019).

36. Leibowitz, S. F., Weiss, G. F., Walsh, U. A. & Viswanath, D. Medial hypothalamic serotonin: role in circadian patterns of feeding and macronutrient selection. *Brain Res* **503**, 132–140 (1989).

37. Ligneau, X. *et al.* Brain histamine and schizophrenia: potential therapeutic applications of H3-receptor inverse agonists studied with BF2. 649. *Biochem Pharmacol* **73**, 1215–1224 (2007).

38. Gao, M., Dekker, M. E., Leurs, R. & Vischer, H. F. Pharmacological characterization of seven human histamine H3 receptor isoforms. *Eur J Pharmacol* **968**, 176450 (2024).

39. Gbahou, F., Rouleau, A. & Arrang, J. The histamine autoreceptor is a short isoform of the H3 receptor. *Br J Pharmacol* **166**, 1860–1871 (2012).

40. Morisset, S. *et al.* High constitutive activity of native H3 receptors regulates histamine neurons in brain. *Nature* **408**, 860–864 (2000).

41. Bongers, G., Bakker, R. A. & Leurs, R. Molecular aspects of the histamine H3 receptor. *Biochem Pharmacol* **73**, 1195–1204 (2007).
