# Supplementary material for: Histamine shapes the neurocomputational dynamics of human learning
Source: Nat Commun. 2026 Jun 2;17:7124. doi: 10.1038/s41467-026-73865-9 (PMC13396361; doi:10.1038/s41467-026-73865-9)
Supplement: Supplementary file 2 — Reporting Summary [file 41467_2026_73865_MOESM2_ESM.pdf]

nature portfolio

Corresponding author(s): Dr Michael J Colwell

Last updated by author(s): 30/09/2025

Reporting Summary

Nature Portfolio wishes to improve the reproducibility of the work that we publish. This form provides structure for consistency and transparency in reporting. For further information on Nature Portfolio policies, see our Editorial Policies and the Editorial Policy Checklist.

Statistics

For all statistical analyses, confirm that the following items are present in the figure legend, table legend, main text, or Methods section.

n/a	Confirmed	
☐	☒	The exact sample size (*n*) for each experimental group/condition, given as a discrete number and unit of measurement
☐	☒	A statement on whether measurements were taken from distinct samples or whether the same sample was measured repeatedly
☐	☒	The statistical test(s) used AND whether they are one- or two-sided<br>*Only common tests should be described solely by name; describe more complex techniques in the Methods section.*
☐	☒	A description of all covariates tested
☐	☒	A description of any assumptions or corrections, such as tests of normality and adjustment for multiple comparisons
☐	☒	A full description of the statistical parameters including central tendency (e.g. means) or other basic estimates (e.g. regression coefficient) AND variation (e.g. standard deviation) or associated estimates of uncertainty (e.g. confidence intervals)
☐	☒	For null hypothesis testing, the test statistic (e.g. *F*, *t*, *r*) with confidence intervals, effect sizes, degrees of freedom and *P* value noted<br>*Give P values as exact values whenever suitable.*
☐	☒	For Bayesian analysis, information on the choice of priors and Markov chain Monte Carlo settings
☒	☐	For hierarchical and complex designs, identification of the appropriate level for tests and full reporting of outcomes
☐	☒	Estimates of effect sizes (e.g. Cohen's *d*, Pearson's *r*), indicating how they were calculated

Our web collection on statistics for biologists contains articles on many of the points above.

Software and code

Policy information about availability of computer code

Data collection	PsychoPy 2021.1.4; Anaconda 2021.4; MATLAB 2021a; Psychtoolbox 3; Presentation (Neurobs) 23.0; Qualtrics Surveys
Data analysis	The code used to undertake preprocessing, network analysis, computational modelling and inferential modelling are available on Zenodo (https://zenodo.org/records/19861591) and Github (https://github.com/mjcolwell/Histamine_Learning_Data_and_Code).<br><br>MATLAB (R2022a); Python (version 3.8.8) The following R packages were used: dplyr (1.1.2), tidyverse (2.0.0), gtools (3.9.4), knitr (1.42), data.table (1.14.8), ggplot2 (3.4.2), car (3.1-2), ggbeeswarm (0.7.2), ggrepel (0.9.3), readxl (1.4.2), openxlsx (4.2.5.2), ggpubr (0.6.0), rstatix (0.7.2), ez (4.4-0), ggsignif (0.6.4), RColorBrewer (1.1-3), emmeans (1.8.5), plotrix (3.8-2), sdamr (0.2.0), cowplot (1.1.1), psycho (0.6.1), ggridges (0.5.4), viridis (0.6.4), ggstance (0.3.6), ggdist (3.3.0), gghalves (0.1.4), ggpp (0.5.4), lme4 (1.1-33), stringr (1.5.0), effectsize (0.8.6), lmerTest (3.1-3), ggExtra (0.10.1), and moments (0.14.1). Neuroimaging analysis was primary undertaken using FSL (version 6.00), with additional imaging undertaken using the following python packages: Nibabel (v 5.2.1), Nilearn (v 0.10.4).

For manuscripts utilizing custom algorithms or software that are central to the research but not yet described in published literature, software must be made available to editors and reviewers. We strongly encourage code deposition in a community repository (e.g. GitHub). See the Nature Portfolio guidelines for submitting code & software for further information.

Data

Policy information about availability of data

All manuscripts must include a data availability statement. This statement should provide the following information, where applicable:

- Accession codes, unique identifiers, or web links for publicly available datasets
- A description of any restrictions on data availability
- For clinical datasets or third party data, please ensure that the statement adheres to our policy

The raw and modelled data generated for this study have been deposited on Zenodo (https://zenodo.org/records/19861591) and Github (https://github.com/mjcolwell/Histamine_Learning_Data_and_Code).

Research involving human participants, their data, or biological material

Policy information about studies with human participants or human data. See also policy information about sex, gender (identity/presentation), and sexual orientation and race, ethnicity and racism.

Reporting on sex and gender	Study participants were requested to self-identify their gender. This information was used to characterise the sample. Groups were balanced for gender during randomisation via a variance minimisation procedure. Gender-disaggregated analyses were not conducted given the study was not sufficiently powered to detect gender differences in the primary outcomes
Reporting on race, ethnicity, or other socially relevant groupings	Study participants were asked about educational background, first spoken language, and family history of mental health issues. This information was used to characterise the sample, and did not inform the primary analyses.
Population characteristics	Of the final sample (N=58), the mean age 28.18, and 64% were female. Behavioural, computational and neuroimaging data for tasks during the multi-stage memory paradigm consisted of N = 52 individuals (mean age = 28.58; 33 female), while resting state neuroimaging data consisted of N = 52 individuals (mean age = 28.39; 35 female). Behavioural, computational and neuroimaging data for the complex working memory task consisted of N = 52 individuals (mean age = 28.12; 33 female). Behavioural and computational data for the instrumental learning task consisted of N = 57 individuals (mean age = 28.16; 36 female).
Recruitment	Study participants were recruited via online (CallForParticipants; Meta/Facebook advertisements) and local (leaflets on noticeboards) advertisements. Prior to the study, participants provided informed consent. Participants were reimbursed 140 GBP upon completion of the study.
Ethics oversight	The study was approved by the University of Oxford Central University Research Ethics Committee (MSD-IREC reference code: R83940/RE002).

Note that full information on the approval of the study protocol must also be provided in the manuscript.

Field-specific reporting

Please select the one below that is the best fit for your research. If you are not sure, read the appropriate sections before making your selection.

☐ Life sciences ☒ Behavioural & social sciences ☐ Ecological, evolutionary & environmental sciences

For a reference copy of the document with all sections, see nature.com/documents/nr-reporting-summary-flat.pdf

Behavioural & social sciences study design

All studies must disclose on these points even when the disclosure is negative.

Study description	A quantitative research study involving a drug intervention in healthy adults with no mental or physical health conditions and not currently using psychoactive medication.
Research sample	A total of sixty participants were randomly assigned to receive either the drug or placebo. Two drop-outs occurred post-randomisation due to technical failure (scanner coil rupture; n=1) and withdrawal prior to drug/placebo administration (n=1). The final sample included 58 participants. Details of participant flow are presented in the CONSORT diagram in the Supplementary Materials (Supplementary Fig. 1). Statistical tests were conducted to confirm homogeneity of demographic variables across allocation groups. Prior to participation, all individuals underwent full medical screening, including a Structured Clinical Interview for DSM Disorders.<br><br>The final sample consisted of N=58 individuals, the mean age 28.18, and 64% were female. This sample is representative of the local recruitment area, Oxford, which has a substantial population of younger students. All participants provided informed consent prior to study participation.
Sampling strategy	A random sampling strategy was employed. Participants were randomised to allocation groups using a variance minimisation algorithm which was optimised for balanced group allocation while accounting for two covariates: gender and digit span score (as an

	index of baseline cognitive function). This approach maximises statistical power and reduces the risk of spurious group differences arising from chance imbalances in the allocation procedure. ample size was determined a priori using G*Power, with a required sample of N=52 calculated for 80% power at an alpha level of 0.05 for two-tailed between-groups analyses. Allowing for attrition, 60 participants were recruited.<br><br>Participants were recruited via local advertisement, introducing healthy volunteer bias whereby volunteers may differ from the general population in health behaviours or cognitive function. MRI self-selection bias is also likely, as individuals with claustrophobia were excluded by self-selection. These biases are common to pharmaco-fMRI studies and are unlikely to have systematically influenced between-group comparisons given the double-blind randomised design
Data collection	Behavioural task data was collected across in-scanner and non-scanner settings. Inside the MRI scanner, behavioural data was collected on a laptop connected to the MRI scanner, which projected the laptop monitor onto a mirror within the scanner. Within the scanner, data was collected using a button box placed on the right-hand of the participant (all participants were right-handed). During scans, a researcher a radiographer was present. For further details on MRI data collection, see the MRI section below. Out of the scanner, behavioural data was collected on a laptop connected to an external keyboard and mouse in a quiet experimental setting. Behavioural tasks were run on either Psychopy or Presentation (Neurobs) software packages. Questionnaire data was collected on Qualtrics software. During non-scanner tasks and questionnaires, a researcher was present to help setup tasks and give instructions on how to perform them. Researchers were aware of the study hypotheses given the pre-registered nature of the study; however, allocation blinding was maintained throughout data collection and analysis via the double-blind design.
Timing	Data collection occurred from April 2023 until January 2024.
Data exclusions	Participants who withdrew before drug/placebo administration or whose sessions ended due to scanner failure were excluded from the final analysis (n=2). Datasets with motion exceeding 1.5 mm or with technical issues (e.g., scanner failure during time-dependent sequences) were excluded (N=6 across all functional sequences). One participant was excluded from non-scanner task (PILT) due to prior exposure to task materials.
Non-participation	Of the 325 individuals who completed online screening, 85 attended the in-person medical screening. The remainder either did not meet inclusion criteria (n = 146) or declined the invitation (n = 55). Among those attending the in-person screening, 60 were randomized to receive drug or placebo, while the rest were deemed ineligible (n = 9) or declined further participation (n = 4). Two further drop-outs took place following randomisation due to technical issues (scanner coil rupture; n=1) and withdrawal prior to drug/placebo administration (n=1).
Randomization	Randomisation was undertaken by research staff who were not involved in the research process (e.g., data collection). Randomised was achieved using a variance minimisation algorithm, allowing for equal allocation across groups while balancing across two covariates: gender and digit span score. The variance minisation algorithm is described in the following paper:<br><br>Sella, F., Raz, G. & Cohen Kadosh, R. When randomisation is not good enough: Matching groups in intervention studies. Psychon Bull Rev 28, 2085–2093 (2021).

Reporting for specific materials, systems and methods

We require information from authors about some types of materials, experimental systems and methods used in many studies. Here, indicate whether each material, system or method listed is relevant to your study. If you are not sure if a list item applies to your research, read the appropriate section before selecting a response.

Materials & experimental systems

n/a	Involved in the study	
☒	☐	Antibodies
☒	☐	Eukaryotic cell lines
☒	☐	Palaeontology and archaeology
☒	☐	Animals and other organisms
☐	☒	Clinical data
☒	☐	Dual use research of concern
☒	☐	Plants

Methods

n/a	Involved in the study	
☒	☐	ChIP-seq
☒	☐	Flow cytometry
☐	☒	MRI-based neuroimaging

Clinical data

Policy information about clinical studies

All manuscripts should comply with the ICMJE guidelines for publication of clinical research and a completed CONSORT checklist must be included with all submissions.

Clinical trial registration	NCT05849675
Study protocol	The version-controlled protocol document, first uploaded on March 29, 2023, along with subsequent changes, can be accessed here: https://osf.io/gkmc6 and here: https://cdn.clinicaltrials.gov/large-docs/75/NCT05849675/Prot_000.pdf
Data collection	Data were collected in a research laboratory within the Neurosciences Building, Warneford Hospital (Oxford). Recruitment was conducted from April 2023 to January 2024 with data collection spanning May 2020 to January 2024.

Outcomes	Primary and secondary outcomes, as registered in the clinical trial, are defined as changes in the drug group compared with placebo at follow-up:<br><br>• Primary outcome 1: BOLD signal levels during resting state fMRI sequence<br>• Primary outcome 2: BOLD signal levels during fMRI memory encoding task<br>• Primary outcome 3: BOLD signal levels during fMRI n-back task<br><br>• Secondary outcome 1: Optimal choice selection during loss and reward conditions in Probabilistic Instrumental Learning Task<br>• Secondary outcome 2: Number of inhibited 'no-go' responses during the affective Interference Go/No-Go Task performance<br>• Secondary outcome 3: Accuracy of target selection on the Colour Change Detection Task<br>• Secondary outcome 4: Accuracy of emotional labeling of facial expressions during the facial emotion recognition task<br>• Secondary outcome 5: Accuracy of target selection during n-back fMRI task<br>• Secondary outcome 6: Accuracy of stimuli labeling (novel or familiar) during fMRI memory encoding task<br><br>This study was conducted as experimental medicine research with healthy participants and is not formally categorised as a clinical trial.

Plants

Seed stocks	*Report on the source of all seed stocks or other plant material used. If applicable, state the seed stock centre and catalogue number. If plant specimens were collected from the field, describe the collection location, date and sampling procedures.*
Novel plant genotypes	*Describe the methods by which all novel plant genotypes were produced. This includes those generated by transgenic approaches, gene editing, chemical/radiation-based mutagenesis and hybridization. For transgenic lines, describe the transformation method, the number of independent lines analyzed and the generation upon which experiments were performed. For gene-edited lines, describe the editor used, the endogenous sequence targeted for editing, the targeting guide RNA sequence (if applicable) and how the editor was applied.*
Authentication	*Describe any authentication procedures for each seed stock used or novel genotype generated. Describe any experiments used to assess the effect of a mutation and, where applicable, how potential secondary effects (e.g. second site T-DNA insertions, mosiacism, off-target gene editing) were examined.*

Magnetic resonance imaging

Experimental design

Design type	Task; block-based design; resting state; structural/perfusion
Design specifications	The resting state sequence was 10m38s in length and occurred before task-based sequences. The learning/encoding task consisted of 12 blocks (6 novel; 6 familiar) each containing 8 stimuli. The n-back task consisted of 12 blocks with 3 blocks per task condition (4 task conditions: 0-back, 1-back, 2-back, and 3-back) and 10 stimuli per block. The asl/ perfusion sequence occurred after all functional sequences and lasted 5.34 minutes.
Behavioral performance measures	For task-based sequences, raw behavioural data included button selected (accuracy) and response time. These data were input to drift diffusion models of evidence accumulation.

Acquisition

Imaging type(s)	Structural; functional; perfusion/asl
Field strength	3
Sequence & imaging parameters	All MR images were acquired on 3-Tesla Prisma Siemens scanner utilising a 32-channel head matrix coil at the Oxford Centre for Human Brain Activity (University of Oxford). High resolution T1-weighted structural images were acquired using the following parameters: 192 slices; TR = 1900 ms, TE = 3.97 ms, FOV = 192 mm, flip angle = 8°, voxel size = 1 mm^3; acquisition time = 5:31. T2-weighted echoplanar images were acquired using the following parameters: TR = 590.0 ms; TE = 4.92 ms/7.38 ms (multband); FOV = 216 mm; flip angle = 46°; voxel size = $3mm^3$). Functional MRI data during the resting state sequence was acquired using the following parameters: TR = 1400 ms; TE = 30.00 ms; FOV = 216 mm; flip angle = 70°; voxel size = 2.40 mm^3; multiband acceleration factor = 3). Functional data during the memory/encoding task was acquiring using the following parameters: TR = 800ms, TE = 30ms, flip angle = 52°, slice thickness = 2mm, multiband accelerator factor 6, resolution = 2.4mm3 isotropic voxel size). Functional data during the complex working memory task (n-back) was acquired using the following parameters: TR = 1500ms, TE = 25ms, flip angle = 70°, slice thickness = 2mm, multiband accelerator factor 3, PAT GRAPPA acceleration factor 2, resolution = 2mm3 isotropic voxel size, acquisition time = 15 mins, 9 sec). Arterial Spin Labelling data was acquired with the following parameters: TR = 4100 ms; TE = 14.0 ms; flip angle = 90°; slice thickness = 4.5 mm. The phase encoding direction for all sequences was A >> P.
Area of acquisition	Whole brain; optimised for hippocampus imaging

Diffusion MRI ☐ Used ☒ Not used

Preprocessing

Preprocessing software	All MRI data was converted to NIfTI-1 using dcm2niix (v1.0.20220720) and BIDS specification using HeuDiConv (v 1.0.1). Image quality was assess using MRIQC (v23.1.0). Structural images were defaced using pydeface (v2.0.2). Skull-stripping for structural data was undertaken via synthstrip (v1.3). T Normalisation/registration of all images was undertaken via FSL's FLIRT/FNIRT (FSL 6.0.6; https://fsl.fmrib.ox.ac.uk/fsl/) . Functional resting state data underwent first-level preprocessing via FSL's MELODIC tool (including motion correction with MCFLIRT), and were denoised via FSL's ICA-FIX. Functional task data were preprocessed and analysed at the first level in FSL FEAT (including motion correction with MCFLIRT). ASL/perfusion data were normalised, distortion-corrected and motion-corrected via FSL's BASIL and FSL's FSL_Anat.
Normalization	All acquired images (functional/structural/ASL) and PET maps from neuromaps were registered to 2 mm MNI space using FSL's FLIRT/FNIRT tool.
Normalization template	MNI 152 2mm standard space T1-weighted template.
Noise and artifact removal	Motion correction for functional data was undertaken with FSL's MCFLIRT tool. Field-map correction for functional data was undertaken with FSL's FEAT tool. Motion outliers were identified via fsl_motion_outliers and included in the first-level GLM in FEAT for further motion removal. For all functional sequences, individuals who exhibited high motion (maximum absolute displacement of ≥ 1.5mm) were excluded from the final analysis. Functional resting state data was further denoised using FSL's ICA-FIX. The ICA-FIX model was trained to distinguish signal from noise using hand-labeled data from 16 participants from the present study, and cross-validated using the leave-one-out method. Functional data was underwent a high-pass filter and was smoothed to 2.5mm and 5mm Gaussian kernels for resting state and task-based data, respectively. Images were manually inspected for alignment issues or noise artifacts.
Volume censoring	N/A

Statistical modeling & inference

Model type and settings	First-level GLMs were generated using FSL's feat tool for all functional data. For all first-level GLMs, motion outliers were included as covariates within the model. In first-level GLMs for task-base analysis, further regressors were specified to represent task conditions or remove non-relevant task periods (e.g., instructions or end-task periods); the first 5 volumes of each were excluded from analysis.<br><br>For the memory task, first-level regressors included volumes marked as belonging to task blocks, including 'novel' stimuli blocks, 'familiar' stimuli' blocks, rest periods following 'novel' blocks, rest periods following 'familiar' blocks, and 'ending' and 'beginning' instructions periods. Activity during 'novel' and 'familiar' stimulus blocks were contrasted to attain a main effect of encoding. To analyse activity during encoding periods only, these regressors were convolved around the haemodynamic response function. To analyse persisting activity after encoding periods, information from 'novel' and 'familiar' rest blocks were decomposed using a finite impulse response function across 15 temporal bins (1 per 0.8 TR). These bins were concatenated within a linear model that captured signal decay, contrasting rest periods following novel versus familiar encoding (t_1 novel [EV –7.5] → t_{15} novel [EV +6.5]; t_1 familiar [EV +6.5] → t_{15} familiar [EV –7.5]).<br><br>For the complex working memory, first-level regressors included volumes marked as belong to task blocks, including each task condition ('0-back', '1-back', '2-back', '3-back), baseline/rest periods, and instruction periods. Task-related activity was modeled linearly with increasing load, with additional contrasts tested between each level and baseline (3 > 0-back, 2 > 0-back, 1 > 0-back). For all task-based analyses, inferential statistics were undertaken via FSL's randomise (set to 5000 permutations).<br><br>For the resting state analysis, a multivariate and univariate network analysis was undertaken with fslnets. Within this analysis, signals during rest were extract from a priori regions of interest (weighted and non-weighted by H3R); these signals were transformed into covariance matrices via fslnets. Multivariate (global connectivity) analyses across covariance matrices was undertaken using linear discriminant analysis (via scikit-learn). Additional univariate (5000 permutations) analysis of connectivity between each node in the matrix was undertaken via fslnets and corrected for FWE.<br><br>For ASL data analysis, regional CSF analysis was undertaken using FSL's randomise (5000 permutations) within whole-brain and task-network maps. For global grey and white matter analyses, CSF values from BASIL for grey and white matter were analysed with between-groups ANOVA modeling.
Effect(s) tested	To determine the main effect of new learning within the encoding task, fMRI activity was contrasted across 'novel' and 'familiar' blocks (novel > familiar; familiar > novel). For the main effect of signal persistence within post-learning rest blocks was determined using a similar contrast (novel rest > familiar rest; familiar rest > novel rest). For the complex working memory task, the effect of increasing task complex was achieved by linearly modeling activity within each task condition according to difficulty (0-back [EV –1.5] to 3-back [EV +1.5]), and pairwise comparisons between each difficulty level and the control condition were undertaken (3 > 0-back, 2 > 0-back, 1 > 0-back). For the univariate network analysis, connectivity between all network nodes was assessed. For ASL data, only between-groups analyses were undertaken.<br><br>For all contrasts (functional, network-based or CSF), the main effect of group was assessed: pitolisant > placebo; placebo > pitolisant.

Specify type of analysis: ☐ Whole brain ☐ ROI-based ☒ Both

For the resting state/network analysis and memory encoding task (effect of learning; signal persistence) the same a priori ROIs were used: hippocampus; basal forebrain; entorhinal cortex; perirhinal cortex; mammilary zone (an ROI containing voxels belonging to the TMN and mammilary bodies). For the

Anatomical location(s)	complex working memory task, a priori ROIs included the caudal and rostral dorsolateral prefrontal cortex, hippocampus, basal forebrain and substantia nigra. All bilateralised structures were a single ROI including voxels from both hemispheres.<br><br>All ROIs were derived from Jülich histological atlas, except outer cortical structures which were derived from the Human Connectome Project atlas. The perirhinal cortex ROI was created created using 5mm radius spheres.<br><br>All ROI masks are included in the task materials: https://github.com/mjcolwell/Histamine_Learning_Data_and_Code
Statistic type for inference<br>(See Eklund et al. 2016)	All inferential statistics (except network analysis) was undertaken using FSL's randomise (voxelwise permutation tests [5000 permutations]). Multivariate network analysis was undertaken on covariance matrices using linear discriminant analysis, and between-groups univariate analysis was undertaken using permutation testing.
Correction	For all voxelwise statistical inference, the threshold-free cluster enhancement correction was undertaken. For univariate network analysis, family-wise error was corrected using the maximal statistic method (via fsl's randomise). To further correct for multiple comparisons, where appropriate all ROIs were bilateralised/contained all divisions.

Models & analysis

n/a	Involved in the study
☐	☒ Functional and/or effective connectivity
☒	☐ Graph analysis
☒	☐ Multivariate modeling or predictive analysis

Functional and/or effective connectivity	For the network-based analysis (H3R weighted and non-weighted), partial correlation matrices were constructed from extracted resting-state time series. Regularised partial correlation matrices were then subjected to linear discriminant analysis. For the univariate analysis, individual edge values were treated as univariate data points in subsequent statistical testing.
